# EXMOTIF: efficient structured motif extraction

**DOI:** 10.1186/1748-7188-1-21

**Published:** 2006-11-16

**Authors:** Yongqiang Zhang, Mohammed J Zaki

**Affiliations:** 1 Department of Computer Science, Rensselaer Polytechnic Institute, Troy, New York 12180, USA

## Abstract

**Background:**

Extracting motifs from sequences is a mainstay of bioinformatics. We look at the problem of mining structured motifs, which allow variable length gaps between simple motif components. We propose an efficient algorithm, called EXMOTIF, that given some sequence(s), and a structured motif template, extracts all *frequent *structured motifs that have quorum *q*. Potential applications of our method include the extraction of single/composite regulatory binding sites in DNA sequences.

**Results:**

EXMOTIF is efficient in terms of both time and space and is shown empirically to outperform RISO, a state-of-the-art algorithm. It is also successful in finding potential single/composite transcription factor binding sites.

**Conclusion:**

EXMOTIF is a useful and efficient tool in discovering structured motifs, especially in DNA sequences. The algorithm is available as open-source at: .

## Introduction

Analyzing and interpreting sequence data is an important task in bioinformatics. One critical aspect of such interpretation is to extract important motifs (patterns) from sequences. The challenges for motif extraction problem are two-fold: one is to design an efficient algorithm to enumerate the frequent motifs; the other is to statistically validate the extracted motifs and report the significant ones.

Motifs can be classified into two main types. If no variable gaps are allowed in the motif, it is called a *simple motif*. For example, in the genome of *Saccharomyces cerevisiae*, the binding sites of transcription factor, GAL4, have as consensus [1], the simple motif, CGG[11,11]CCG. Here [11,11] means that there is a fixed "gap" (or don't care characters), 11 positions long. If variable gaps are allowed in a motif, it is called a *structured motif*. A structured motif can be regarded as an ordered collection of simple motifs with gap constraints between each pair of adjacent simple motifs. For example, many *retrotransposons *in the *Ty1-copia *group [2] have as consensus the structured motif:  MT[115,136]MTNTAYGG[121,151]GTNGAYGAY. Here MT, MTNTAYGG and GTNGAYGAY are three simple motifs; [115,136] and [121,151] are variable gap constraints ([minimum gap, maximum gap]) allowed between the adjacent simple motifs. More formally, a structured motif, ℳ
 MathType@MTEF@5@5@+=feaafiart1ev1aaatCvAUfKttLearuWrP9MDH5MBPbIqV92AaeXatLxBI9gBamrtHrhAL1wy0L2yHvtyaeHbnfgDOvwBHrxAJfwnaebbnrfifHhDYfgasaacH8akY=wiFfYdH8Gipec8Eeeu0xXdbba9frFj0=OqFfea0dXdd9vqai=hGuQ8kuc9pgc9s8qqaq=dirpe0xb9q8qiLsFr0=vr0=vr0dc8meaabaqaciaacaGaaeqabaWaaeGaeaaakeaaimaacqWFZestaaa@3790@, is specified in the form:

*M*_1_[*l*_1_, *u*_1_]*M*_2_[*l*_2_, *u*_2_]*M*_3 _... *M*_*k*-1_[*l*_*k*-1_, *u*_*k*-1_]*M*_*k*_

where *M*_*i*_, 1 ≤ *i *≤ *k*, is a simple motif *component*, and *l*_*i *_and *u*_*i *_(for 1 ≤ *i *<*k *and where 0 ≤ *l*_*i *_≤ *u*_*i*_), are the minimum and maximum number of gaps allowed between *M*_*i *_and *M*_*i*+1_, respectively. Note that a gap is defined to be the number of intervening positions after *M*_*i *_but before *M*_*i*+1_. In other words, if *s*_*i *_and *e*_*i *_represent the start and end positions of component *M*_*i*_, then for *i *∈ [1, *k *- 1], the number of gaps is given as *g*_*i *_= *e*_*i*+1 _- *s*_*i *_- 1, and we require that *g*_*i *_∈ [*l*_*i*_, *u*_*i*_]. The number of simple motif components, *k*, is also called the *length *of ℳ
 MathType@MTEF@5@5@+=feaafiart1ev1aaatCvAUfKttLearuWrP9MDH5MBPbIqV92AaeXatLxBI9gBamrtHrhAL1wy0L2yHvtyaeHbnfgDOvwBHrxAJfwnaebbnrfifHhDYfgasaacH8akY=wiFfYdH8Gipec8Eeeu0xXdbba9frFj0=OqFfea0dXdd9vqai=hGuQ8kuc9pgc9s8qqaq=dirpe0xb9q8qiLsFr0=vr0=vr0dc8meaabaqaciaacaGaaeqabaWaaeGaeaaakeaaimaacqWFZestaaa@3790@. Let *W*_*i*_, 1 ≤ *i *<*k*, denote the span of the gap range, [*l*_*i*_, *u*_*i*_], which is calculated as: *W*_*i *_= *u*_*i *_- *l*_*i *_+ 1.

In the structured motif extraction problem, the component motifs *M*_*i *_are *unknown *before the extraction. However, we do provide some *known *parameters to restrict the structured motifs to be extracted, including: (i) *k *– the *length *of ℳ
 MathType@MTEF@5@5@+=feaafiart1ev1aaatCvAUfKttLearuWrP9MDH5MBPbIqV92AaeXatLxBI9gBamrtHrhAL1wy0L2yHvtyaeHbnfgDOvwBHrxAJfwnaebbnrfifHhDYfgasaacH8akY=wiFfYdH8Gipec8Eeeu0xXdbba9frFj0=OqFfea0dXdd9vqai=hGuQ8kuc9pgc9s8qqaq=dirpe0xb9q8qiLsFr0=vr0=vr0dc8meaabaqaciaacaGaaeqabaWaaeGaeaaakeaaimaacqWFZestaaa@3790@; (ii) |*M*_*i*_| – the length of each component *M*_*i *_∈ ℳ
 MathType@MTEF@5@5@+=feaafiart1ev1aaatCvAUfKttLearuWrP9MDH5MBPbIqV92AaeXatLxBI9gBamrtHrhAL1wy0L2yHvtyaeHbnfgDOvwBHrxAJfwnaebbnrfifHhDYfgasaacH8akY=wiFfYdH8Gipec8Eeeu0xXdbba9frFj0=OqFfea0dXdd9vqai=hGuQ8kuc9pgc9s8qqaq=dirpe0xb9q8qiLsFr0=vr0=vr0dc8meaabaqaciaacaGaaeqabaWaaeGaeaaakeaaimaacqWFZestaaa@3790@, for 1 ≤ *i *≤ *k*; and (iii) [*l*_*i*_, *u*_*i*_] – the gap range between *M*_*i *_and *M*_*i*+1_, for 1 ≤ *i *<*k*. All these parameters define a *structured motif template*, T
 MathType@MTEF@5@5@+=feaafiart1ev1aaatCvAUfKttLearuWrP9MDH5MBPbIqV92AaeXatLxBI9gBamrtHrhAL1wy0L2yHvtyaeHbnfgDOvwBHrxAJfwnaebbnrfifHhDYfgasaacH8akY=wiFfYdH8Gipec8Eeeu0xXdbba9frFj0=OqFfea0dXdd9vqai=hGuQ8kuc9pgc9s8qqaq=dirpe0xb9q8qiLsFr0=vr0=vr0dc8meaabaqaciaacaGaaeqabaWaaeGaeaaakeaaimaacqWFtepvaaa@3847@, for the structured motifs to be extracted from a set of sequences S
 MathType@MTEF@5@5@+=feaafiart1ev1aaatCvAUfKttLearuWrP9MDH5MBPbIqV92AaeXatLxBI9gBamrtHrhAL1wy0L2yHvtyaeHbnfgDOvwBHrxAJfwnaebbnrfifHhDYfgasaacH8akY=wiFfYdH8Gipec8Eeeu0xXdbba9frFj0=OqFfea0dXdd9vqai=hGuQ8kuc9pgc9s8qqaq=dirpe0xb9q8qiLsFr0=vr0=vr0dc8meaabaqaciaacaGaaeqabaWaaeGaeaaakeaaimaacqWFse=uaaa@3845@. A structured motif ℳ
 MathType@MTEF@5@5@+=feaafiart1ev1aaatCvAUfKttLearuWrP9MDH5MBPbIqV92AaeXatLxBI9gBamrtHrhAL1wy0L2yHvtyaeHbnfgDOvwBHrxAJfwnaebbnrfifHhDYfgasaacH8akY=wiFfYdH8Gipec8Eeeu0xXdbba9frFj0=OqFfea0dXdd9vqai=hGuQ8kuc9pgc9s8qqaq=dirpe0xb9q8qiLsFr0=vr0=vr0dc8meaabaqaciaacaGaaeqabaWaaeGaeaaakeaaimaacqWFZestaaa@3790@ matching the template T
 MathType@MTEF@5@5@+=feaafiart1ev1aaatCvAUfKttLearuWrP9MDH5MBPbIqV92AaeXatLxBI9gBamrtHrhAL1wy0L2yHvtyaeHbnfgDOvwBHrxAJfwnaebbnrfifHhDYfgasaacH8akY=wiFfYdH8Gipec8Eeeu0xXdbba9frFj0=OqFfea0dXdd9vqai=hGuQ8kuc9pgc9s8qqaq=dirpe0xb9q8qiLsFr0=vr0=vr0dc8meaabaqaciaacaGaaeqabaWaaeGaeaaakeaaimaacqWFtepvaaa@3847@ in S
 MathType@MTEF@5@5@+=feaafiart1ev1aaatCvAUfKttLearuWrP9MDH5MBPbIqV92AaeXatLxBI9gBamrtHrhAL1wy0L2yHvtyaeHbnfgDOvwBHrxAJfwnaebbnrfifHhDYfgasaacH8akY=wiFfYdH8Gipec8Eeeu0xXdbba9frFj0=OqFfea0dXdd9vqai=hGuQ8kuc9pgc9s8qqaq=dirpe0xb9q8qiLsFr0=vr0=vr0dc8meaabaqaciaacaGaaeqabaWaaeGaeaaakeaaimaacqWFse=uaaa@3845@ is called an *instance *of T
 MathType@MTEF@5@5@+=feaafiart1ev1aaatCvAUfKttLearuWrP9MDH5MBPbIqV92AaeXatLxBI9gBamrtHrhAL1wy0L2yHvtyaeHbnfgDOvwBHrxAJfwnaebbnrfifHhDYfgasaacH8akY=wiFfYdH8Gipec8Eeeu0xXdbba9frFj0=OqFfea0dXdd9vqai=hGuQ8kuc9pgc9s8qqaq=dirpe0xb9q8qiLsFr0=vr0=vr0dc8meaabaqaciaacaGaaeqabaWaaeGaeaaakeaaimaacqWFtepvaaa@3847@. We use *K *to denote the number of symbols (not counting gaps) in ℳ
 MathType@MTEF@5@5@+=feaafiart1ev1aaatCvAUfKttLearuWrP9MDH5MBPbIqV92AaeXatLxBI9gBamrtHrhAL1wy0L2yHvtyaeHbnfgDOvwBHrxAJfwnaebbnrfifHhDYfgasaacH8akY=wiFfYdH8Gipec8Eeeu0xXdbba9frFj0=OqFfea0dXdd9vqai=hGuQ8kuc9pgc9s8qqaq=dirpe0xb9q8qiLsFr0=vr0=vr0dc8meaabaqaciaacaGaaeqabaWaaeGaeaaakeaaimaacqWFZestaaa@3790@ and use ℳ
 MathType@MTEF@5@5@+=feaafiart1ev1aaatCvAUfKttLearuWrP9MDH5MBPbIqV92AaeXatLxBI9gBamrtHrhAL1wy0L2yHvtyaeHbnfgDOvwBHrxAJfwnaebbnrfifHhDYfgasaacH8akY=wiFfYdH8Gipec8Eeeu0xXdbba9frFj0=OqFfea0dXdd9vqai=hGuQ8kuc9pgc9s8qqaq=dirpe0xb9q8qiLsFr0=vr0=vr0dc8meaabaqaciaacaGaaeqabaWaaeGaeaaakeaaimaacqWFZestaaa@3790@[*j*] (with 1 ≤ *j *≤ *K*) to denote the *j*th symbol of ℳ
 MathType@MTEF@5@5@+=feaafiart1ev1aaatCvAUfKttLearuWrP9MDH5MBPbIqV92AaeXatLxBI9gBamrtHrhAL1wy0L2yHvtyaeHbnfgDOvwBHrxAJfwnaebbnrfifHhDYfgasaacH8akY=wiFfYdH8Gipec8Eeeu0xXdbba9frFj0=OqFfea0dXdd9vqai=hGuQ8kuc9pgc9s8qqaq=dirpe0xb9q8qiLsFr0=vr0=vr0dc8meaabaqaciaacaGaaeqabaWaaeGaeaaakeaaimaacqWFZestaaa@3790@.

Let *δ*_*S *_(ℳ
 MathType@MTEF@5@5@+=feaafiart1ev1aaatCvAUfKttLearuWrP9MDH5MBPbIqV92AaeXatLxBI9gBamrtHrhAL1wy0L2yHvtyaeHbnfgDOvwBHrxAJfwnaebbnrfifHhDYfgasaacH8akY=wiFfYdH8Gipec8Eeeu0xXdbba9frFj0=OqFfea0dXdd9vqai=hGuQ8kuc9pgc9s8qqaq=dirpe0xb9q8qiLsFr0=vr0=vr0dc8meaabaqaciaacaGaaeqabaWaaeGaeaaakeaaimaacqWFZestaaa@3790@) denote the number of occurrences of an instance motif ℳ
 MathType@MTEF@5@5@+=feaafiart1ev1aaatCvAUfKttLearuWrP9MDH5MBPbIqV92AaeXatLxBI9gBamrtHrhAL1wy0L2yHvtyaeHbnfgDOvwBHrxAJfwnaebbnrfifHhDYfgasaacH8akY=wiFfYdH8Gipec8Eeeu0xXdbba9frFj0=OqFfea0dXdd9vqai=hGuQ8kuc9pgc9s8qqaq=dirpe0xb9q8qiLsFr0=vr0=vr0dc8meaabaqaciaacaGaaeqabaWaaeGaeaaakeaaimaacqWFZestaaa@3790@ in a sequence *S *∈ S
 MathType@MTEF@5@5@+=feaafiart1ev1aaatCvAUfKttLearuWrP9MDH5MBPbIqV92AaeXatLxBI9gBamrtHrhAL1wy0L2yHvtyaeHbnfgDOvwBHrxAJfwnaebbnrfifHhDYfgasaacH8akY=wiFfYdH8Gipec8Eeeu0xXdbba9frFj0=OqFfea0dXdd9vqai=hGuQ8kuc9pgc9s8qqaq=dirpe0xb9q8qiLsFr0=vr0=vr0dc8meaabaqaciaacaGaaeqabaWaaeGaeaaakeaaimaacqWFse=uaaa@3845@. Let *d*_*S *_(ℳ
 MathType@MTEF@5@5@+=feaafiart1ev1aaatCvAUfKttLearuWrP9MDH5MBPbIqV92AaeXatLxBI9gBamrtHrhAL1wy0L2yHvtyaeHbnfgDOvwBHrxAJfwnaebbnrfifHhDYfgasaacH8akY=wiFfYdH8Gipec8Eeeu0xXdbba9frFj0=OqFfea0dXdd9vqai=hGuQ8kuc9pgc9s8qqaq=dirpe0xb9q8qiLsFr0=vr0=vr0dc8meaabaqaciaacaGaaeqabaWaaeGaeaaakeaaimaacqWFZestaaa@3790@) = 1 if *δ*_*S *_(ℳ
 MathType@MTEF@5@5@+=feaafiart1ev1aaatCvAUfKttLearuWrP9MDH5MBPbIqV92AaeXatLxBI9gBamrtHrhAL1wy0L2yHvtyaeHbnfgDOvwBHrxAJfwnaebbnrfifHhDYfgasaacH8akY=wiFfYdH8Gipec8Eeeu0xXdbba9frFj0=OqFfea0dXdd9vqai=hGuQ8kuc9pgc9s8qqaq=dirpe0xb9q8qiLsFr0=vr0=vr0dc8meaabaqaciaacaGaaeqabaWaaeGaeaaakeaaimaacqWFZestaaa@3790@) > 0 and *d*_*S *_(ℳ
 MathType@MTEF@5@5@+=feaafiart1ev1aaatCvAUfKttLearuWrP9MDH5MBPbIqV92AaeXatLxBI9gBamrtHrhAL1wy0L2yHvtyaeHbnfgDOvwBHrxAJfwnaebbnrfifHhDYfgasaacH8akY=wiFfYdH8Gipec8Eeeu0xXdbba9frFj0=OqFfea0dXdd9vqai=hGuQ8kuc9pgc9s8qqaq=dirpe0xb9q8qiLsFr0=vr0=vr0dc8meaabaqaciaacaGaaeqabaWaaeGaeaaakeaaimaacqWFZestaaa@3790@) = 0 if *δ*_*S *_(ℳ
 MathType@MTEF@5@5@+=feaafiart1ev1aaatCvAUfKttLearuWrP9MDH5MBPbIqV92AaeXatLxBI9gBamrtHrhAL1wy0L2yHvtyaeHbnfgDOvwBHrxAJfwnaebbnrfifHhDYfgasaacH8akY=wiFfYdH8Gipec8Eeeu0xXdbba9frFj0=OqFfea0dXdd9vqai=hGuQ8kuc9pgc9s8qqaq=dirpe0xb9q8qiLsFr0=vr0=vr0dc8meaabaqaciaacaGaaeqabaWaaeGaeaaakeaaimaacqWFZestaaa@3790@) = 0. The *support *of motif ℳ
 MathType@MTEF@5@5@+=feaafiart1ev1aaatCvAUfKttLearuWrP9MDH5MBPbIqV92AaeXatLxBI9gBamrtHrhAL1wy0L2yHvtyaeHbnfgDOvwBHrxAJfwnaebbnrfifHhDYfgasaacH8akY=wiFfYdH8Gipec8Eeeu0xXdbba9frFj0=OqFfea0dXdd9vqai=hGuQ8kuc9pgc9s8qqaq=dirpe0xb9q8qiLsFr0=vr0=vr0dc8meaabaqaciaacaGaaeqabaWaaeGaeaaakeaaimaacqWFZestaaa@3790@ in the is defined as π(ℳ)=∑S∈SdS(ℳ)
 MathType@MTEF@5@5@+=feaafiart1ev1aaatCvAUfKttLearuWrP9MDH5MBPbIqV92AaeXatLxBI9gBamrtHrhAL1wy0L2yHvtyaeHbnfgDOvwBHrxAJfwnaebbnrfifHhDYfgasaacH8akY=wiFfYdH8Gipec8Eeeu0xXdbba9frFj0=OqFfea0dXdd9vqai=hGuQ8kuc9pgc9s8qqaq=dirpe0xb9q8qiLsFr0=vr0=vr0dc8meaabaqaciaacaGaaeqabaWaaeGaeaaakeaaiiGacqWFapaCcqGGOaakimaacqGFZestcqGGPaqkcqGH9aqpdaaeqaqaaiabdsgaKnaaBaaaleaacqWGtbWuaeqaaaqaaiabdofatjabgIGiolab+jr8tbqab0GaeyyeIuoakiabcIcaOiab+ntinjabcMcaPaaa@47FD@, i.e., the number of sequences in S
 MathType@MTEF@5@5@+=feaafiart1ev1aaatCvAUfKttLearuWrP9MDH5MBPbIqV92AaeXatLxBI9gBamrtHrhAL1wy0L2yHvtyaeHbnfgDOvwBHrxAJfwnaebbnrfifHhDYfgasaacH8akY=wiFfYdH8Gipec8Eeeu0xXdbba9frFj0=OqFfea0dXdd9vqai=hGuQ8kuc9pgc9s8qqaq=dirpe0xb9q8qiLsFr0=vr0=vr0dc8meaabaqaciaacaGaaeqabaWaaeGaeaaakeaaimaacqWFse=uaaa@3845@ that contain at least one occurrence of ℳ
 MathType@MTEF@5@5@+=feaafiart1ev1aaatCvAUfKttLearuWrP9MDH5MBPbIqV92AaeXatLxBI9gBamrtHrhAL1wy0L2yHvtyaeHbnfgDOvwBHrxAJfwnaebbnrfifHhDYfgasaacH8akY=wiFfYdH8Gipec8Eeeu0xXdbba9frFj0=OqFfea0dXdd9vqai=hGuQ8kuc9pgc9s8qqaq=dirpe0xb9q8qiLsFr0=vr0=vr0dc8meaabaqaciaacaGaaeqabaWaaeGaeaaakeaaimaacqWFZestaaa@3790@. The *weighted support *of ℳ
 MathType@MTEF@5@5@+=feaafiart1ev1aaatCvAUfKttLearuWrP9MDH5MBPbIqV92AaeXatLxBI9gBamrtHrhAL1wy0L2yHvtyaeHbnfgDOvwBHrxAJfwnaebbnrfifHhDYfgasaacH8akY=wiFfYdH8Gipec8Eeeu0xXdbba9frFj0=OqFfea0dXdd9vqai=hGuQ8kuc9pgc9s8qqaq=dirpe0xb9q8qiLsFr0=vr0=vr0dc8meaabaqaciaacaGaaeqabaWaaeGaeaaakeaaimaacqWFZestaaa@3790@ is defined as πw(ℳ)=∑S∈SδS(ℳ)
 MathType@MTEF@5@5@+=feaafiart1ev1aaatCvAUfKttLearuWrP9MDH5MBPbIqV92AaeXatLxBI9gBamrtHrhAL1wy0L2yHvtyaeHbnfgDOvwBHrxAJfwnaebbnrfifHhDYfgasaacH8akY=wiFfYdH8Gipec8Eeeu0xXdbba9frFj0=OqFfea0dXdd9vqai=hGuQ8kuc9pgc9s8qqaq=dirpe0xb9q8qiLsFr0=vr0=vr0dc8meaabaqaciaacaGaaeqabaWaaeGaeaaakeaaiiGacqWFapaCdaWgaaWcbaacbiGae43DaChabeaakiabcIcaOGWaaiab9ntinjabcMcaPiabg2da9maaqababaGae8hTdq2aaSbaaSqaaiabdofatbqabaaabaGaem4uamLaeyicI4Sae0NeXpfabeqdcqGHris5aOGaeiikaGIae03mH0KaeiykaKcaaa@49FC@, i.e., total number of occurrences of ℳ
 MathType@MTEF@5@5@+=feaafiart1ev1aaatCvAUfKttLearuWrP9MDH5MBPbIqV92AaeXatLxBI9gBamrtHrhAL1wy0L2yHvtyaeHbnfgDOvwBHrxAJfwnaebbnrfifHhDYfgasaacH8akY=wiFfYdH8Gipec8Eeeu0xXdbba9frFj0=OqFfea0dXdd9vqai=hGuQ8kuc9pgc9s8qqaq=dirpe0xb9q8qiLsFr0=vr0=vr0dc8meaabaqaciaacaGaaeqabaWaaeGaeaaakeaaimaacqWFZestaaa@3790@ over all sequences in S
 MathType@MTEF@5@5@+=feaafiart1ev1aaatCvAUfKttLearuWrP9MDH5MBPbIqV92AaeXatLxBI9gBamrtHrhAL1wy0L2yHvtyaeHbnfgDOvwBHrxAJfwnaebbnrfifHhDYfgasaacH8akY=wiFfYdH8Gipec8Eeeu0xXdbba9frFj0=OqFfea0dXdd9vqai=hGuQ8kuc9pgc9s8qqaq=dirpe0xb9q8qiLsFr0=vr0=vr0dc8meaabaqaciaacaGaaeqabaWaaeGaeaaakeaaimaacqWFse=uaaa@3845@. We use O
 MathType@MTEF@5@5@+=feaafiart1ev1aaatCvAUfKttLearuWrP9MDH5MBPbIqV92AaeXatLxBI9gBamrtHrhAL1wy0L2yHvtyaeHbnfgDOvwBHrxAJfwnaebbnrfifHhDYfgasaacH8akY=wiFfYdH8Gipec8Eeeu0xXdbba9frFj0=OqFfea0dXdd9vqai=hGuQ8kuc9pgc9s8qqaq=dirpe0xb9q8qiLsFr0=vr0=vr0dc8meaabaqaciaacaGaaeqabaWaaeGaeaaakeaaimaacqWFoe=taaa@383D@ (ℳ
 MathType@MTEF@5@5@+=feaafiart1ev1aaatCvAUfKttLearuWrP9MDH5MBPbIqV92AaeXatLxBI9gBamrtHrhAL1wy0L2yHvtyaeHbnfgDOvwBHrxAJfwnaebbnrfifHhDYfgasaacH8akY=wiFfYdH8Gipec8Eeeu0xXdbba9frFj0=OqFfea0dXdd9vqai=hGuQ8kuc9pgc9s8qqaq=dirpe0xb9q8qiLsFr0=vr0=vr0dc8meaabaqaciaacaGaaeqabaWaaeGaeaaakeaaimaacqWFZestaaa@3790@) to denote the set of all occurrences of a structured motif ℳ
 MathType@MTEF@5@5@+=feaafiart1ev1aaatCvAUfKttLearuWrP9MDH5MBPbIqV92AaeXatLxBI9gBamrtHrhAL1wy0L2yHvtyaeHbnfgDOvwBHrxAJfwnaebbnrfifHhDYfgasaacH8akY=wiFfYdH8Gipec8Eeeu0xXdbba9frFj0=OqFfea0dXdd9vqai=hGuQ8kuc9pgc9s8qqaq=dirpe0xb9q8qiLsFr0=vr0=vr0dc8meaabaqaciaacaGaaeqabaWaaeGaeaaakeaaimaacqWFZestaaa@3790@. Given a user-specified quorum threshold *q *≥ 1, a motif that occurs at least *q *times will be called *frequent*.

There are two main tasks in the structured motif extraction problem: a) *Common Motifs *– find all motifs ℳ
 MathType@MTEF@5@5@+=feaafiart1ev1aaatCvAUfKttLearuWrP9MDH5MBPbIqV92AaeXatLxBI9gBamrtHrhAL1wy0L2yHvtyaeHbnfgDOvwBHrxAJfwnaebbnrfifHhDYfgasaacH8akY=wiFfYdH8Gipec8Eeeu0xXdbba9frFj0=OqFfea0dXdd9vqai=hGuQ8kuc9pgc9s8qqaq=dirpe0xb9q8qiLsFr0=vr0=vr0dc8meaabaqaciaacaGaaeqabaWaaeGaeaaakeaaimaacqWFZestaaa@3790@ in a set of sequences S
 MathType@MTEF@5@5@+=feaafiart1ev1aaatCvAUfKttLearuWrP9MDH5MBPbIqV92AaeXatLxBI9gBamrtHrhAL1wy0L2yHvtyaeHbnfgDOvwBHrxAJfwnaebbnrfifHhDYfgasaacH8akY=wiFfYdH8Gipec8Eeeu0xXdbba9frFj0=OqFfea0dXdd9vqai=hGuQ8kuc9pgc9s8qqaq=dirpe0xb9q8qiLsFr0=vr0=vr0dc8meaabaqaciaacaGaaeqabaWaaeGaeaaakeaaimaacqWFse=uaaa@3845@, such that the support of ℳ
 MathType@MTEF@5@5@+=feaafiart1ev1aaatCvAUfKttLearuWrP9MDH5MBPbIqV92AaeXatLxBI9gBamrtHrhAL1wy0L2yHvtyaeHbnfgDOvwBHrxAJfwnaebbnrfifHhDYfgasaacH8akY=wiFfYdH8Gipec8Eeeu0xXdbba9frFj0=OqFfea0dXdd9vqai=hGuQ8kuc9pgc9s8qqaq=dirpe0xb9q8qiLsFr0=vr0=vr0dc8meaabaqaciaacaGaaeqabaWaaeGaeaaakeaaimaacqWFZestaaa@3790@ is at least *q*, b) *Repeated Motifs *– find all motifs in a single sequence *S*, such that the weighted support of ℳ
 MathType@MTEF@5@5@+=feaafiart1ev1aaatCvAUfKttLearuWrP9MDH5MBPbIqV92AaeXatLxBI9gBamrtHrhAL1wy0L2yHvtyaeHbnfgDOvwBHrxAJfwnaebbnrfifHhDYfgasaacH8akY=wiFfYdH8Gipec8Eeeu0xXdbba9frFj0=OqFfea0dXdd9vqai=hGuQ8kuc9pgc9s8qqaq=dirpe0xb9q8qiLsFr0=vr0=vr0dc8meaabaqaciaacaGaaeqabaWaaeGaeaaakeaaimaacqWFZestaaa@3790@ is at least *q*. Furthermore, the structured motif extraction problem allows several variations:

• *Substitutions*: O
 MathType@MTEF@5@5@+=feaafiart1ev1aaatCvAUfKttLearuWrP9MDH5MBPbIqV92AaeXatLxBI9gBamrtHrhAL1wy0L2yHvtyaeHbnfgDOvwBHrxAJfwnaebbnrfifHhDYfgasaacH8akY=wiFfYdH8Gipec8Eeeu0xXdbba9frFj0=OqFfea0dXdd9vqai=hGuQ8kuc9pgc9s8qqaq=dirpe0xb9q8qiLsFr0=vr0=vr0dc8meaabaqaciaacaGaaeqabaWaaeGaeaaakeaaimaacqWFoe=taaa@383D@ may consist of similar motifs, as measured by *Hamming Distance *[3], instead of exact matches, to the simple motifs in ℳ
 MathType@MTEF@5@5@+=feaafiart1ev1aaatCvAUfKttLearuWrP9MDH5MBPbIqV92AaeXatLxBI9gBamrtHrhAL1wy0L2yHvtyaeHbnfgDOvwBHrxAJfwnaebbnrfifHhDYfgasaacH8akY=wiFfYdH8Gipec8Eeeu0xXdbba9frFj0=OqFfea0dXdd9vqai=hGuQ8kuc9pgc9s8qqaq=dirpe0xb9q8qiLsFr0=vr0=vr0dc8meaabaqaciaacaGaaeqabaWaaeGaeaaakeaaimaacqWFZestaaa@3790@. We can either allow for at most *ε*_*i *_errors for each simple motif *M*_*i*_, 1 ≤ *i *≤ *k*, or at most *ε *errors for the whole structured motif ℳ
 MathType@MTEF@5@5@+=feaafiart1ev1aaatCvAUfKttLearuWrP9MDH5MBPbIqV92AaeXatLxBI9gBamrtHrhAL1wy0L2yHvtyaeHbnfgDOvwBHrxAJfwnaebbnrfifHhDYfgasaacH8akY=wiFfYdH8Gipec8Eeeu0xXdbba9frFj0=OqFfea0dXdd9vqai=hGuQ8kuc9pgc9s8qqaq=dirpe0xb9q8qiLsFr0=vr0=vr0dc8meaabaqaciaacaGaaeqabaWaaeGaeaaakeaaimaacqWFZestaaa@3790@.

• *Overlapping Components*: The variable gap constraints (*l*_*i *_and *u*_*i*_) can take on a limited range of *negative *values, allowing search for overlapping simple motifs. We allow two adjacent components *M*_*i *_and *M*_*i*+1 _to overlap, but we require that *M*_*i*+1 _does not precede *M*_*i*_. This condition can be satisfied by the following constraints on the gap range [*l*_*i*_, *u*_*i*_]: -|*M*_*i*_| ≤ *l*_*i *_≤ *u*_*i*_, for *i *∈ [l, *k*). For example the search for motif template NNN[-2,2]NNN (where 'N' stands for any of the four DNA bases: A,C,G,T), may discover the pattern ACG[-2,2]CGA, representing an overlapped occurrence, ACGA, as well as a non-overlapped occurrence, ACG--CGA, at the two extremes of the gap range.

• *Motif Length Ranges*: Each simple motif *M*_*i *_in a template ℳ
 MathType@MTEF@5@5@+=feaafiart1ev1aaatCvAUfKttLearuWrP9MDH5MBPbIqV92AaeXatLxBI9gBamrtHrhAL1wy0L2yHvtyaeHbnfgDOvwBHrxAJfwnaebbnrfifHhDYfgasaacH8akY=wiFfYdH8Gipec8Eeeu0xXdbba9frFj0=OqFfea0dXdd9vqai=hGuQ8kuc9pgc9s8qqaq=dirpe0xb9q8qiLsFr0=vr0=vr0dc8meaabaqaciaacaGaaeqabaWaaeGaeaaakeaaimaacqWFZestaaa@3790@ can be of a range of lengths, i.e., |*M*_*i*_| ∈ [*l*_*a*_, *l*_*b*_], where *l*_*a *_and *l*_*b *_are the lower and upper bounds on the desired length.

Table 1 shows four example DNA sequences *S*_1_, *S*_2_, *S*_3_, *S*_4 _∈ S
 MathType@MTEF@5@5@+=feaafiart1ev1aaatCvAUfKttLearuWrP9MDH5MBPbIqV92AaeXatLxBI9gBamrtHrhAL1wy0L2yHvtyaeHbnfgDOvwBHrxAJfwnaebbnrfifHhDYfgasaacH8akY=wiFfYdH8Gipec8Eeeu0xXdbba9frFj0=OqFfea0dXdd9vqai=hGuQ8kuc9pgc9s8qqaq=dirpe0xb9q8qiLsFr0=vr0=vr0dc8meaabaqaciaacaGaaeqabaWaaeGaeaaakeaaimaacqWFse=uaaa@3845@; a structured motif template T
 MathType@MTEF@5@5@+=feaafiart1ev1aaatCvAUfKttLearuWrP9MDH5MBPbIqV92AaeXatLxBI9gBamrtHrhAL1wy0L2yHvtyaeHbnfgDOvwBHrxAJfwnaebbnrfifHhDYfgasaacH8akY=wiFfYdH8Gipec8Eeeu0xXdbba9frFj0=OqFfea0dXdd9vqai=hGuQ8kuc9pgc9s8qqaq=dirpe0xb9q8qiLsFr0=vr0=vr0dc8meaabaqaciaacaGaaeqabaWaaeGaeaaakeaaimaacqWFtepvaaa@3847@, where *M*_1 _= NNN, *M*_2 _= NN and *M*_3 _= NNNN, and [0,3] and [1,3] are the intervening gap ranges between the components; and a quorum threshold *q *= 2. The length of the template T
 MathType@MTEF@5@5@+=feaafiart1ev1aaatCvAUfKttLearuWrP9MDH5MBPbIqV92AaeXatLxBI9gBamrtHrhAL1wy0L2yHvtyaeHbnfgDOvwBHrxAJfwnaebbnrfifHhDYfgasaacH8akY=wiFfYdH8Gipec8Eeeu0xXdbba9frFj0=OqFfea0dXdd9vqai=hGuQ8kuc9pgc9s8qqaq=dirpe0xb9q8qiLsFr0=vr0=vr0dc8meaabaqaciaacaGaaeqabaWaaeGaeaaakeaaimaacqWFtepvaaa@3847@ is *k *= 3 and the number of symbols in ℳ
 MathType@MTEF@5@5@+=feaafiart1ev1aaatCvAUfKttLearuWrP9MDH5MBPbIqV92AaeXatLxBI9gBamrtHrhAL1wy0L2yHvtyaeHbnfgDOvwBHrxAJfwnaebbnrfifHhDYfgasaacH8akY=wiFfYdH8Gipec8Eeeu0xXdbba9frFj0=OqFfea0dXdd9vqai=hGuQ8kuc9pgc9s8qqaq=dirpe0xb9q8qiLsFr0=vr0=vr0dc8meaabaqaciaacaGaaeqabaWaaeGaeaaakeaaimaacqWFZestaaa@3790@ is *K *= 3 + 2 + 4 = 9. The span of gap ranges are: *W*_1 _= *u*_1 _- *l*_1 _+ 1 = 2 and *W*_2 _= *u*_2 _- *l*_2 _+ 1 = 2. If no substitutions are allowed, there are five frequent structured motifs in S
 MathType@MTEF@5@5@+=feaafiart1ev1aaatCvAUfKttLearuWrP9MDH5MBPbIqV92AaeXatLxBI9gBamrtHrhAL1wy0L2yHvtyaeHbnfgDOvwBHrxAJfwnaebbnrfifHhDYfgasaacH8akY=wiFfYdH8Gipec8Eeeu0xXdbba9frFj0=OqFfea0dXdd9vqai=hGuQ8kuc9pgc9s8qqaq=dirpe0xb9q8qiLsFr0=vr0=vr0dc8meaabaqaciaacaGaaeqabaWaaeGaeaaakeaaimaacqWFse=uaaa@3845@ matching the template T
 MathType@MTEF@5@5@+=feaafiart1ev1aaatCvAUfKttLearuWrP9MDH5MBPbIqV92AaeXatLxBI9gBamrtHrhAL1wy0L2yHvtyaeHbnfgDOvwBHrxAJfwnaebbnrfifHhDYfgasaacH8akY=wiFfYdH8Gipec8Eeeu0xXdbba9frFj0=OqFfea0dXdd9vqai=hGuQ8kuc9pgc9s8qqaq=dirpe0xb9q8qiLsFr0=vr0=vr0dc8meaabaqaciaacaGaaeqabaWaaeGaeaaakeaaimaacqWFtepvaaa@3847@, namely ℳ
 MathType@MTEF@5@5@+=feaafiart1ev1aaatCvAUfKttLearuWrP9MDH5MBPbIqV92AaeXatLxBI9gBamrtHrhAL1wy0L2yHvtyaeHbnfgDOvwBHrxAJfwnaebbnrfifHhDYfgasaacH8akY=wiFfYdH8Gipec8Eeeu0xXdbba9frFj0=OqFfea0dXdd9vqai=hGuQ8kuc9pgc9s8qqaq=dirpe0xb9q8qiLsFr0=vr0=vr0dc8meaabaqaciaacaGaaeqabaWaaeGaeaaakeaaimaacqWFZestaaa@3790@_1 _= CCG[0,3]TA[1,3]GAAC (shown in bold) and ℳ
 MathType@MTEF@5@5@+=feaafiart1ev1aaatCvAUfKttLearuWrP9MDH5MBPbIqV92AaeXatLxBI9gBamrtHrhAL1wy0L2yHvtyaeHbnfgDOvwBHrxAJfwnaebbnrfifHhDYfgasaacH8akY=wiFfYdH8Gipec8Eeeu0xXdbba9frFj0=OqFfea0dXdd9vqai=hGuQ8kuc9pgc9s8qqaq=dirpe0xb9q8qiLsFr0=vr0=vr0dc8meaabaqaciaacaGaaeqabaWaaeGaeaaakeaaimaacqWFZestaaa@3790@_2 _= CCG[0,3]TA[1,3]AACC which occur in *S*_1 _and *S*_2_; ℳ
 MathType@MTEF@5@5@+=feaafiart1ev1aaatCvAUfKttLearuWrP9MDH5MBPbIqV92AaeXatLxBI9gBamrtHrhAL1wy0L2yHvtyaeHbnfgDOvwBHrxAJfwnaebbnrfifHhDYfgasaacH8akY=wiFfYdH8Gipec8Eeeu0xXdbba9frFj0=OqFfea0dXdd9vqai=hGuQ8kuc9pgc9s8qqaq=dirpe0xb9q8qiLsFr0=vr0=vr0dc8meaabaqaciaacaGaaeqabaWaaeGaeaaakeaaimaacqWFZestaaa@3790@_3 _= TAT[0,3]GG[1,3]ACCA (shown underlined), ℳ
 MathType@MTEF@5@5@+=feaafiart1ev1aaatCvAUfKttLearuWrP9MDH5MBPbIqV92AaeXatLxBI9gBamrtHrhAL1wy0L2yHvtyaeHbnfgDOvwBHrxAJfwnaebbnrfifHhDYfgasaacH8akY=wiFfYdH8Gipec8Eeeu0xXdbba9frFj0=OqFfea0dXdd9vqai=hGuQ8kuc9pgc9s8qqaq=dirpe0xb9q8qiLsFr0=vr0=vr0dc8meaabaqaciaacaGaaeqabaWaaeGaeaaakeaaimaacqWFZestaaa@3790@_4 _= TAT[0,3]GA[1,3]CCAT and ℳ
 MathType@MTEF@5@5@+=feaafiart1ev1aaatCvAUfKttLearuWrP9MDH5MBPbIqV92AaeXatLxBI9gBamrtHrhAL1wy0L2yHvtyaeHbnfgDOvwBHrxAJfwnaebbnrfifHhDYfgasaacH8akY=wiFfYdH8Gipec8Eeeu0xXdbba9frFj0=OqFfea0dXdd9vqai=hGuQ8kuc9pgc9s8qqaq=dirpe0xb9q8qiLsFr0=vr0=vr0dc8meaabaqaciaacaGaaeqabaWaaeGaeaaakeaaimaacqWFZestaaa@3790@_5 _= TAT[0,3] GG[1,3]CCAT which occur in *S*_2 _and *S*_3_. If substitutions are allows, say, *e*_1 _= 1 = *e*_3_, then the occurrence of ℳ
 MathType@MTEF@5@5@+=feaafiart1ev1aaatCvAUfKttLearuWrP9MDH5MBPbIqV92AaeXatLxBI9gBamrtHrhAL1wy0L2yHvtyaeHbnfgDOvwBHrxAJfwnaebbnrfifHhDYfgasaacH8akY=wiFfYdH8Gipec8Eeeu0xXdbba9frFj0=OqFfea0dXdd9vqai=hGuQ8kuc9pgc9s8qqaq=dirpe0xb9q8qiLsFr0=vr0=vr0dc8meaabaqaciaacaGaaeqabaWaaeGaeaaakeaaimaacqWFZestaaa@3790@_6 _= TAA[0,3]GG[1,3] CCCT (shown underlined) in *S*_4 _will be considered to match motif ℳ
 MathType@MTEF@5@5@+=feaafiart1ev1aaatCvAUfKttLearuWrP9MDH5MBPbIqV92AaeXatLxBI9gBamrtHrhAL1wy0L2yHvtyaeHbnfgDOvwBHrxAJfwnaebbnrfifHhDYfgasaacH8akY=wiFfYdH8Gipec8Eeeu0xXdbba9frFj0=OqFfea0dXdd9vqai=hGuQ8kuc9pgc9s8qqaq=dirpe0xb9q8qiLsFr0=vr0=vr0dc8meaabaqaciaacaGaaeqabaWaaeGaeaaakeaaimaacqWFZestaaa@3790@_5_.

**Table 1:** Structured motif extraction.

Sequence *S*_1 _(∈ S MathType@MTEF@5@5@+=feaafiart1ev1aaatCvAUfKttLearuWrP9MDH5MBPbIqV92AaeXatLxBI9gBamrtHrhAL1wy0L2yHvtyaeHbnfgDOvwBHrxAJfwnaebbnrfifHhDYfgasaacH8akY=wiFfYdH8Gipec8Eeeu0xXdbba9frFj0=OqFfea0dXdd9vqai=hGuQ8kuc9pgc9s8qqaq=dirpe0xb9q8qiLsFr0=vr0=vr0dc8meaabaqaciaacaGaaeqabaWaaeGaeaaakeaaimaacqWFse=uaaa@3845@):	**CCGTA**CC**GAA**CCTCAAA
Sequence *S*_2 _(∈ S MathType@MTEF@5@5@+=feaafiart1ev1aaatCvAUfKttLearuWrP9MDH5MBPbIqV92AaeXatLxBI9gBamrtHrhAL1wy0L2yHvtyaeHbnfgDOvwBHrxAJfwnaebbnrfifHhDYfgasaacH8akY=wiFfYdH8Gipec8Eeeu0xXdbba9frFj0=OqFfea0dXdd9vqai=hGuQ8kuc9pgc9s8qqaq=dirpe0xb9q8qiLsFr0=vr0=vr0dc8meaabaqaciaacaGaaeqabaWaaeGaeaaakeaaimaacqWFse=uaaa@3845@):	**CCG**T**TATA**G**GAAC**CATT
Sequence *S*_3 _(∈ S MathType@MTEF@5@5@+=feaafiart1ev1aaatCvAUfKttLearuWrP9MDH5MBPbIqV92AaeXatLxBI9gBamrtHrhAL1wy0L2yHvtyaeHbnfgDOvwBHrxAJfwnaebbnrfifHhDYfgasaacH8akY=wiFfYdH8Gipec8Eeeu0xXdbba9frFj0=OqFfea0dXdd9vqai=hGuQ8kuc9pgc9s8qqaq=dirpe0xb9q8qiLsFr0=vr0=vr0dc8meaabaqaciaacaGaaeqabaWaaeGaeaaakeaaimaacqWFse=uaaa@3845@):	TATGGAACCATCTT
Sequence *S*_4 _(∈ S MathType@MTEF@5@5@+=feaafiart1ev1aaatCvAUfKttLearuWrP9MDH5MBPbIqV92AaeXatLxBI9gBamrtHrhAL1wy0L2yHvtyaeHbnfgDOvwBHrxAJfwnaebbnrfifHhDYfgasaacH8akY=wiFfYdH8Gipec8Eeeu0xXdbba9frFj0=OqFfea0dXdd9vqai=hGuQ8kuc9pgc9s8qqaq=dirpe0xb9q8qiLsFr0=vr0=vr0dc8meaabaqaciaacaGaaeqabaWaaeGaeaaakeaaimaacqWFse=uaaa@3845@):	TAACGGATCCCTTT
Structured Motif Template (T MathType@MTEF@5@5@+=feaafiart1ev1aaatCvAUfKttLearuWrP9MDH5MBPbIqV92AaeXatLxBI9gBamrtHrhAL1wy0L2yHvtyaeHbnfgDOvwBHrxAJfwnaebbnrfifHhDYfgasaacH8akY=wiFfYdH8Gipec8Eeeu0xXdbba9frFj0=OqFfea0dXdd9vqai=hGuQ8kuc9pgc9s8qqaq=dirpe0xb9q8qiLsFr0=vr0=vr0dc8meaabaqaciaacaGaaeqabaWaaeGaeaaakeaaimaacqWFtepvaaa@3847@):	NNN[0,3]NN[1,3]NNNN
Quorum (*q*):	2

In this paper, we propose EXMOTIF, an efficient algorithm for both the structured motif extraction problems. It uses an inverted index of symbol positions, and it enumerates all structured motifs by *positional joins *over this index. The variable gap constraints are also considered at the same time as the joins, resulting in considerable efficiency. In order to save time and space, we only keep the start positions of each intermediate pattern during the positional join.

## Related work

Many simple motif extraction algorithms have been proposed primarily for extracting the transcription factor binding sites, where each motif consists of a unique binding site [4-10] or two binding sites separated by a fixed number of gaps [11-13]. A pattern with a single component is also called a *monad pattern*. Structured motif extraction problems, in which variable number of gaps are allowed, have attracted much attention recently, where the structured motifs can be extracted either from multiple sequences [14-21] or from a single sequence [22,23]. In many cases, more than one transcription factor may cooperatively regulate a gene. Such patterns are called *composite regulatory patterns*. To detect the composite regulatory patterns, one may apply single binding site identification algorithms to detect each component separately. However, this solution may fail when some components are not very strong (significant). Thus it is necessary to detect the whole composite regulatory patterns (even with weak components) directly, whose gaps and other possibly strong components can increase its significance.

Several algorithms have been used to address the composite pattern discovery with two components, which are called *dyad patterns*. Helden et al. [11] propose a method for dyad analysis, which exhaustively counts the number of occurrences of each possible pair of patterns in the sequences and then assesses their statistical significance. This method can only deal with fixed number of gaps between the two components. MITRA [12] first casts the composite pattern discovery problem as a larger monad discovery problem and then applies an exhaustive monad discovery algorithm. It can handle several mismatches but can only handle sequences less than 60 kilo-bases long. Co-Bind [24] models composite transcription factors with Position Weight Matrices (PWMs) and finds PWMs that maximize the joint likelihood of occurrences of the two binding site components. Co-Bind uses Gibbs sampling to select binding sites and then refines the PWMs for a fixed number of times. Co-Bind may miss some binding sites since not all patterns in the sequences are considered. Moreover, using a fixed number of iterations for improvement may not converge to the global optimal dyad PWM.

SMILE [14] describes four variants of increasing generality for common structured motif extraction, and proposes two solutions for them. The two approaches for the first problem, in which the structured motif template consists of two components with a gap range between them, both start by building a generalized suffix tree for the input sequences and extracting the first component. Then in the first approach, the second component is extracted by simply jumping in the sequences from the end of the first one to the second within the gap range. In the second approach, the suffix tree is temporarily modified so as to extract the second component from the modified suffix tree directly. The drawback of SMILE is that its time and space complexity are exponential in the number of gaps between the two components. In order to reduce the time during the extraction of the structured motifs, [18] presents a parallel algorithm, PSmile, based on SMILE, where the search space is well-partitioned among the available processors.

RISO [15-17] improves SMILE in two aspects. First, instead of building the whole suffix tree for the input sequences, RISO builds a suffix tree only up to a certain level *l*, called a *factor tree*, which leads to a large space saving. Second, a new data structure called *box-link *is proposed to store the information about how to jump within the DNA sequences from one simple component (box) to the subsequent one in the structured motif. This accelerates the extraction process and avoids exponential time and space consumption (in the gaps) as in SMILE. In RISO, after the generalized factor tree is built, the box-links are constructed by exhaustively enumerating all the possible structured motifs in the sequences and are added to the leaves of the factor tree. Then the extraction process begins during which the factor tree may be temporarily and partially modified so as to extract the subsequent simple motifs. Since during the box-link construction, the structured motif occurrences are exhaustively enumerated and the frequency threshold is never used to prune the candidate structured motifs, RISO needs a lot of computation during this step.

For repeated structured motif identification problem, the frequency closure property that "all the subsequences of a frequent sequence must be frequent", doesn't hold any more since the frequency of a pattern can exceed the frequency of its sub-patterns. [22] introduces an closure-like property which can help prune the patterns without missing the frequent patterns. The two algorithms proposed in [22] can extract within one sequence all frequent patterns of length no greater than a length threshold, which can be either manually specified or automatically determined. However, this method requires that all the gap ranges [*l*_*i*_, *u*_*i*_], between adjacent *symbols *in the structured motif be the same, i.e., [*l*_*i*_, *u*_*i*_] = [*l*, *u*] for all *i *∈ [1, *k *- 1]. Moreover, approximate matches are not allowed for the structured motif.

## The EXMOTIF algorithm

We first introduce our basic approach for common structured motif extraction problem. We then successively optimize it for various practical scenarios.

### The basic approach

Let's assume that we are extracting all structured motif instances from *n *sequence S
 MathType@MTEF@5@5@+=feaafiart1ev1aaatCvAUfKttLearuWrP9MDH5MBPbIqV92AaeXatLxBI9gBamrtHrhAL1wy0L2yHvtyaeHbnfgDOvwBHrxAJfwnaebbnrfifHhDYfgasaacH8akY=wiFfYdH8Gipec8Eeeu0xXdbba9frFj0=OqFfea0dXdd9vqai=hGuQ8kuc9pgc9s8qqaq=dirpe0xb9q8qiLsFr0=vr0=vr0dc8meaabaqaciaacaGaaeqabaWaaeGaeaaakeaaimaacqWFse=uaaa@3845@ = {*S*_*i*_, 1 ≤ *i *≤ *n*}, each of which satisfies the template T
 MathType@MTEF@5@5@+=feaafiart1ev1aaatCvAUfKttLearuWrP9MDH5MBPbIqV92AaeXatLxBI9gBamrtHrhAL1wy0L2yHvtyaeHbnfgDOvwBHrxAJfwnaebbnrfifHhDYfgasaacH8akY=wiFfYdH8Gipec8Eeeu0xXdbba9frFj0=OqFfea0dXdd9vqai=hGuQ8kuc9pgc9s8qqaq=dirpe0xb9q8qiLsFr0=vr0=vr0dc8meaabaqaciaacaGaaeqabaWaaeGaeaaakeaaimaacqWFtepvaaa@3847@ and occurs at least in *q *sequences of S
 MathType@MTEF@5@5@+=feaafiart1ev1aaatCvAUfKttLearuWrP9MDH5MBPbIqV92AaeXatLxBI9gBamrtHrhAL1wy0L2yHvtyaeHbnfgDOvwBHrxAJfwnaebbnrfifHhDYfgasaacH8akY=wiFfYdH8Gipec8Eeeu0xXdbba9frFj0=OqFfea0dXdd9vqai=hGuQ8kuc9pgc9s8qqaq=dirpe0xb9q8qiLsFr0=vr0=vr0dc8meaabaqaciaacaGaaeqabaWaaeGaeaaakeaaimaacqWFse=uaaa@3845@. We assume for the moment that no substitutions are allowed in any of the simple motifs. We also assume that all *S*_*i *_∈ S
 MathType@MTEF@5@5@+=feaafiart1ev1aaatCvAUfKttLearuWrP9MDH5MBPbIqV92AaeXatLxBI9gBamrtHrhAL1wy0L2yHvtyaeHbnfgDOvwBHrxAJfwnaebbnrfifHhDYfgasaacH8akY=wiFfYdH8Gipec8Eeeu0xXdbba9frFj0=OqFfea0dXdd9vqai=hGuQ8kuc9pgc9s8qqaq=dirpe0xb9q8qiLsFr0=vr0=vr0dc8meaabaqaciaacaGaaeqabaWaaeGaeaaakeaaimaacqWFse=uaaa@3845@, 1 ≤ *i *≤ *n *and the extracted motifs are over the DNA alphabet, Σ_DNA_. EXMOTIF first converts each *S*_*i *_∈ S
 MathType@MTEF@5@5@+=feaafiart1ev1aaatCvAUfKttLearuWrP9MDH5MBPbIqV92AaeXatLxBI9gBamrtHrhAL1wy0L2yHvtyaeHbnfgDOvwBHrxAJfwnaebbnrfifHhDYfgasaacH8akY=wiFfYdH8Gipec8Eeeu0xXdbba9frFj0=OqFfea0dXdd9vqai=hGuQ8kuc9pgc9s8qqaq=dirpe0xb9q8qiLsFr0=vr0=vr0dc8meaabaqaciaacaGaaeqabaWaaeGaeaaakeaaimaacqWFse=uaaa@3845@, 1 ≤ *i *≤ *n *into an equivalent inverted format [25], where we associate with each symbol in the sequence *S*_*i *_its *pos-list*, a *sorted *list of the positions where the symbol occurs in *S*_*i*_. Then for each symbol we combine its pos-list in each *S*_*i *_to obtain its pos-list in S
 MathType@MTEF@5@5@+=feaafiart1ev1aaatCvAUfKttLearuWrP9MDH5MBPbIqV92AaeXatLxBI9gBamrtHrhAL1wy0L2yHvtyaeHbnfgDOvwBHrxAJfwnaebbnrfifHhDYfgasaacH8akY=wiFfYdH8Gipec8Eeeu0xXdbba9frFj0=OqFfea0dXdd9vqai=hGuQ8kuc9pgc9s8qqaq=dirpe0xb9q8qiLsFr0=vr0=vr0dc8meaabaqaciaacaGaaeqabaWaaeGaeaaakeaaimaacqWFse=uaaa@3845@. More formally, for a symbol *X *∈ Σ_DNA_, its pos-list in *S*_*i *_is given as P (*X*, *S*_*i*_) = {*j *| *S*_*i*_[*j*] = *X*, *j *∈ [1, |*S*_*i*_|]}, where *S*_*i*_[*j*] is the symbol at position *j *in *S*_*i*_, and |*S*_*i*_| denotes the length of *S*_*i*_. Its pos-list across all sequences S
 MathType@MTEF@5@5@+=feaafiart1ev1aaatCvAUfKttLearuWrP9MDH5MBPbIqV92AaeXatLxBI9gBamrtHrhAL1wy0L2yHvtyaeHbnfgDOvwBHrxAJfwnaebbnrfifHhDYfgasaacH8akY=wiFfYdH8Gipec8Eeeu0xXdbba9frFj0=OqFfea0dXdd9vqai=hGuQ8kuc9pgc9s8qqaq=dirpe0xb9q8qiLsFr0=vr0=vr0dc8meaabaqaciaacaGaaeqabaWaaeGaeaaakeaaimaacqWFse=uaaa@3845@ is obtained by grouping the pos-lists of each sequence, and is given as P (*X*, S
 MathType@MTEF@5@5@+=feaafiart1ev1aaatCvAUfKttLearuWrP9MDH5MBPbIqV92AaeXatLxBI9gBamrtHrhAL1wy0L2yHvtyaeHbnfgDOvwBHrxAJfwnaebbnrfifHhDYfgasaacH8akY=wiFfYdH8Gipec8Eeeu0xXdbba9frFj0=OqFfea0dXdd9vqai=hGuQ8kuc9pgc9s8qqaq=dirpe0xb9q8qiLsFr0=vr0=vr0dc8meaabaqaciaacaGaaeqabaWaaeGaeaaakeaaimaacqWFse=uaaa@3845@) = {⟨ *i*, | P (*X*, *S*_*i*_)|, P (*X*, *S*_*i*_)⟩ | *S*_*i *_∈ S
 MathType@MTEF@5@5@+=feaafiart1ev1aaatCvAUfKttLearuWrP9MDH5MBPbIqV92AaeXatLxBI9gBamrtHrhAL1wy0L2yHvtyaeHbnfgDOvwBHrxAJfwnaebbnrfifHhDYfgasaacH8akY=wiFfYdH8Gipec8Eeeu0xXdbba9frFj0=OqFfea0dXdd9vqai=hGuQ8kuc9pgc9s8qqaq=dirpe0xb9q8qiLsFr0=vr0=vr0dc8meaabaqaciaacaGaaeqabaWaaeGaeaaakeaaimaacqWFse=uaaa@3845@}, where *i *is the *sequence identifier *of *S*_*i*_, and | P (*X*, *S*_*i*_)| denotes the cardinality of the pos-list P (*X*, *S*_*i*_) in sequence *S*_*i*_. For our example sequences in Table 1, the pos-list for each DNA base is given in Table 2. For example, A occurs in sequence *S*_1 _at the positions {5, 9, 10, 15, 16, 17}, thus the entries in A's pos-list are {**1**, **6**, 5, 9, 10, 15, 16, 17}.

**Table 2:** Pos-lists.

X	*pos-lists*
A	{**1**,**6**,5,9,10,15,16,17, **2**,**5**,6,8,11,12,15, **3**,**4**,2,6,7,10, **4**,**3**,2,3,7}
C	{**1**,**7**,1,2,6,7,11,12,14, **2**,**4**,1,2,13,14, **3**,**3**,8,9,12, **4**,**4**,4,9,10,11}
G	{**1**,**2**,3,8, **2**,**3**,3,9,10, **3**,**2**,4,5, **4**, **2**,5,6}
T	{**1**,**2**,4,13, **2**,**5**,4,5,7,16,17 **3**,**5**,1,3,11,13,14, **4**,**5**,1,8,12,13,14}

Sequence identifiers (*i*) and cardinality of P (*X*, *S*_*i*_) are marked in bold.

#### Positional joins

We first extend the notion of pos-lists to cover structured motifs. The pos-list of ℳ
 MathType@MTEF@5@5@+=feaafiart1ev1aaatCvAUfKttLearuWrP9MDH5MBPbIqV92AaeXatLxBI9gBamrtHrhAL1wy0L2yHvtyaeHbnfgDOvwBHrxAJfwnaebbnrfifHhDYfgasaacH8akY=wiFfYdH8Gipec8Eeeu0xXdbba9frFj0=OqFfea0dXdd9vqai=hGuQ8kuc9pgc9s8qqaq=dirpe0xb9q8qiLsFr0=vr0=vr0dc8meaabaqaciaacaGaaeqabaWaaeGaeaaakeaaimaacqWFZestaaa@3790@ in *S*_*i *_∈ S
 MathType@MTEF@5@5@+=feaafiart1ev1aaatCvAUfKttLearuWrP9MDH5MBPbIqV92AaeXatLxBI9gBamrtHrhAL1wy0L2yHvtyaeHbnfgDOvwBHrxAJfwnaebbnrfifHhDYfgasaacH8akY=wiFfYdH8Gipec8Eeeu0xXdbba9frFj0=OqFfea0dXdd9vqai=hGuQ8kuc9pgc9s8qqaq=dirpe0xb9q8qiLsFr0=vr0=vr0dc8meaabaqaciaacaGaaeqabaWaaeGaeaaakeaaimaacqWFse=uaaa@3845@ is given as the set of start positions of all the matches of ℳ
 MathType@MTEF@5@5@+=feaafiart1ev1aaatCvAUfKttLearuWrP9MDH5MBPbIqV92AaeXatLxBI9gBamrtHrhAL1wy0L2yHvtyaeHbnfgDOvwBHrxAJfwnaebbnrfifHhDYfgasaacH8akY=wiFfYdH8Gipec8Eeeu0xXdbba9frFj0=OqFfea0dXdd9vqai=hGuQ8kuc9pgc9s8qqaq=dirpe0xb9q8qiLsFr0=vr0=vr0dc8meaabaqaciaacaGaaeqabaWaaeGaeaaakeaaimaacqWFZestaaa@3790@ in *S*_*i*_. Let *X*, *Y *∈ Σ_DNA _be any two symbols, and let ℳ
 MathType@MTEF@5@5@+=feaafiart1ev1aaatCvAUfKttLearuWrP9MDH5MBPbIqV92AaeXatLxBI9gBamrtHrhAL1wy0L2yHvtyaeHbnfgDOvwBHrxAJfwnaebbnrfifHhDYfgasaacH8akY=wiFfYdH8Gipec8Eeeu0xXdbba9frFj0=OqFfea0dXdd9vqai=hGuQ8kuc9pgc9s8qqaq=dirpe0xb9q8qiLsFr0=vr0=vr0dc8meaabaqaciaacaGaaeqabaWaaeGaeaaakeaaimaacqWFZestaaa@3790@ = *X*[*l*, *u*]*Y *be a structured motif. Given the pos-lists of *X *and *Y *in *S*_*i *_for 1 ≤ *i *≤ *n*, namely, P (*X*, *S*_*i*_) and P (*Y*, *S*_*i*_), the pos-list of ℳ
 MathType@MTEF@5@5@+=feaafiart1ev1aaatCvAUfKttLearuWrP9MDH5MBPbIqV92AaeXatLxBI9gBamrtHrhAL1wy0L2yHvtyaeHbnfgDOvwBHrxAJfwnaebbnrfifHhDYfgasaacH8akY=wiFfYdH8Gipec8Eeeu0xXdbba9frFj0=OqFfea0dXdd9vqai=hGuQ8kuc9pgc9s8qqaq=dirpe0xb9q8qiLsFr0=vr0=vr0dc8meaabaqaciaacaGaaeqabaWaaeGaeaaakeaaimaacqWFZestaaa@3790@ in *S*_*i *_can be obtained by a positional join as follows: for a position *x *∈ P (*X*, *S*_*i*_), if there exists a position *y *∈ P (*Y*, *S*_*i*_), such that *l *≤ *y *- *x *- 1 ≤ *u*, it means that *Y *follows *X *within the variable gap range [*l*, *u*] in the sequence *S*_*i*_, and thus we can add *x *to the pos-list of motif *X*[*l*, *u*]*Y*. Let *d *be the number of gaps between *x *∈ P (*X*, *S*_*i*_) and *y *∈ P (*Y*, *S*_*i*_), given as *d *= *y *- *x *- 1.

Then, in general, there are three cases to consider in the positional join algorithm:

• *d *<*l*: Advance *y *to the next element in P (*Y*, *S*_*i*_).

• *d *> *u*: Advance *x *to the next element in P (*X*, *S*_*i*_).

• *l *≤ *d *≤ *u*: Save this occurrence in P (*X*[*l*, *u*]*Y*, *S*_*i*_), and then advance *x *to the next element in P (*X*, *S*_*i*_).

**Figure 1:**
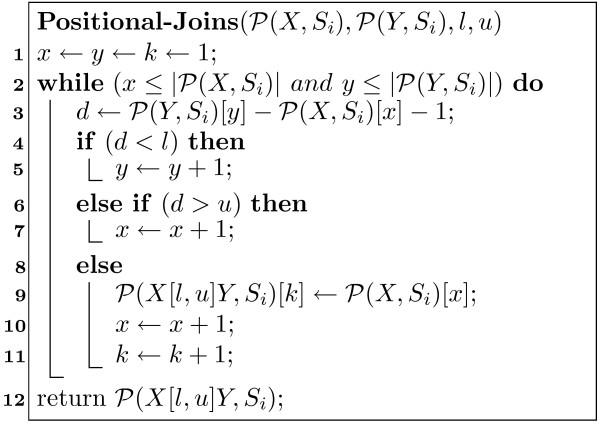
Positional Joins Algorithm.

The pos-list for *X*[*l*, *u*]*Y *can be computed in time linear in the lengths of P (*X*, *S*_*i*_) and P (*Y*, *S*_*i*_), i.e., the complexity of a positional join is *O*(|P (*X*, *S*_*i*_)| + |P (*Y*, *S*_*i*_)|). In essence, each time we advance *x *∈ P (*X*, *S*_*i*_), we check if there exists a *y *∈ P (*Y*, *S*_*i*_) that satisfies the given gap constraint. Instead of searching for the matching *y *from the beginning of the pos-list each time, we search from the last position used to compare with *x*. This results in fast positional joins. For example, during the positional join for the motif A[0,1]T in *S*_4_, with *l *= 0 and *u *= 1, we scan the pos-lists of A and T for *S*_4 _in Table 2, i.e. P (*X*, *S*_4_) = {2, 3, 7} and P (*Y*, *S*_4_) = {1, 8, 12, 13, 14}. Initially, *x *= 2 and *y *= 1. This gives *d *= 1 - 2 - 1 = - 2 <*l*, thus we advance *y *to 8. Next, *d *= 8 - 2 - 1 = 5 > *u*, thus we advance *x *to 3.  Then, *d *= 8 - 3 - 1 = 4 > *u*, thus we advance *x *to 7. Next, *d *= 8 - 7 - 1 = 0 ∈ [*l*, *u*], so we store *x *= 7 in P (*A*[0, 1]*T*, *S*_4_). We would advance *x *but since we have already reached the end of P (*A*, *S*_4_), the positional join stops. Thus the final pos-list of A[0,1]T in *S*_4 _is: P (*A*[0, 1]*T*, *S*_4_) = {7}. After we obtain the pos-list of ℳ
 MathType@MTEF@5@5@+=feaafiart1ev1aaatCvAUfKttLearuWrP9MDH5MBPbIqV92AaeXatLxBI9gBamrtHrhAL1wy0L2yHvtyaeHbnfgDOvwBHrxAJfwnaebbnrfifHhDYfgasaacH8akY=wiFfYdH8Gipec8Eeeu0xXdbba9frFj0=OqFfea0dXdd9vqai=hGuQ8kuc9pgc9s8qqaq=dirpe0xb9q8qiLsFr0=vr0=vr0dc8meaabaqaciaacaGaaeqabaWaaeGaeaaakeaaimaacqWFZestaaa@3790@ in each *S*_*i *_for 1 ≤ *i *≤ *n*, we can combine them together to obtain the pos-list of ℳ
 MathType@MTEF@5@5@+=feaafiart1ev1aaatCvAUfKttLearuWrP9MDH5MBPbIqV92AaeXatLxBI9gBamrtHrhAL1wy0L2yHvtyaeHbnfgDOvwBHrxAJfwnaebbnrfifHhDYfgasaacH8akY=wiFfYdH8Gipec8Eeeu0xXdbba9frFj0=OqFfea0dXdd9vqai=hGuQ8kuc9pgc9s8qqaq=dirpe0xb9q8qiLsFr0=vr0=vr0dc8meaabaqaciaacaGaaeqabaWaaeGaeaaakeaaimaacqWFZestaaa@3790@ in S
 MathType@MTEF@5@5@+=feaafiart1ev1aaatCvAUfKttLearuWrP9MDH5MBPbIqV92AaeXatLxBI9gBamrtHrhAL1wy0L2yHvtyaeHbnfgDOvwBHrxAJfwnaebbnrfifHhDYfgasaacH8akY=wiFfYdH8Gipec8Eeeu0xXdbba9frFj0=OqFfea0dXdd9vqai=hGuQ8kuc9pgc9s8qqaq=dirpe0xb9q8qiLsFr0=vr0=vr0dc8meaabaqaciaacaGaaeqabaWaaeGaeaaakeaaimaacqWFse=uaaa@3845@. For example, the full pos-list of A[0,1]T for S
 MathType@MTEF@5@5@+=feaafiart1ev1aaatCvAUfKttLearuWrP9MDH5MBPbIqV92AaeXatLxBI9gBamrtHrhAL1wy0L2yHvtyaeHbnfgDOvwBHrxAJfwnaebbnrfifHhDYfgasaacH8akY=wiFfYdH8Gipec8Eeeu0xXdbba9frFj0=OqFfea0dXdd9vqai=hGuQ8kuc9pgc9s8qqaq=dirpe0xb9q8qiLsFr0=vr0=vr0dc8meaabaqaciaacaGaaeqabaWaaeGaeaaakeaaimaacqWFse=uaaa@3845@ is: {**2**, **2**, 6, 15, **3**, **2**, 2, 10, **4, 1**, 7}. Thus the support of A[0,1]T is 3. Note here for each non-empty pos-list, we insert its sequence identifier and length before it. The pseudo-code for the positional joins for a given sequence *S*_*i *_∈ S
 MathType@MTEF@5@5@+=feaafiart1ev1aaatCvAUfKttLearuWrP9MDH5MBPbIqV92AaeXatLxBI9gBamrtHrhAL1wy0L2yHvtyaeHbnfgDOvwBHrxAJfwnaebbnrfifHhDYfgasaacH8akY=wiFfYdH8Gipec8Eeeu0xXdbba9frFj0=OqFfea0dXdd9vqai=hGuQ8kuc9pgc9s8qqaq=dirpe0xb9q8qiLsFr0=vr0=vr0dc8meaabaqaciaacaGaaeqabaWaaeGaeaaakeaaimaacqWFse=uaaa@3845@ is shown in Figure 1. The full pos-list is obtained by concatenating the pos-lists from each sequence *S*_*i*_.

Given a longer motif ℳ
 MathType@MTEF@5@5@+=feaafiart1ev1aaatCvAUfKttLearuWrP9MDH5MBPbIqV92AaeXatLxBI9gBamrtHrhAL1wy0L2yHvtyaeHbnfgDOvwBHrxAJfwnaebbnrfifHhDYfgasaacH8akY=wiFfYdH8Gipec8Eeeu0xXdbba9frFj0=OqFfea0dXdd9vqai=hGuQ8kuc9pgc9s8qqaq=dirpe0xb9q8qiLsFr0=vr0=vr0dc8meaabaqaciaacaGaaeqabaWaaeGaeaaakeaaimaacqWFZestaaa@3790@, the positional joins start with the last two symbols, and proceed by successively joining the pos-list of the current symbol with the intermediate pos-list of the suffix. That is, the intermediate pos-list for a *(l+1)*-length pattern (with *l *≥ 1) is obtained by doing a positional join of the pos-list of the pattern's first symbol, called the *head symbol*, with the pos-list of its *l*-length suffix, called the *tail*. As the computation progresses the previous tail pos-lists are discarded. Combined with the fact that only start positions are kept in a pos-list, this saves both time and space.

In order to enumerate all frequent motifs instances ℳ
 MathType@MTEF@5@5@+=feaafiart1ev1aaatCvAUfKttLearuWrP9MDH5MBPbIqV92AaeXatLxBI9gBamrtHrhAL1wy0L2yHvtyaeHbnfgDOvwBHrxAJfwnaebbnrfifHhDYfgasaacH8akY=wiFfYdH8Gipec8Eeeu0xXdbba9frFj0=OqFfea0dXdd9vqai=hGuQ8kuc9pgc9s8qqaq=dirpe0xb9q8qiLsFr0=vr0=vr0dc8meaabaqaciaacaGaaeqabaWaaeGaeaaakeaaimaacqWFZestaaa@3790@ in S
 MathType@MTEF@5@5@+=feaafiart1ev1aaatCvAUfKttLearuWrP9MDH5MBPbIqV92AaeXatLxBI9gBamrtHrhAL1wy0L2yHvtyaeHbnfgDOvwBHrxAJfwnaebbnrfifHhDYfgasaacH8akY=wiFfYdH8Gipec8Eeeu0xXdbba9frFj0=OqFfea0dXdd9vqai=hGuQ8kuc9pgc9s8qqaq=dirpe0xb9q8qiLsFr0=vr0=vr0dc8meaabaqaciaacaGaaeqabaWaaeGaeaaakeaaimaacqWFse=uaaa@3845@, EXMOTIF computes the pos-list for each ℳ
 MathType@MTEF@5@5@+=feaafiart1ev1aaatCvAUfKttLearuWrP9MDH5MBPbIqV92AaeXatLxBI9gBamrtHrhAL1wy0L2yHvtyaeHbnfgDOvwBHrxAJfwnaebbnrfifHhDYfgasaacH8akY=wiFfYdH8Gipec8Eeeu0xXdbba9frFj0=OqFfea0dXdd9vqai=hGuQ8kuc9pgc9s8qqaq=dirpe0xb9q8qiLsFr0=vr0=vr0dc8meaabaqaciaacaGaaeqabaWaaeGaeaaakeaaimaacqWFZestaaa@3790@ and report ℳ
 MathType@MTEF@5@5@+=feaafiart1ev1aaatCvAUfKttLearuWrP9MDH5MBPbIqV92AaeXatLxBI9gBamrtHrhAL1wy0L2yHvtyaeHbnfgDOvwBHrxAJfwnaebbnrfifHhDYfgasaacH8akY=wiFfYdH8Gipec8Eeeu0xXdbba9frFj0=OqFfea0dXdd9vqai=hGuQ8kuc9pgc9s8qqaq=dirpe0xb9q8qiLsFr0=vr0=vr0dc8meaabaqaciaacaGaaeqabaWaaeGaeaaakeaaimaacqWFZestaaa@3790@ only if its support is no less than the quorum (*q*). A straightforward approach is to directly perform positional joins on the symbols from the end to the start for each ℳ
 MathType@MTEF@5@5@+=feaafiart1ev1aaatCvAUfKttLearuWrP9MDH5MBPbIqV92AaeXatLxBI9gBamrtHrhAL1wy0L2yHvtyaeHbnfgDOvwBHrxAJfwnaebbnrfifHhDYfgasaacH8akY=wiFfYdH8Gipec8Eeeu0xXdbba9frFj0=OqFfea0dXdd9vqai=hGuQ8kuc9pgc9s8qqaq=dirpe0xb9q8qiLsFr0=vr0=vr0dc8meaabaqaciaacaGaaeqabaWaaeGaeaaakeaaimaacqWFZestaaa@3790@. This approach leads to much redundant computation since simple motif components may be shared among several structured motifs. EXMOTIF, in contrast, performs two steps: it first computes the pos-lists for all simple motifs in S
 MathType@MTEF@5@5@+=feaafiart1ev1aaatCvAUfKttLearuWrP9MDH5MBPbIqV92AaeXatLxBI9gBamrtHrhAL1wy0L2yHvtyaeHbnfgDOvwBHrxAJfwnaebbnrfifHhDYfgasaacH8akY=wiFfYdH8Gipec8Eeeu0xXdbba9frFj0=OqFfea0dXdd9vqai=hGuQ8kuc9pgc9s8qqaq=dirpe0xb9q8qiLsFr0=vr0=vr0dc8meaabaqaciaacaGaaeqabaWaaeGaeaaakeaaimaacqWFse=uaaa@3845@ by doing positional joins on pos-lists of its symbols, and it then computes the pos-list for each structured motif by doing positional joins on pos-lists of its simple motif components. EXMOTIF handles both simple and structured motifs uniformly, by adding the gap range [0, 0] between adjacent symbols within each simple motif *M*_*i*_. For our example in Table 1, the structured motif template T
 MathType@MTEF@5@5@+=feaafiart1ev1aaatCvAUfKttLearuWrP9MDH5MBPbIqV92AaeXatLxBI9gBamrtHrhAL1wy0L2yHvtyaeHbnfgDOvwBHrxAJfwnaebbnrfifHhDYfgasaacH8akY=wiFfYdH8Gipec8Eeeu0xXdbba9frFj0=OqFfea0dXdd9vqai=hGuQ8kuc9pgc9s8qqaq=dirpe0xb9q8qiLsFr0=vr0=vr0dc8meaabaqaciaacaGaaeqabaWaaeGaeaaakeaaimaacqWFtepvaaa@3847@ becomes: N[0,0]N[0,0]N[0,1]N[0,0]N[2,3]N[0,0]N[0,0]N[0,0]N. Also since we only report frequent motifs, we can prune the candidate patterns during the positional joins based on the closure property of support (note however that this cannot be done for weighted support).

#### Extraction of the simple motifs

Given a template motif T
 MathType@MTEF@5@5@+=feaafiart1ev1aaatCvAUfKttLearuWrP9MDH5MBPbIqV92AaeXatLxBI9gBamrtHrhAL1wy0L2yHvtyaeHbnfgDOvwBHrxAJfwnaebbnrfifHhDYfgasaacH8akY=wiFfYdH8Gipec8Eeeu0xXdbba9frFj0=OqFfea0dXdd9vqai=hGuQ8kuc9pgc9s8qqaq=dirpe0xb9q8qiLsFr0=vr0=vr0dc8meaabaqaciaacaGaaeqabaWaaeGaeaaakeaaimaacqWFtepvaaa@3847@, we know the lengths of the simple motif components desired. A naive approach is to directly do positional joins on the symbols from the end to the start of each simple motif. However, since some simple motifs are of the same length and the longer simple motifs can be obtained by doing positional joins on the shorter simple motifs/symbols, we can avoid some redundant computation. Note also that the gap range inside the simple motif is always [0,0].

Let ℒ
 MathType@MTEF@5@5@+=feaafiart1ev1aaatCvAUfKttLearuWrP9MDH5MBPbIqV92AaeXatLxBI9gBaebbnrfifHhDYfgasaacH8akY=wiFfYdH8Gipec8Eeeu0xXdbba9frFj0=OqFfea0dXdd9vqai=hGuQ8kuc9pgc9s8qqaq=dirpe0xb9q8qiLsFr0=vr0=vr0dc8meaabaqaciaacaGaaeqabaqabeGadaaakeaat0uy0HwzTfgDPnwy1egaryqtHrhAL1wy0L2yHvdaiqaacqWFsectaaa@376D@ = {*L*_*i*_, 1 ≤ *i *≤ *m*}, where *L*_*i *_is the length of each simple motif in T
 MathType@MTEF@5@5@+=feaafiart1ev1aaatCvAUfKttLearuWrP9MDH5MBPbIqV92AaeXatLxBI9gBamrtHrhAL1wy0L2yHvtyaeHbnfgDOvwBHrxAJfwnaebbnrfifHhDYfgasaacH8akY=wiFfYdH8Gipec8Eeeu0xXdbba9frFj0=OqFfea0dXdd9vqai=hGuQ8kuc9pgc9s8qqaq=dirpe0xb9q8qiLsFr0=vr0=vr0dc8meaabaqaciaacaGaaeqabaWaaeGaeaaakeaaimaacqWFtepvaaa@3847@ and assume ℒ
 MathType@MTEF@5@5@+=feaafiart1ev1aaatCvAUfKttLearuWrP9MDH5MBPbIqV92AaeXatLxBI9gBaebbnrfifHhDYfgasaacH8akY=wiFfYdH8Gipec8Eeeu0xXdbba9frFj0=OqFfea0dXdd9vqai=hGuQ8kuc9pgc9s8qqaq=dirpe0xb9q8qiLsFr0=vr0=vr0dc8meaabaqaciaacaGaaeqabaqabeGadaaakeaat0uy0HwzTfgDPnwy1egaryqtHrhAL1wy0L2yHvdaiqaacqWFsectaaa@376D@ is sorted in the ascending order. For each *L*_*i*_, 1 ≤ *i *≤ *m*, we need to enumerate |ΣDNA|Li
 MathType@MTEF@5@5@+=feaafiart1ev1aaatCvAUfKttLearuWrP9MDH5MBPbIqV92AaeXatLxBI9gBaebbnrfifHhDYfgasaacH8akY=wiFfYdH8Gipec8Eeeu0xXdbba9frFj0=OqFfea0dXdd9vqai=hGuQ8kuc9pgc9s8qqaq=dirpe0xb9q8qiLsFr0=vr0=vr0dc8meaabaqaciaacaGaaeqabaqabeGadaaakeaadaabdaqaaiabfo6atnaaBaaaleaacqqGebarcqqGobGtcqqGbbqqaeqaaaGccaGLhWUaayjcSdWaaWbaaSqabeaacqWGmbatdaWgaaadbaGaemyAaKgabeaaaaaaaa@3799@ possible simple motifs. Let maxℒ
 MathType@MTEF@5@5@+=feaafiart1ev1aaatCvAUfKttLearuWrP9MDH5MBPbIqV92AaeXatLxBI9gBamrtHrhAL1wy0L2yHvtyaeHbnfgDOvwBHrxAJfwnaebbnrfifHhDYfgasaacH8akY=wiFfYdH8Gipec8Eeeu0xXdbba9frFj0=OqFfea0dXdd9vqai=hGuQ8kuc9pgc9s8qqaq=dirpe0xb9q8qiLsFr0=vr0=vr0dc8meaabaqaciaacaGaaeqabaWaaeGaeaaakeaaieGacqWFTbqBcqWFHbqycqWF4baEdaWgaaWcbaacdaGae4NeHWeabeaaaaa@3BBF@ be the maximum length in ℒ
 MathType@MTEF@5@5@+=feaafiart1ev1aaatCvAUfKttLearuWrP9MDH5MBPbIqV92AaeXatLxBI9gBaebbnrfifHhDYfgasaacH8akY=wiFfYdH8Gipec8Eeeu0xXdbba9frFj0=OqFfea0dXdd9vqai=hGuQ8kuc9pgc9s8qqaq=dirpe0xb9q8qiLsFr0=vr0=vr0dc8meaabaqaciaacaGaaeqabaqabeGadaaakeaat0uy0HwzTfgDPnwy1egaryqtHrhAL1wy0L2yHvdaiqaacqWFsectaaa@376D@. We can compute the pos-lists of simple motifs sequentially from length 1 to maxℒ
 MathType@MTEF@5@5@+=feaafiart1ev1aaatCvAUfKttLearuWrP9MDH5MBPbIqV92AaeXatLxBI9gBamrtHrhAL1wy0L2yHvtyaeHbnfgDOvwBHrxAJfwnaebbnrfifHhDYfgasaacH8akY=wiFfYdH8Gipec8Eeeu0xXdbba9frFj0=OqFfea0dXdd9vqai=hGuQ8kuc9pgc9s8qqaq=dirpe0xb9q8qiLsFr0=vr0=vr0dc8meaabaqaciaacaGaaeqabaWaaeGaeaaakeaaieGacqWFTbqBcqWFHbqycqWF4baEdaWgaaWcbaacdaGae4NeHWeabeaaaaa@3BBF@. But this may waste time in enumerating some simple motifs of lengths that are not in ℒ
 MathType@MTEF@5@5@+=feaafiart1ev1aaatCvAUfKttLearuWrP9MDH5MBPbIqV92AaeXatLxBI9gBaebbnrfifHhDYfgasaacH8akY=wiFfYdH8Gipec8Eeeu0xXdbba9frFj0=OqFfea0dXdd9vqai=hGuQ8kuc9pgc9s8qqaq=dirpe0xb9q8qiLsFr0=vr0=vr0dc8meaabaqaciaacaGaaeqabaqabeGadaaakeaat0uy0HwzTfgDPnwy1egaryqtHrhAL1wy0L2yHvdaiqaacqWFsectaaa@376D@. Instead, EXMOTIF first computes the pos-lists for the simple motifs of lengths that are powers of 2. Formally, let *J *be an integer such that 2^*J *^≤ maxℒ
 MathType@MTEF@5@5@+=feaafiart1ev1aaatCvAUfKttLearuWrP9MDH5MBPbIqV92AaeXatLxBI9gBamrtHrhAL1wy0L2yHvtyaeHbnfgDOvwBHrxAJfwnaebbnrfifHhDYfgasaacH8akY=wiFfYdH8Gipec8Eeeu0xXdbba9frFj0=OqFfea0dXdd9vqai=hGuQ8kuc9pgc9s8qqaq=dirpe0xb9q8qiLsFr0=vr0=vr0dc8meaabaqaciaacaGaaeqabaWaaeGaeaaakeaaieGacqWFTbqBcqWFHbqycqWF4baEdaWgaaWcbaacdaGae4NeHWeabeaaaaa@3BBF@ < 2^*J*+1^. We extract the patterns of length 2^*j *^by doing positional joins on the *pos-lists *of patterns of length 2^*j*-1 ^for all 1 ≤ *j *≤ *J*. For example, when maxℒ
 MathType@MTEF@5@5@+=feaafiart1ev1aaatCvAUfKttLearuWrP9MDH5MBPbIqV92AaeXatLxBI9gBamrtHrhAL1wy0L2yHvtyaeHbnfgDOvwBHrxAJfwnaebbnrfifHhDYfgasaacH8akY=wiFfYdH8Gipec8Eeeu0xXdbba9frFj0=OqFfea0dXdd9vqai=hGuQ8kuc9pgc9s8qqaq=dirpe0xb9q8qiLsFr0=vr0=vr0dc8meaabaqaciaacaGaaeqabaWaaeGaeaaakeaaieGacqWFTbqBcqWFHbqycqWF4baEdaWgaaWcbaacdaGae4NeHWeabeaaaaa@3BBF@ = 11, EXMOTIF first computes the pos-lists for simple motifs of length 2^0 ^= 1, 2^1 ^= 2, 2^2 ^= 4 and 2^3 ^= 8.

EXMOTIF then computes the pos-lists for the simple motifs of *L*_*i *_∈ ℒ
 MathType@MTEF@5@5@+=feaafiart1ev1aaatCvAUfKttLearuWrP9MDH5MBPbIqV92AaeXatLxBI9gBaebbnrfifHhDYfgasaacH8akY=wiFfYdH8Gipec8Eeeu0xXdbba9frFj0=OqFfea0dXdd9vqai=hGuQ8kuc9pgc9s8qqaq=dirpe0xb9q8qiLsFr0=vr0=vr0dc8meaabaqaciaacaGaaeqabaqabeGadaaakeaat0uy0HwzTfgDPnwy1egaryqtHrhAL1wy0L2yHvdaiqaacqWFsectaaa@376D@, by doing positional joins on simple motifs whose *pos-list*(s) have already been computed and their lengths sum to *L*_*i*_. For example, when *L*_*i *_= 11, EXMOTIF has to join motifs of lengths 8, 2, and 1. It first obtains all motifs of length 8 + 2 = 10, and then joins the motifs of lengths 10 and 1, to get the pos-lists of all simple motifs of length 10 + 1 = 11. The pos-lists for the simple motifs of length *L*_*i *_∈ ℒ
 MathType@MTEF@5@5@+=feaafiart1ev1aaatCvAUfKttLearuWrP9MDH5MBPbIqV92AaeXatLxBI9gBaebbnrfifHhDYfgasaacH8akY=wiFfYdH8Gipec8Eeeu0xXdbba9frFj0=OqFfea0dXdd9vqai=hGuQ8kuc9pgc9s8qqaq=dirpe0xb9q8qiLsFr0=vr0=vr0dc8meaabaqaciaacaGaaeqabaqabeGadaaakeaat0uy0HwzTfgDPnwy1egaryqtHrhAL1wy0L2yHvdaiqaacqWFsectaaa@376D@ are kept for further use in the structured motif extraction. At the end of the first phase, EXMOTIF has computed the pos-lists for all simple motif components that can satisfy the template.

#### Extraction of the structured motifs

We extract the structured motifs by doing positional joins on the pos-lists of the simple motifs from the end to the start in the structured motif ℳ
 MathType@MTEF@5@5@+=feaafiart1ev1aaatCvAUfKttLearuWrP9MDH5MBPbIqV92AaeXatLxBI9gBamrtHrhAL1wy0L2yHvtyaeHbnfgDOvwBHrxAJfwnaebbnrfifHhDYfgasaacH8akY=wiFfYdH8Gipec8Eeeu0xXdbba9frFj0=OqFfea0dXdd9vqai=hGuQ8kuc9pgc9s8qqaq=dirpe0xb9q8qiLsFr0=vr0=vr0dc8meaabaqaciaacaGaaeqabaWaaeGaeaaakeaaimaacqWFZestaaa@3790@. Formally, let *H*[*l*, *u*]*T *be an intermediate structured motif, with simple motif *H *as the head, and a suffix structured motif *T *as tail. Then P (*H*[*l*, *u*]*T*) can be obtained by doing positional joins on P (*H*) and P (*T*). Since P (*H*) keeps only the start positions, we need to compute the corresponding end positions for those occurrences of *H*, to check the gap constraints. Since only exact matches or substitutions are allowed for simple motifs, the end position is simply *s *+ |*H*| - 1 for a start position *s*.

#### Full-position recovery

In our positional join approach, to save time and space we retain only the motif start positions, however, in some applications, we may need to know the full position of each occurrence, i.e., the set of matching positions for each symbol in the motif. EXMOTIF records some "indices" during the positional joins in order to facilitate full position recovery.

**Figure 2:**
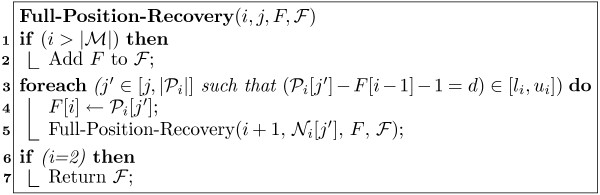
Indexed Full Position Recovery Algorithm.

For each suffix of a structured motif, ℳ
 MathType@MTEF@5@5@+=feaafiart1ev1aaatCvAUfKttLearuWrP9MDH5MBPbIqV92AaeXatLxBI9gBamrtHrhAL1wy0L2yHvtyaeHbnfgDOvwBHrxAJfwnaebbnrfifHhDYfgasaacH8akY=wiFfYdH8Gipec8Eeeu0xXdbba9frFj0=OqFfea0dXdd9vqai=hGuQ8kuc9pgc9s8qqaq=dirpe0xb9q8qiLsFr0=vr0=vr0dc8meaabaqaciaacaGaaeqabaWaaeGaeaaakeaaimaacqWFZestaaa@3790@, starting at position *i *with 1 ≤ *i *≤ |ℳ
 MathType@MTEF@5@5@+=feaafiart1ev1aaatCvAUfKttLearuWrP9MDH5MBPbIqV92AaeXatLxBI9gBamrtHrhAL1wy0L2yHvtyaeHbnfgDOvwBHrxAJfwnaebbnrfifHhDYfgasaacH8akY=wiFfYdH8Gipec8Eeeu0xXdbba9frFj0=OqFfea0dXdd9vqai=hGuQ8kuc9pgc9s8qqaq=dirpe0xb9q8qiLsFr0=vr0=vr0dc8meaabaqaciaacaGaaeqabaWaaeGaeaaakeaaimaacqWFZestaaa@3790@|, we keep its pos-list, P_*i*_, and an index list, N
 MathType@MTEF@5@5@+=feaafiart1ev1aaatCvAUfKttLearuWrP9MDH5MBPbIqV92AaeXatLxBI9gBamrtHrhAL1wy0L2yHvtyaeHbnfgDOvwBHrxAJfwnaebbnrfifHhDYfgasaacH8akY=wiFfYdH8Gipec8Eeeu0xXdbba9frFj0=OqFfea0dXdd9vqai=hGuQ8kuc9pgc9s8qqaq=dirpe0xb9q8qiLsFr0=vr0=vr0dc8meaabaqaciaacaGaaeqabaWaaeGaeaaakeaaimaacqWFneVtaaa@383B@_*i*_. For each entry, say P_*i*_[*j*], in the pos-list P_*i*_, the corresponding index entry N
 MathType@MTEF@5@5@+=feaafiart1ev1aaatCvAUfKttLearuWrP9MDH5MBPbIqV92AaeXatLxBI9gBamrtHrhAL1wy0L2yHvtyaeHbnfgDOvwBHrxAJfwnaebbnrfifHhDYfgasaacH8akY=wiFfYdH8Gipec8Eeeu0xXdbba9frFj0=OqFfea0dXdd9vqai=hGuQ8kuc9pgc9s8qqaq=dirpe0xb9q8qiLsFr0=vr0=vr0dc8meaabaqaciaacaGaaeqabaWaaeGaeaaakeaaimaacqWFneVtaaa@383B@_*i*_[*j*], points to the first entry, say *f*, in P_*i*+1 _that satisfies the gap range with respect to P_*i*_[*j*], i.e., P_*i*+1_[*f*] - P_*i*_[*j*] - 1 ∈ [*l*_*i*_, *u*_*i*_]. Note that N|ℳ|
 MathType@MTEF@5@5@+=feaafiart1ev1aaatCvAUfKttLearuWrP9MDH5MBPbIqV92AaeXatLxBI9gBamrtHrhAL1wy0L2yHvtyaeHbnfgDOvwBHrxAJfwnaebbnrfifHhDYfgasaacH8akY=wiFfYdH8Gipec8Eeeu0xXdbba9frFj0=OqFfea0dXdd9vqai=hGuQ8kuc9pgc9s8qqaq=dirpe0xb9q8qiLsFr0=vr0=vr0dc8meaabaqaciaacaGaaeqabaWaaeGaeaaakeaaimaacqWFneVtdaWgaaWcbaWaaqWaaeaacqWFZestaiaawEa7caGLiWoaaeqaaaaa@3CAF@ is never used. Also note that P (ℳ
 MathType@MTEF@5@5@+=feaafiart1ev1aaatCvAUfKttLearuWrP9MDH5MBPbIqV92AaeXatLxBI9gBamrtHrhAL1wy0L2yHvtyaeHbnfgDOvwBHrxAJfwnaebbnrfifHhDYfgasaacH8akY=wiFfYdH8Gipec8Eeeu0xXdbba9frFj0=OqFfea0dXdd9vqai=hGuQ8kuc9pgc9s8qqaq=dirpe0xb9q8qiLsFr0=vr0=vr0dc8meaabaqaciaacaGaaeqabaWaaeGaeaaakeaaimaacqWFZestaaa@3790@) = P_1_. Let *s *be a start position for the structured motif in sequence *S*, and let *s *be the *j*_*s*_-th entry in P_1_, i.e., *s *= P_1_[*j*_*s*_]. Let *F *store a full position starting from *s*, and let ℱ
 MathType@MTEF@5@5@+=feaafiart1ev1aaatCvAUfKttLearuWrP9MDH5MBPbIqV92AaeXatLxBI9gBamrtHrhAL1wy0L2yHvtyaeHbnfgDOvwBHrxAJfwnaebbnrfifHhDYfgasaacH8akY=wiFfYdH8Gipec8Eeeu0xXdbba9frFj0=OqFfea0dXdd9vqai=hGuQ8kuc9pgc9s8qqaq=dirpe0xb9q8qiLsFr0=vr0=vr0dc8meaabaqaciaacaGaaeqabaWaaeGaeaaakeaaimaacqWFXeIraaa@3787@ store the set of all full positions. Figure 2 shows the pseudo-code for recovering full positions starting from *s*. This recursive algorithm has four parameters: *i *denotes a (suffix) position in ℳ
 MathType@MTEF@5@5@+=feaafiart1ev1aaatCvAUfKttLearuWrP9MDH5MBPbIqV92AaeXatLxBI9gBamrtHrhAL1wy0L2yHvtyaeHbnfgDOvwBHrxAJfwnaebbnrfifHhDYfgasaacH8akY=wiFfYdH8Gipec8Eeeu0xXdbba9frFj0=OqFfea0dXdd9vqai=hGuQ8kuc9pgc9s8qqaq=dirpe0xb9q8qiLsFr0=vr0=vr0dc8meaabaqaciaacaGaaeqabaWaaeGaeaaakeaaimaacqWFZestaaa@3790@, *j *gives the *j*-th entry in P_*i*_, *F *denotes an intermediate full position, and ℱ
 MathType@MTEF@5@5@+=feaafiart1ev1aaatCvAUfKttLearuWrP9MDH5MBPbIqV92AaeXatLxBI9gBamrtHrhAL1wy0L2yHvtyaeHbnfgDOvwBHrxAJfwnaebbnrfifHhDYfgasaacH8akY=wiFfYdH8Gipec8Eeeu0xXdbba9frFj0=OqFfea0dXdd9vqai=hGuQ8kuc9pgc9s8qqaq=dirpe0xb9q8qiLsFr0=vr0=vr0dc8meaabaqaciaacaGaaeqabaWaaeGaeaaakeaaimaacqWFXeIraaa@3787@ denotes the set of all the full occurrences. The algorithm is initially called with *i *= 2, *j *= N
 MathType@MTEF@5@5@+=feaafiart1ev1aaatCvAUfKttLearuWrP9MDH5MBPbIqV92AaeXatLxBI9gBamrtHrhAL1wy0L2yHvtyaeHbnfgDOvwBHrxAJfwnaebbnrfifHhDYfgasaacH8akY=wiFfYdH8Gipec8Eeeu0xXdbba9frFj0=OqFfea0dXdd9vqai=hGuQ8kuc9pgc9s8qqaq=dirpe0xb9q8qiLsFr0=vr0=vr0dc8meaabaqaciaacaGaaeqabaWaaeGaeaaakeaaimaacqWFneVtaaa@383B@_1_[*j*_*s*_], *F *= {*s*}, and ℱ
 MathType@MTEF@5@5@+=feaafiart1ev1aaatCvAUfKttLearuWrP9MDH5MBPbIqV92AaeXatLxBI9gBamrtHrhAL1wy0L2yHvtyaeHbnfgDOvwBHrxAJfwnaebbnrfifHhDYfgasaacH8akY=wiFfYdH8Gipec8Eeeu0xXdbba9frFj0=OqFfea0dXdd9vqai=hGuQ8kuc9pgc9s8qqaq=dirpe0xb9q8qiLsFr0=vr0=vr0dc8meaabaqaciaacaGaaeqabaWaaeGaeaaakeaaimaacqWFXeIraaa@3787@ = ∅. Starting at the first index in *P*_*i*_, that satisfies the gap range with respect to the last position in *F*, we continue to compute all such positions *j*' ∈ [*j*, |*P*_*i*_|] that satisfy the gap range (line 3).  That is, we find all positions *j*', such that *P*_*i*_[*j*'] - *F*[*i *- 1] - 1 = *d *∈ [*l*_*i*_, *u*_*i*_]. For each such position *j*', we add it in turn to the intermediate full position, and make another recursive call (line 5), passing the first index position *N*_*i*_[*j*'] in *P*_*i*+1 _that can satisfy the gap range with respect to *P*_*i*_[*j*']. Thus in each call we keep following the indices from one pos-list to the next, to finally obtain a full position starting from *s *when we reach the last pos-list, ${\text{P}}_{\left|ℳ\right|}$. Note that at each suffix position *i*, since *j *only marks the first position in P_*i*+1 _that satisfies the gap constraints, we also need to consider all the subsequent positions *j*' > *j *that may satisfy the corresponding gap range.

**Figure 3:**
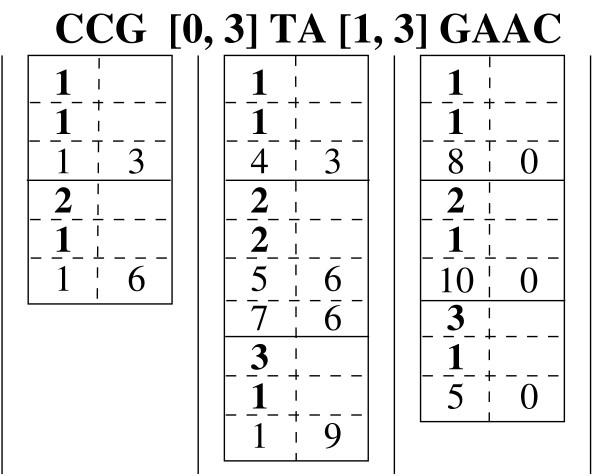
Indexed Full-position Recovery Example.

Consider the example shown in Fig. 3 to recover the full positions for ℳ
 MathType@MTEF@5@5@+=feaafiart1ev1aaatCvAUfKttLearuWrP9MDH5MBPbIqV92AaeXatLxBI9gBamrtHrhAL1wy0L2yHvtyaeHbnfgDOvwBHrxAJfwnaebbnrfifHhDYfgasaacH8akY=wiFfYdH8Gipec8Eeeu0xXdbba9frFj0=OqFfea0dXdd9vqai=hGuQ8kuc9pgc9s8qqaq=dirpe0xb9q8qiLsFr0=vr0=vr0dc8meaabaqaciaacaGaaeqabaWaaeGaeaaakeaaimaacqWFZestaaa@3790@ = CCG[0,3]TA[1,3]GAAC. Under each symbol we show two columns. The left column corresponds to the intermediate pos-lists as we proceed from right to left, whereas the right column stores the indices into the previous pos-list. For example, the middle column gives the pos-list P (*TA*[1,3]*GAAC*) = {**1**, **1**, 4, **2**, **2**, 5, 7, **3**, **1**, 1}. For each position *x *∈ P (*TA*[l,3]*GAAC*) (excluding the sequence identifiers and the cardinality), the right column records an index in P (*GAAC*) which corresponds to the first position in P (*GAAC*) that satisfies the gap range with respect to *x*. For example, for position *x *= 5 (at index 6), the first position in P (*GAAC*) that satisfies the gap range [1,3] is 10 (since in this case there are 3 gaps between the end of TA at position 6 and start of GAAC at position 10), and it occurs at index 6. Likewise, for each position in the current pos-list we store which positions in the previous pos-list were extended. With this indexed information, full-position recovery becomes straightforward. We begin with the start positions of the occurrences. We then keep following the indices from one pos-list to the next, until we reach the last pos-list. Since the index only marks the first position that satisfies the gap range, we still need to check if the following positions satisfy the gap range. At each stage in the full position recovery, we maintain a list of intermediate position prefixes ℱ
 MathType@MTEF@5@5@+=feaafiart1ev1aaatCvAUfKttLearuWrP9MDH5MBPbIqV92AaeXatLxBI9gBamrtHrhAL1wy0L2yHvtyaeHbnfgDOvwBHrxAJfwnaebbnrfifHhDYfgasaacH8akY=wiFfYdH8Gipec8Eeeu0xXdbba9frFj0=OqFfea0dXdd9vqai=hGuQ8kuc9pgc9s8qqaq=dirpe0xb9q8qiLsFr0=vr0=vr0dc8meaabaqaciaacaGaaeqabaWaaeGaeaaakeaaimaacqWFXeIraaa@3787@ that match up to the *j*-th position in ℳ
 MathType@MTEF@5@5@+=feaafiart1ev1aaatCvAUfKttLearuWrP9MDH5MBPbIqV92AaeXatLxBI9gBamrtHrhAL1wy0L2yHvtyaeHbnfgDOvwBHrxAJfwnaebbnrfifHhDYfgasaacH8akY=wiFfYdH8Gipec8Eeeu0xXdbba9frFj0=OqFfea0dXdd9vqai=hGuQ8kuc9pgc9s8qqaq=dirpe0xb9q8qiLsFr0=vr0=vr0dc8meaabaqaciaacaGaaeqabaWaaeGaeaaakeaaimaacqWFZestaaa@3790@. For example, to recover the full position for ℳ
 MathType@MTEF@5@5@+=feaafiart1ev1aaatCvAUfKttLearuWrP9MDH5MBPbIqV92AaeXatLxBI9gBamrtHrhAL1wy0L2yHvtyaeHbnfgDOvwBHrxAJfwnaebbnrfifHhDYfgasaacH8akY=wiFfYdH8Gipec8Eeeu0xXdbba9frFj0=OqFfea0dXdd9vqai=hGuQ8kuc9pgc9s8qqaq=dirpe0xb9q8qiLsFr0=vr0=vr0dc8meaabaqaciaacaGaaeqabaWaaeGaeaaakeaaimaacqWFZestaaa@3790@ = CCG[0,3]TA[1,3]GAAC, considering start position 1 (with ℱ
 MathType@MTEF@5@5@+=feaafiart1ev1aaatCvAUfKttLearuWrP9MDH5MBPbIqV92AaeXatLxBI9gBamrtHrhAL1wy0L2yHvtyaeHbnfgDOvwBHrxAJfwnaebbnrfifHhDYfgasaacH8akY=wiFfYdH8Gipec8Eeeu0xXdbba9frFj0=OqFfea0dXdd9vqai=hGuQ8kuc9pgc9s8qqaq=dirpe0xb9q8qiLsFr0=vr0=vr0dc8meaabaqaciaacaGaaeqabaWaaeGaeaaakeaaimaacqWFXeIraaa@3787@ = {(1)}) in sequence 2, we follow index 6 to get position 5 in the middle pos-list, to get ℱ
 MathType@MTEF@5@5@+=feaafiart1ev1aaatCvAUfKttLearuWrP9MDH5MBPbIqV92AaeXatLxBI9gBamrtHrhAL1wy0L2yHvtyaeHbnfgDOvwBHrxAJfwnaebbnrfifHhDYfgasaacH8akY=wiFfYdH8Gipec8Eeeu0xXdbba9frFj0=OqFfea0dXdd9vqai=hGuQ8kuc9pgc9s8qqaq=dirpe0xb9q8qiLsFr0=vr0=vr0dc8meaabaqaciaacaGaaeqabaWaaeGaeaaakeaaimaacqWFXeIraaa@3787@ = {(1, 5)}. Since the next position after 5 is 7 which is also within the gap range [0,3], so we update ℱ
 MathType@MTEF@5@5@+=feaafiart1ev1aaatCvAUfKttLearuWrP9MDH5MBPbIqV92AaeXatLxBI9gBamrtHrhAL1wy0L2yHvtyaeHbnfgDOvwBHrxAJfwnaebbnrfifHhDYfgasaacH8akY=wiFfYdH8Gipec8Eeeu0xXdbba9frFj0=OqFfea0dXdd9vqai=hGuQ8kuc9pgc9s8qqaq=dirpe0xb9q8qiLsFr0=vr0=vr0dc8meaabaqaciaacaGaaeqabaWaaeGaeaaakeaaimaacqWFXeIraaa@3787@ = {(1, 5), (1, 7)}. For position 5, we follow index 6 to get position 10 in the rightmost pos-list, to get ℱ
 MathType@MTEF@5@5@+=feaafiart1ev1aaatCvAUfKttLearuWrP9MDH5MBPbIqV92AaeXatLxBI9gBamrtHrhAL1wy0L2yHvtyaeHbnfgDOvwBHrxAJfwnaebbnrfifHhDYfgasaacH8akY=wiFfYdH8Gipec8Eeeu0xXdbba9frFj0=OqFfea0dXdd9vqai=hGuQ8kuc9pgc9s8qqaq=dirpe0xb9q8qiLsFr0=vr0=vr0dc8meaabaqaciaacaGaaeqabaWaaeGaeaaakeaaimaacqWFXeIraaa@3787@ = {(1, 5, 10)}; for position 7, we follow index 6 to get position 10 in the right pos-list, to get ℱ
 MathType@MTEF@5@5@+=feaafiart1ev1aaatCvAUfKttLearuWrP9MDH5MBPbIqV92AaeXatLxBI9gBamrtHrhAL1wy0L2yHvtyaeHbnfgDOvwBHrxAJfwnaebbnrfifHhDYfgasaacH8akY=wiFfYdH8Gipec8Eeeu0xXdbba9frFj0=OqFfea0dXdd9vqai=hGuQ8kuc9pgc9s8qqaq=dirpe0xb9q8qiLsFr0=vr0=vr0dc8meaabaqaciaacaGaaeqabaWaaeGaeaaakeaaimaacqWFXeIraaa@3787@ = {(1, 7, 10)}. Likewise, we can recover the full-position in sequence 1, which is ℱ
 MathType@MTEF@5@5@+=feaafiart1ev1aaatCvAUfKttLearuWrP9MDH5MBPbIqV92AaeXatLxBI9gBamrtHrhAL1wy0L2yHvtyaeHbnfgDOvwBHrxAJfwnaebbnrfifHhDYfgasaacH8akY=wiFfYdH8Gipec8Eeeu0xXdbba9frFj0=OqFfea0dXdd9vqai=hGuQ8kuc9pgc9s8qqaq=dirpe0xb9q8qiLsFr0=vr0=vr0dc8meaabaqaciaacaGaaeqabaWaaeGaeaaakeaaimaacqWFXeIraaa@3787@ = {(1, 4, 8)}. During the full-position recovery, we can also count the number of full-positions, i.e., occurrences, of each structured motif. For example, there are 3 occurrences of CCG[0,3]TA[1,3]GAAC.

#### Length ranges for simple motifs

EXMOTIF also allows variation in the lengths of the simple motifs to be found. For example, a motif template may be specified as *M*_1_[5,10] *M*_2_, |*M*_1_| ∈ [2,4], and |*M*_2_| ∈ [6,7], which means that we have to consider NN, NNN, and NNNN as the possible templates for *M*_1 _and similarly for *M*_2_. A straightforward way for handling length ranges is to enumerate exhaustively all the possible sub-templates of T
 MathType@MTEF@5@5@+=feaafiart1ev1aaatCvAUfKttLearuWrP9MDH5MBPbIqV92AaeXatLxBI9gBamrtHrhAL1wy0L2yHvtyaeHbnfgDOvwBHrxAJfwnaebbnrfifHhDYfgasaacH8akY=wiFfYdH8Gipec8Eeeu0xXdbba9frFj0=OqFfea0dXdd9vqai=hGuQ8kuc9pgc9s8qqaq=dirpe0xb9q8qiLsFr0=vr0=vr0dc8meaabaqaciaacaGaaeqabaWaaeGaeaaakeaaimaacqWFtepvaaa@3847@ with simple motifs of fixed lengths and then to extract each sub-template separately. Instead, EXMOTIF does an optimized extraction. EXMOTIF reuses the partial pos-lists created when using a depth first search to enumerate and extract the sub-templates.

### Handling substitutions

As mutations are a common phenomena in biological sequences, we allow substitutions in the extracted motifs. That is two motif instances may be considered to be the same if they are within the allowed substitution thresholds. EXMOTIF allows users to specify the number of substitutions allowed for the whole motif (*ε*), and also a per simple motif threshold (*ε*_*i*_, *i *∈ [1, *k*]). There are two types of substitutions we consider.

#### Position-specific substitutions

Here we allow a position (a DNA symbol) in the instance motif ℳ
 MathType@MTEF@5@5@+=feaafiart1ev1aaatCvAUfKttLearuWrP9MDH5MBPbIqV92AaeXatLxBI9gBamrtHrhAL1wy0L2yHvtyaeHbnfgDOvwBHrxAJfwnaebbnrfifHhDYfgasaacH8akY=wiFfYdH8Gipec8Eeeu0xXdbba9frFj0=OqFfea0dXdd9vqai=hGuQ8kuc9pgc9s8qqaq=dirpe0xb9q8qiLsFr0=vr0=vr0dc8meaabaqaciaacaGaaeqabaWaaeGaeaaakeaaimaacqWFZestaaa@3790@ to be substituted with 1 or 2 other DNA symbols. All such neighbors will contribute to the frequency of ℳ
 MathType@MTEF@5@5@+=feaafiart1ev1aaatCvAUfKttLearuWrP9MDH5MBPbIqV92AaeXatLxBI9gBamrtHrhAL1wy0L2yHvtyaeHbnfgDOvwBHrxAJfwnaebbnrfifHhDYfgasaacH8akY=wiFfYdH8Gipec8Eeeu0xXdbba9frFj0=OqFfea0dXdd9vqai=hGuQ8kuc9pgc9s8qqaq=dirpe0xb9q8qiLsFr0=vr0=vr0dc8meaabaqaciaacaGaaeqabaWaaeGaeaaakeaaimaacqWFZestaaa@3790@. For example, for ℳ
 MathType@MTEF@5@5@+=feaafiart1ev1aaatCvAUfKttLearuWrP9MDH5MBPbIqV92AaeXatLxBI9gBamrtHrhAL1wy0L2yHvtyaeHbnfgDOvwBHrxAJfwnaebbnrfifHhDYfgasaacH8akY=wiFfYdH8Gipec8Eeeu0xXdbba9frFj0=OqFfea0dXdd9vqai=hGuQ8kuc9pgc9s8qqaq=dirpe0xb9q8qiLsFr0=vr0=vr0dc8meaabaqaciaacaGaaeqabaWaaeGaeaaakeaaimaacqWFZestaaa@3790@ = *ACG*[4,6]*TT*, if we allow *e*_1 _= 1 substitutions in motif *M*_*1 *_= *ACG*, at position 2, then *AAG*[4,6]*TT*, *ACG*[4,6]*TT *or *AGG*[4,6]*TT *may contribute to the frequency of ℳ
 MathType@MTEF@5@5@+=feaafiart1ev1aaatCvAUfKttLearuWrP9MDH5MBPbIqV92AaeXatLxBI9gBamrtHrhAL1wy0L2yHvtyaeHbnfgDOvwBHrxAJfwnaebbnrfifHhDYfgasaacH8akY=wiFfYdH8Gipec8Eeeu0xXdbba9frFj0=OqFfea0dXdd9vqai=hGuQ8kuc9pgc9s8qqaq=dirpe0xb9q8qiLsFr0=vr0=vr0dc8meaabaqaciaacaGaaeqabaWaaeGaeaaakeaaimaacqWFZestaaa@3790@. Instead of enumerating all of these separately, EXMOTIF can directly mine relevant motifs using IUPAC symbols (see Table 3). EXMOTIF simply constructs the pos-lists for the relevant IUPAC symbols by scanning sequences in S
 MathType@MTEF@5@5@+=feaafiart1ev1aaatCvAUfKttLearuWrP9MDH5MBPbIqV92AaeXatLxBI9gBamrtHrhAL1wy0L2yHvtyaeHbnfgDOvwBHrxAJfwnaebbnrfifHhDYfgasaacH8akY=wiFfYdH8Gipec8Eeeu0xXdbba9frFj0=OqFfea0dXdd9vqai=hGuQ8kuc9pgc9s8qqaq=dirpe0xb9q8qiLsFr0=vr0=vr0dc8meaabaqaciaacaGaaeqabaWaaeGaeaaakeaaimaacqWFse=uaaa@3845@ once. Then it mines the motif instances as in the basic approach, since all allowed substitutions have already been incorporated into the relevant IUPAC symbols. Let *v*_*i*_, 1 ≤ *i *≤ *k*, to denote the set of IUPAC symbols that can appear in the motif. When *v*_*i *_= 1 (i.e., each position allows only 1 DNA symbol), the alphabet used is {A, C, G, T}; when *v*_*i *_= 2 (i.e., each position may allow up to 2 DNA symbols), the expanded alphabet is {A, C, G, T, R, Y, K, M, S, W}; and when *v*_*i *_= 3 (i.e., each position may allow up to 3 DNA symbols), the expanded alphabet is {A, C, G, T, R, Y, K, M, S, W, B, D, H, V}. For example, when *v*_1 _= 2, instead of reporting ℳ
 MathType@MTEF@5@5@+=feaafiart1ev1aaatCvAUfKttLearuWrP9MDH5MBPbIqV92AaeXatLxBI9gBamrtHrhAL1wy0L2yHvtyaeHbnfgDOvwBHrxAJfwnaebbnrfifHhDYfgasaacH8akY=wiFfYdH8Gipec8Eeeu0xXdbba9frFj0=OqFfea0dXdd9vqai=hGuQ8kuc9pgc9s8qqaq=dirpe0xb9q8qiLsFr0=vr0=vr0dc8meaabaqaciaacaGaaeqabaWaaeGaeaaakeaaimaacqWFZestaaa@3790@ = *ACG*[4,6]*TT *as the mined instance, EXMOTIF may report *ASG*[4,6]*TT *as an instance, where S stands for either C or G (see Table 3). EXMOTIF also allows the user to specify the maximum number of IUPAC symbols that can appear in each simple motif, *e*_*i*_, 1 ≤ *i *≤ *k*.

**Table 3:** IUPAC alphabet (Σ_IUPAC_).

Symbol	A	C	G	T
Bases	A	C	G	T
Symbol	U	R	Y	K
Bases	U	A,G	C,T	G,T

Symbol	M	S	W	B
Bases	A,C	G,C	A,T	C,G,T

Symbol	D	H	V	N
Bases	A,G,T	A,C,T	A,C,G	A,C,G,T

#### Arbitrary substitutions

Here we allow a DNA symbol in ℳ
 MathType@MTEF@5@5@+=feaafiart1ev1aaatCvAUfKttLearuWrP9MDH5MBPbIqV92AaeXatLxBI9gBamrtHrhAL1wy0L2yHvtyaeHbnfgDOvwBHrxAJfwnaebbnrfifHhDYfgasaacH8akY=wiFfYdH8Gipec8Eeeu0xXdbba9frFj0=OqFfea0dXdd9vqai=hGuQ8kuc9pgc9s8qqaq=dirpe0xb9q8qiLsFr0=vr0=vr0dc8meaabaqaciaacaGaaeqabaWaaeGaeaaakeaaimaacqWFZestaaa@3790@ to be substituted with other symbols across all positions (i.e., in a position independent manner), up to the allowed maximum errors per motif (or per component). To count the support for a motif, EXMOTIF has to consider all of its *neighbors *as well, which are defined as all the motifs (including itself) within *Hamming distance, ε *(or per motif *e*_*i*_). Then the support of an instance motif is calculated as the total number of sequences in which its neighbors (including itself) are present. As always, the motif is frequent if its support meets the quorum *q*, that is, its neighbors are present in at least *q *distinct sequences.

The main challenge is that when arbitrary, position independent substitutions are allowed, we cannot do support checking during each positional join, since the support of the current motif may be below quorum, but combined with its neighbors it may meet quorum. Thus EXMOTIF does support checking at two points. First, it checks for quorum after the pos-lists of all the simple motifs in T
 MathType@MTEF@5@5@+=feaafiart1ev1aaatCvAUfKttLearuWrP9MDH5MBPbIqV92AaeXatLxBI9gBamrtHrhAL1wy0L2yHvtyaeHbnfgDOvwBHrxAJfwnaebbnrfifHhDYfgasaacH8akY=wiFfYdH8Gipec8Eeeu0xXdbba9frFj0=OqFfea0dXdd9vqai=hGuQ8kuc9pgc9s8qqaq=dirpe0xb9q8qiLsFr0=vr0=vr0dc8meaabaqaciaacaGaaeqabaWaaeGaeaaakeaaimaacqWFtepvaaa@3847@ have been computed, provided the per motif error thresholds *e*_*i *_have been specified. In this case each simple motif must be frequent to be extended to a structured motif. Second, it checks for quorum after the pos-lists of all the structured motifs that satisfy T
 MathType@MTEF@5@5@+=feaafiart1ev1aaatCvAUfKttLearuWrP9MDH5MBPbIqV92AaeXatLxBI9gBamrtHrhAL1wy0L2yHvtyaeHbnfgDOvwBHrxAJfwnaebbnrfifHhDYfgasaacH8akY=wiFfYdH8Gipec8Eeeu0xXdbba9frFj0=OqFfea0dXdd9vqai=hGuQ8kuc9pgc9s8qqaq=dirpe0xb9q8qiLsFr0=vr0=vr0dc8meaabaqaciaacaGaaeqabaWaaeGaeaaakeaaimaacqWFtepvaaa@3847@ are computed.

##### Determining neighbors

In order to quickly find all the existing neighbors of a motif within the allowed error thresholds, EXMOTIF first computes all the exact structured motifs, and stores them into a hash table to facilitate fast lookup. Then for each extracted structured motif ℳ
 MathType@MTEF@5@5@+=feaafiart1ev1aaatCvAUfKttLearuWrP9MDH5MBPbIqV92AaeXatLxBI9gBamrtHrhAL1wy0L2yHvtyaeHbnfgDOvwBHrxAJfwnaebbnrfifHhDYfgasaacH8akY=wiFfYdH8Gipec8Eeeu0xXdbba9frFj0=OqFfea0dXdd9vqai=hGuQ8kuc9pgc9s8qqaq=dirpe0xb9q8qiLsFr0=vr0=vr0dc8meaabaqaciaacaGaaeqabaWaaeGaeaaakeaaimaacqWFZestaaa@3790@, EXMOTIF enumerates all its possible neighbors and checks whether they exist in the hash table. One problem is that the number of possible neighbors of ℳ
 MathType@MTEF@5@5@+=feaafiart1ev1aaatCvAUfKttLearuWrP9MDH5MBPbIqV92AaeXatLxBI9gBamrtHrhAL1wy0L2yHvtyaeHbnfgDOvwBHrxAJfwnaebbnrfifHhDYfgasaacH8akY=wiFfYdH8Gipec8Eeeu0xXdbba9frFj0=OqFfea0dXdd9vqai=hGuQ8kuc9pgc9s8qqaq=dirpe0xb9q8qiLsFr0=vr0=vr0dc8meaabaqaciaacaGaaeqabaWaaeGaeaaakeaaimaacqWFZestaaa@3790@ can be quite large. When we allow *ε*_*i *_substitutions for simple component *M*_*i *_in ℳ
 MathType@MTEF@5@5@+=feaafiart1ev1aaatCvAUfKttLearuWrP9MDH5MBPbIqV92AaeXatLxBI9gBamrtHrhAL1wy0L2yHvtyaeHbnfgDOvwBHrxAJfwnaebbnrfifHhDYfgasaacH8akY=wiFfYdH8Gipec8Eeeu0xXdbba9frFj0=OqFfea0dXdd9vqai=hGuQ8kuc9pgc9s8qqaq=dirpe0xb9q8qiLsFr0=vr0=vr0dc8meaabaqaciaacaGaaeqabaWaaeGaeaaakeaaimaacqWFZestaaa@3790@, for 1 ≤ *i *≤ *k*, the number of ℳ
 MathType@MTEF@5@5@+=feaafiart1ev1aaatCvAUfKttLearuWrP9MDH5MBPbIqV92AaeXatLxBI9gBamrtHrhAL1wy0L2yHvtyaeHbnfgDOvwBHrxAJfwnaebbnrfifHhDYfgasaacH8akY=wiFfYdH8Gipec8Eeeu0xXdbba9frFj0=OqFfea0dXdd9vqai=hGuQ8kuc9pgc9s8qqaq=dirpe0xb9q8qiLsFr0=vr0=vr0dc8meaabaqaciaacaGaaeqabaWaaeGaeaaakeaaimaacqWFZestaaa@3790@'s neighbors is given as ∏i=1k[∑j=0ei(|Mi|j)⋅3j]
MathType@MTEF@5@5@+=feaafiart1ev1aaatCvAUfKttLearuWrP9MDH5MBPbIqV92AaeXatLxBI9gBaebbnrfifHhDYfgasaacH8akY=wiFfYdH8Gipec8Eeeu0xXdbba9frFj0=OqFfea0dXdd9vqai=hGuQ8kuc9pgc9s8qqaq=dirpe0xb9q8qiLsFr0=vr0=vr0dc8meaabaqaciaacaGaaeqabaqabeGadaaakeaadaqeWaqaaiabcUfaBnaaqadabaWaaeWaaeaafaqabeGabaaabaWaaqWaaeaacqWGnbqtdaWgaaWcbaGaemyAaKgabeaaaOGaay5bSlaawIa7aaqaaiabdQgaQbaaaiaawIcacaGLPaaacqGHflY1cqaIZaWmdaahaaWcbeqaaiabdQgaQbaaaeaacqWGQbGAcqGH9aqpcqaIWaamaeaacqWGLbqzdaWgaaadbaGaemyAaKgabeaaa0GaeyyeIuoakiabc2faDbWcbaGaemyAaKMaeyypa0JaeGymaedabaGaem4AaSganiabg+Givdaaaa@4B8B@. For example, for ℳ
 MathType@MTEF@5@5@+=feaafiart1ev1aaatCvAUfKttLearuWrP9MDH5MBPbIqV92AaeXatLxBI9gBamrtHrhAL1wy0L2yHvtyaeHbnfgDOvwBHrxAJfwnaebbnrfifHhDYfgasaacH8akY=wiFfYdH8Gipec8Eeeu0xXdbba9frFj0=OqFfea0dXdd9vqai=hGuQ8kuc9pgc9s8qqaq=dirpe0xb9q8qiLsFr0=vr0=vr0dc8meaabaqaciaacaGaaeqabaWaaeGaeaaakeaaimaacqWFZestaaa@3790@ = AACGTT[1,5]AGTTCC, when we allow one substitution for each simple motif, the number of its neighbors is 361; when we allow two substitutions per component, the number of its neighbors is 23,716. Instead of enumerating the potentially large number of neighbors (many of which may not even occur in the sequence set S
 MathType@MTEF@5@5@+=feaafiart1ev1aaatCvAUfKttLearuWrP9MDH5MBPbIqV92AaeXatLxBI9gBamrtHrhAL1wy0L2yHvtyaeHbnfgDOvwBHrxAJfwnaebbnrfifHhDYfgasaacH8akY=wiFfYdH8Gipec8Eeeu0xXdbba9frFj0=OqFfea0dXdd9vqai=hGuQ8kuc9pgc9s8qqaq=dirpe0xb9q8qiLsFr0=vr0=vr0dc8meaabaqaciaacaGaaeqabaWaaeGaeaaakeaaimaacqWFse=uaaa@3845@) for each structured motif ℳ
 MathType@MTEF@5@5@+=feaafiart1ev1aaatCvAUfKttLearuWrP9MDH5MBPbIqV92AaeXatLxBI9gBamrtHrhAL1wy0L2yHvtyaeHbnfgDOvwBHrxAJfwnaebbnrfifHhDYfgasaacH8akY=wiFfYdH8Gipec8Eeeu0xXdbba9frFj0=OqFfea0dXdd9vqai=hGuQ8kuc9pgc9s8qqaq=dirpe0xb9q8qiLsFr0=vr0=vr0dc8meaabaqaciaacaGaaeqabaWaaeGaeaaakeaaimaacqWFZestaaa@3790@ individually, EXMOTIF utilizes the observation that many motifs have shared neighbors, and thus previously computed support information can be reused. EXMOTIF enumerates neighbors in two steps. In the first step, for each ℳ
 MathType@MTEF@5@5@+=feaafiart1ev1aaatCvAUfKttLearuWrP9MDH5MBPbIqV92AaeXatLxBI9gBamrtHrhAL1wy0L2yHvtyaeHbnfgDOvwBHrxAJfwnaebbnrfifHhDYfgasaacH8akY=wiFfYdH8Gipec8Eeeu0xXdbba9frFj0=OqFfea0dXdd9vqai=hGuQ8kuc9pgc9s8qqaq=dirpe0xb9q8qiLsFr0=vr0=vr0dc8meaabaqaciaacaGaaeqabaWaaeGaeaaakeaaimaacqWFZestaaa@3790@, it enumerates *aggregate *neighbor motifs, replacing the allowed number of errors *e*_*i *_with as many 'N' symbols (which stands for A,C,G, or T). The number of possible aggregate neighbors is given as ∏i=1k(|Mi|εi)
MathType@MTEF@5@5@+=feaafiart1ev1aaatCvAUfKttLearuWrP9MDH5MBPbIqV92AaeXatLxBI9gBaebbnrfifHhDYfgasaacH8akY=wiFfYdH8Gipec8Eeeu0xXdbba9frFj0=OqFfea0dXdd9vqai=hGuQ8kuc9pgc9s8qqaq=dirpe0xb9q8qiLsFr0=vr0=vr0dc8meaabaqaciaacaGaaeqabaqabeGadaaakeaadaqeWaqaamaabmaabaqbaeqabiqaaaqaamaaemaabaGaemyta00aaSbaaSqaaiabdMgaPbqabaaakiaawEa7caGLiWoaaeaaiiGacqWF1oqzdaWgaaWcbaGaemyAaKgabeaaaaaakiaawIcacaGLPaaaaSqaaiabdMgaPjabg2da9iabigdaXaqaaiabdUgaRbqdcqGHpis1aaaa@3DF8@. The second step, it computes the support for each aggregate neighbor by expanding each 'N' with each DNA symbol, looking up the hash table for the support of the corresponding motif, and adding the supports for all matching motifs. Since the motifs matching an aggregate are also neighbors of each other, the support of the aggregate can be re-used to compute the support of other matching motifs as well. Once the supports for all aggregate neighbors have been computed, the final support of the structured motif ℳ
 MathType@MTEF@5@5@+=feaafiart1ev1aaatCvAUfKttLearuWrP9MDH5MBPbIqV92AaeXatLxBI9gBamrtHrhAL1wy0L2yHvtyaeHbnfgDOvwBHrxAJfwnaebbnrfifHhDYfgasaacH8akY=wiFfYdH8Gipec8Eeeu0xXdbba9frFj0=OqFfea0dXdd9vqai=hGuQ8kuc9pgc9s8qqaq=dirpe0xb9q8qiLsFr0=vr0=vr0dc8meaabaqaciaacaGaaeqabaWaaeGaeaaakeaaimaacqWFZestaaa@3790@ can be obtained. Thus for each ℳ
 MathType@MTEF@5@5@+=feaafiart1ev1aaatCvAUfKttLearuWrP9MDH5MBPbIqV92AaeXatLxBI9gBamrtHrhAL1wy0L2yHvtyaeHbnfgDOvwBHrxAJfwnaebbnrfifHhDYfgasaacH8akY=wiFfYdH8Gipec8Eeeu0xXdbba9frFj0=OqFfea0dXdd9vqai=hGuQ8kuc9pgc9s8qqaq=dirpe0xb9q8qiLsFr0=vr0=vr0dc8meaabaqaciaacaGaaeqabaWaaeGaeaaakeaaimaacqWFZestaaa@3790@, the number of "neighbors" to consider can be as low as ∏i=1k(|Mi|εi)
MathType@MTEF@5@5@+=feaafiart1ev1aaatCvAUfKttLearuWrP9MDH5MBPbIqV92AaeXatLxBI9gBaebbnrfifHhDYfgasaacH8akY=wiFfYdH8Gipec8Eeeu0xXdbba9frFj0=OqFfea0dXdd9vqai=hGuQ8kuc9pgc9s8qqaq=dirpe0xb9q8qiLsFr0=vr0=vr0dc8meaabaqaciaacaGaaeqabaqabeGadaaakeaadaqeWaqaamaabmaabaqbaeqabiqaaaqaamaaemaabaGaemyta00aaSbaaSqaaiabdMgaPbqabaaakiaawEa7caGLiWoaaeaaiiGacqWF1oqzdaWgaaWcbaGaemyAaKgabeaaaaaakiaawIcacaGLPaaaaSqaaiabdMgaPjabg2da9iabigdaXaqaaiabdUgaRbqdcqGHpis1aaaa@3DF8@!

**Figure 4:**
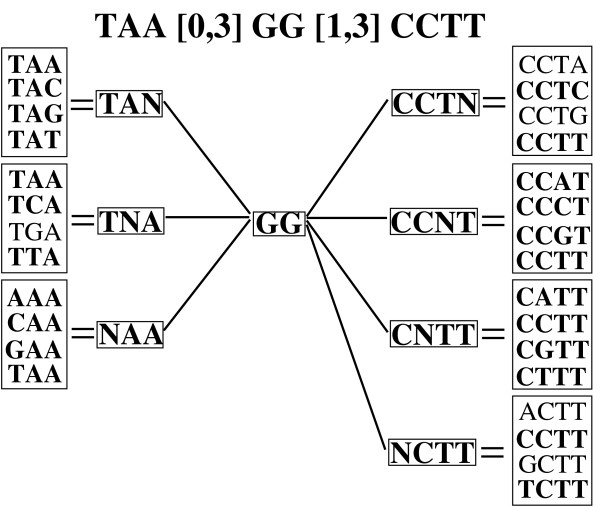
Aggregate Neighbors.

For example, consider the example shown in Figure 4. Consider the structured motif ℳ
 MathType@MTEF@5@5@+=feaafiart1ev1aaatCvAUfKttLearuWrP9MDH5MBPbIqV92AaeXatLxBI9gBamrtHrhAL1wy0L2yHvtyaeHbnfgDOvwBHrxAJfwnaebbnrfifHhDYfgasaacH8akY=wiFfYdH8Gipec8Eeeu0xXdbba9frFj0=OqFfea0dXdd9vqai=hGuQ8kuc9pgc9s8qqaq=dirpe0xb9q8qiLsFr0=vr0=vr0dc8meaabaqaciaacaGaaeqabaWaaeGaeaaakeaaimaacqWFZestaaa@3790@ = TAA[0,3]GG[1,3]CCTT (taken from our example in Table 1); assume that *ε*_1 _= 1, *ε*_2 _= 0 and *ε*_3 _= 1. There are three possible aggregates for TAA, namely TAN, TNA, and NAA, and four aggregates for CCTT, namely CCTN, CCNT, CNTT, and NCTT, giving a total of 12 aggregate neighbors for ℳ
 MathType@MTEF@5@5@+=feaafiart1ev1aaatCvAUfKttLearuWrP9MDH5MBPbIqV92AaeXatLxBI9gBamrtHrhAL1wy0L2yHvtyaeHbnfgDOvwBHrxAJfwnaebbnrfifHhDYfgasaacH8akY=wiFfYdH8Gipec8Eeeu0xXdbba9frFj0=OqFfea0dXdd9vqai=hGuQ8kuc9pgc9s8qqaq=dirpe0xb9q8qiLsFr0=vr0=vr0dc8meaabaqaciaacaGaaeqabaWaaeGaeaaakeaaimaacqWFZestaaa@3790@, as illustrated in the figure. EXMOTIF processes each aggregate neighbor in turn. Using a hash-table (or direct lookup table if there are only a few neighbors), it checks if the aggregate neighbor has been processed previously. If yes, it moves on to the next aggregate. If not, it gathers the support information from all of its matching structured motifs, to compute its total support. Next, it also updates the neighbor support value for each of the matching motifs, so that once an aggregate is processed, we no longer require its information. All we need to know is whether it has been processed or not. For example, once the support of the first aggregate TAN[0,3]GG[1,3]CCTN for the example motif ℳ
 MathType@MTEF@5@5@+=feaafiart1ev1aaatCvAUfKttLearuWrP9MDH5MBPbIqV92AaeXatLxBI9gBamrtHrhAL1wy0L2yHvtyaeHbnfgDOvwBHrxAJfwnaebbnrfifHhDYfgasaacH8akY=wiFfYdH8Gipec8Eeeu0xXdbba9frFj0=OqFfea0dXdd9vqai=hGuQ8kuc9pgc9s8qqaq=dirpe0xb9q8qiLsFr0=vr0=vr0dc8meaabaqaciaacaGaaeqabaWaaeGaeaaakeaaimaacqWFZestaaa@3790@ above is computed, EXMOTIF also updates the neighbor supports for all other matching structured motifs, such as ℳ′
 MathType@MTEF@5@5@+=feaafiart1ev1aaatCvAUfeBSjuyZL2yd9gzLbvyNv2CaerbwvMCKfMBHbqedmvETj2BSbWenfgDOvwBHrxAJfwnHbqeg0uy0HwzTfgDPnwy1aqee0evGueE0jxyaibaieIgFLIOYR2NHOxjYhrPYhrPYpI8F4rqqrFfpeea0xe9Lq=Jc9vqaqpepm0xbbG8FasPYRqj0=yi0lXdbba9pGe9qqFf0dXdHuk9fr=xfr=xfrpiWZqaaeaabiGaaiaacaqabeaadaqacqaaaOqaaGWaaiqb=ntinzaafaaaaa@406D@ = TAC[0,3]GG[1,3]CCTG. Later when processing ℳ′
 MathType@MTEF@5@5@+=feaafiart1ev1aaatCvAUfeBSjuyZL2yd9gzLbvyNv2CaerbwvMCKfMBHbqedmvETj2BSbWenfgDOvwBHrxAJfwnHbqeg0uy0HwzTfgDPnwy1aqee0evGueE0jxyaibaieIgFLIOYR2NHOxjYhrPYhrPYpI8F4rqqrFfpeea0xe9Lq=Jc9vqaqpepm0xbbG8FasPYRqj0=yi0lXdbba9pGe9qqFf0dXdHuk9fr=xfr=xfrpiWZqaaeaabiGaaiaacaqabeaadaqacqaaaOqaaGWaaiqb=ntinzaafaaaaa@406D@, EXMOTIF can skip the above aggregate and focus on the not yet processed aggregates, e.g., NAC[0,3]GG[1,3]NCTG, and so on.

**Figure 5:**
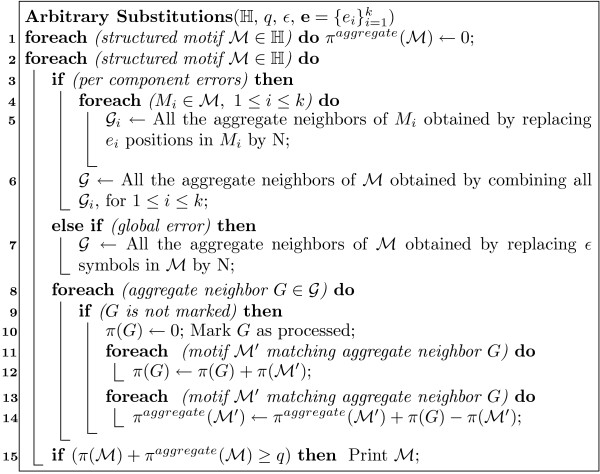
Arbitrary Substitutions.

The pseudo-code for arbitrary substitutions is given in Figure 5. The procedure takes as input the hash-table ℍ containing all structured motifs ℳ
 MathType@MTEF@5@5@+=feaafiart1ev1aaatCvAUfKttLearuWrP9MDH5MBPbIqV92AaeXatLxBI9gBamrtHrhAL1wy0L2yHvtyaeHbnfgDOvwBHrxAJfwnaebbnrfifHhDYfgasaacH8akY=wiFfYdH8Gipec8Eeeu0xXdbba9frFj0=OqFfea0dXdd9vqai=hGuQ8kuc9pgc9s8qqaq=dirpe0xb9q8qiLsFr0=vr0=vr0dc8meaabaqaciaacaGaaeqabaWaaeGaeaaakeaaimaacqWFZestaaa@3790@ and their supports *π*(ℳ
 MathType@MTEF@5@5@+=feaafiart1ev1aaatCvAUfKttLearuWrP9MDH5MBPbIqV92AaeXatLxBI9gBamrtHrhAL1wy0L2yHvtyaeHbnfgDOvwBHrxAJfwnaebbnrfifHhDYfgasaacH8akY=wiFfYdH8Gipec8Eeeu0xXdbba9frFj0=OqFfea0dXdd9vqai=hGuQ8kuc9pgc9s8qqaq=dirpe0xb9q8qiLsFr0=vr0=vr0dc8meaabaqaciaacaGaaeqabaWaaeGaeaaakeaaimaacqWFZestaaa@3790@), the quorum *q*, and the per simple motif errors *e*_*i *_or the global error *ε *for the structured motifs. For each structured motif we also maintain its aggregate support *π*^*aggregate*^(ℳ
 MathType@MTEF@5@5@+=feaafiart1ev1aaatCvAUfKttLearuWrP9MDH5MBPbIqV92AaeXatLxBI9gBamrtHrhAL1wy0L2yHvtyaeHbnfgDOvwBHrxAJfwnaebbnrfifHhDYfgasaacH8akY=wiFfYdH8Gipec8Eeeu0xXdbba9frFj0=OqFfea0dXdd9vqai=hGuQ8kuc9pgc9s8qqaq=dirpe0xb9q8qiLsFr0=vr0=vr0dc8meaabaqaciaacaGaaeqabaWaaeGaeaaakeaaimaacqWFZestaaa@3790@), which is initially set to 0 (line 1). Initially we create all the aggregate neighbors for each extracted structured motif ℳ
 MathType@MTEF@5@5@+=feaafiart1ev1aaatCvAUfKttLearuWrP9MDH5MBPbIqV92AaeXatLxBI9gBamrtHrhAL1wy0L2yHvtyaeHbnfgDOvwBHrxAJfwnaebbnrfifHhDYfgasaacH8akY=wiFfYdH8Gipec8Eeeu0xXdbba9frFj0=OqFfea0dXdd9vqai=hGuQ8kuc9pgc9s8qqaq=dirpe0xb9q8qiLsFr0=vr0=vr0dc8meaabaqaciaacaGaaeqabaWaaeGaeaaakeaaimaacqWFZestaaa@3790@ (lines 3–7). For each such aggregate neighbor *G *(line 8), if it has not been processed, we compute its support by adding the individual supports of all its matching motifs ℳ′
 MathType@MTEF@5@5@+=feaafiart1ev1aaatCvAUfeBSjuyZL2yd9gzLbvyNv2CaerbwvMCKfMBHbqedmvETj2BSbWenfgDOvwBHrxAJfwnHbqeg0uy0HwzTfgDPnwy1aqee0evGueE0jxyaibaieIgFLIOYR2NHOxjYhrPYhrPYpI8F4rqqrFfpeea0xe9Lq=Jc9vqaqpepm0xbbG8FasPYRqj0=yi0lXdbba9pGe9qqFf0dXdHuk9fr=xfr=xfrpiWZqaaeaabiGaaiaacaqabeaadaqacqaaaOqaaGWaaiqb=ntinzaafaaaaa@406D@ (lines 11–12). Note that these support values are found quickly via the hash-table ℍ.  Once the support of an aggregate neighbor is known, we immediately update the aggregate support *π*^*aggregate*^) for each of its contributing matching motifs ℳ′
 MathType@MTEF@5@5@+=feaafiart1ev1aaatCvAUfeBSjuyZL2yd9gzLbvyNv2CaerbwvMCKfMBHbqedmvETj2BSbWenfgDOvwBHrxAJfwnHbqeg0uy0HwzTfgDPnwy1aqee0evGueE0jxyaibaieIgFLIOYR2NHOxjYhrPYhrPYpI8F4rqqrFfpeea0xe9Lq=Jc9vqaqpepm0xbbG8FasPYRqj0=yi0lXdbba9pGe9qqFf0dXdHuk9fr=xfr=xfrpiWZqaaeaabiGaaiaacaqabeaadaqacqaaaOqaaGWaaiqb=ntinzaafaaaaa@406D@ (lines 13–14). Note that since each motif has already contributed to the support of the aggregate neighbor (*π *(*G*)), we must subtract the initial support of ℳ′
 MathType@MTEF@5@5@+=feaafiart1ev1aaatCvAUfeBSjuyZL2yd9gzLbvyNv2CaerbwvMCKfMBHbqedmvETj2BSbWenfgDOvwBHrxAJfwnHbqeg0uy0HwzTfgDPnwy1aqee0evGueE0jxyaibaieIgFLIOYR2NHOxjYhrPYhrPYpI8F4rqqrFfpeea0xe9Lq=Jc9vqaqpepm0xbbG8FasPYRqj0=yi0lXdbba9pGe9qqFf0dXdHuk9fr=xfr=xfrpiWZqaaeaabiGaaiaacaqabeaadaqacqaaaOqaaGWaaiqb=ntinzaafaaaaa@406D@ (*π*(ℳ′
 MathType@MTEF@5@5@+=feaafiart1ev1aaatCvAUfeBSjuyZL2yd9gzLbvyNv2CaerbwvMCKfMBHbqedmvETj2BSbWenfgDOvwBHrxAJfwnHbqeg0uy0HwzTfgDPnwy1aqee0evGueE0jxyaibaieIgFLIOYR2NHOxjYhrPYhrPYpI8F4rqqrFfpeea0xe9Lq=Jc9vqaqpepm0xbbG8FasPYRqj0=yi0lXdbba9pGe9qqFf0dXdHuk9fr=xfr=xfrpiWZqaaeaabiGaaiaacaqabeaadaqacqaaaOqaaGWaaiqb=ntinzaafaaaaa@406D@)) to avoid over-counting. Finally, once all the aggregate neighbors have been processed, we output the structured motif ℳ
 MathType@MTEF@5@5@+=feaafiart1ev1aaatCvAUfKttLearuWrP9MDH5MBPbIqV92AaeXatLxBI9gBamrtHrhAL1wy0L2yHvtyaeHbnfgDOvwBHrxAJfwnaebbnrfifHhDYfgasaacH8akY=wiFfYdH8Gipec8Eeeu0xXdbba9frFj0=OqFfea0dXdd9vqai=hGuQ8kuc9pgc9s8qqaq=dirpe0xb9q8qiLsFr0=vr0=vr0dc8meaabaqaciaacaGaaeqabaWaaeGaeaaakeaaimaacqWFZestaaa@3790@, provided *π*(ℳ
 MathType@MTEF@5@5@+=feaafiart1ev1aaatCvAUfKttLearuWrP9MDH5MBPbIqV92AaeXatLxBI9gBamrtHrhAL1wy0L2yHvtyaeHbnfgDOvwBHrxAJfwnaebbnrfifHhDYfgasaacH8akY=wiFfYdH8Gipec8Eeeu0xXdbba9frFj0=OqFfea0dXdd9vqai=hGuQ8kuc9pgc9s8qqaq=dirpe0xb9q8qiLsFr0=vr0=vr0dc8meaabaqaciaacaGaaeqabaWaaeGaeaaakeaaimaacqWFZestaaa@3790@) + *π*^*aggregate*^(ℳ
 MathType@MTEF@5@5@+=feaafiart1ev1aaatCvAUfKttLearuWrP9MDH5MBPbIqV92AaeXatLxBI9gBamrtHrhAL1wy0L2yHvtyaeHbnfgDOvwBHrxAJfwnaebbnrfifHhDYfgasaacH8akY=wiFfYdH8Gipec8Eeeu0xXdbba9frFj0=OqFfea0dXdd9vqai=hGuQ8kuc9pgc9s8qqaq=dirpe0xb9q8qiLsFr0=vr0=vr0dc8meaabaqaciaacaGaaeqabaWaaeGaeaaakeaaimaacqWFZestaaa@3790@) meets the quorum requirement (line 14).

##### Counting support

There are two methods to record the support for each motif. In the first method, we associate each motif with a bit vector, V
 MathType@MTEF@5@5@+=feaafiart1ev1aaatCvAUfKttLearuWrP9MDH5MBPbIqV92AaeXatLxBI9gBamrtHrhAL1wy0L2yHvtyaeHbnfgDOvwBHrxAJfwnaebbnrfifHhDYfgasaacH8akY=wiFfYdH8Gipec8Eeeu0xXdbba9frFj0=OqFfea0dXdd9vqai=hGuQ8kuc9pgc9s8qqaq=dirpe0xb9q8qiLsFr0=vr0=vr0dc8meaabaqaciaacaGaaeqabaWaaeGaeaaakeaaimaacqWFveVvaaa@384B@. Each bit, V
 MathType@MTEF@5@5@+=feaafiart1ev1aaatCvAUfKttLearuWrP9MDH5MBPbIqV92AaeXatLxBI9gBamrtHrhAL1wy0L2yHvtyaeHbnfgDOvwBHrxAJfwnaebbnrfifHhDYfgasaacH8akY=wiFfYdH8Gipec8Eeeu0xXdbba9frFj0=OqFfea0dXdd9vqai=hGuQ8kuc9pgc9s8qqaq=dirpe0xb9q8qiLsFr0=vr0=vr0dc8meaabaqaciaacaGaaeqabaWaaeGaeaaakeaaimaacqWFveVvaaa@384B@_*i *_for 1 ≤ *i *≤ *n *(where *n *= |S
 MathType@MTEF@5@5@+=feaafiart1ev1aaatCvAUfKttLearuWrP9MDH5MBPbIqV92AaeXatLxBI9gBamrtHrhAL1wy0L2yHvtyaeHbnfgDOvwBHrxAJfwnaebbnrfifHhDYfgasaacH8akY=wiFfYdH8Gipec8Eeeu0xXdbba9frFj0=OqFfea0dXdd9vqai=hGuQ8kuc9pgc9s8qqaq=dirpe0xb9q8qiLsFr0=vr0=vr0dc8meaabaqaciaacaGaaeqabaWaaeGaeaaakeaaimaacqWFse=uaaa@3845@|) indicates whether the motif is present in the sequence *S*_*i *_∈ S
 MathType@MTEF@5@5@+=feaafiart1ev1aaatCvAUfKttLearuWrP9MDH5MBPbIqV92AaeXatLxBI9gBamrtHrhAL1wy0L2yHvtyaeHbnfgDOvwBHrxAJfwnaebbnrfifHhDYfgasaacH8akY=wiFfYdH8Gipec8Eeeu0xXdbba9frFj0=OqFfea0dXdd9vqai=hGuQ8kuc9pgc9s8qqaq=dirpe0xb9q8qiLsFr0=vr0=vr0dc8meaabaqaciaacaGaaeqabaWaaeGaeaaakeaaimaacqWFse=uaaa@3845@. The support of the motif is the number of set bits in V
 MathType@MTEF@5@5@+=feaafiart1ev1aaatCvAUfKttLearuWrP9MDH5MBPbIqV92AaeXatLxBI9gBamrtHrhAL1wy0L2yHvtyaeHbnfgDOvwBHrxAJfwnaebbnrfifHhDYfgasaacH8akY=wiFfYdH8Gipec8Eeeu0xXdbba9frFj0=OqFfea0dXdd9vqai=hGuQ8kuc9pgc9s8qqaq=dirpe0xb9q8qiLsFr0=vr0=vr0dc8meaabaqaciaacaGaaeqabaWaaeGaeaaakeaaimaacqWFveVvaaa@384B@. Thus to obtain the support for a motif, we can simply union the bit vectors of all its (aggregate) neighbors. Using one bit to represent a sequence saves space, and also saves time via the union operation. However, since we need *n *fixed bits for each motif to store its bit vector, this is not efficient if there are many sequences, and if a motif occurs only in a small number of sequences, which leads to a sparse bit vector. Thus in the second method, EXMOTIF associates each motif with an identifier array, Q
 MathType@MTEF@5@5@+=feaafiart1ev1aaatCvAUfKttLearuWrP9MDH5MBPbIqV92AaeXatLxBI9gBamrtHrhAL1wy0L2yHvtyaeHbnfgDOvwBHrxAJfwnaebbnrfifHhDYfgasaacH8akY=wiFfYdH8Gipec8Eeeu0xXdbba9frFj0=OqFfea0dXdd9vqai=hGuQ8kuc9pgc9s8qqaq=dirpe0xb9q8qiLsFr0=vr0=vr0dc8meaabaqaciaacaGaaeqabaWaaeGaeaaakeaaimaacqWFqeFuaaa@3841@, to only store the sequence identifiers in which the motif occurs. EXMOTIF can then obtain the support for a motif by scanning the identifier arrays of its neighbors in linear time. For example consider again our motif (from Table 1), TAT[0,1]GG[2,3]CCAT, which occurs in *S*_2 _and *S*_3_, Its bit vector is thus V
 MathType@MTEF@5@5@+=feaafiart1ev1aaatCvAUfKttLearuWrP9MDH5MBPbIqV92AaeXatLxBI9gBamrtHrhAL1wy0L2yHvtyaeHbnfgDOvwBHrxAJfwnaebbnrfifHhDYfgasaacH8akY=wiFfYdH8Gipec8Eeeu0xXdbba9frFj0=OqFfea0dXdd9vqai=hGuQ8kuc9pgc9s8qqaq=dirpe0xb9q8qiLsFr0=vr0=vr0dc8meaabaqaciaacaGaaeqabaWaaeGaeaaakeaaimaacqWFveVvaaa@384B@ = {0110} and its identifier array Q
 MathType@MTEF@5@5@+=feaafiart1ev1aaatCvAUfKttLearuWrP9MDH5MBPbIqV92AaeXatLxBI9gBamrtHrhAL1wy0L2yHvtyaeHbnfgDOvwBHrxAJfwnaebbnrfifHhDYfgasaacH8akY=wiFfYdH8Gipec8Eeeu0xXdbba9frFj0=OqFfea0dXdd9vqai=hGuQ8kuc9pgc9s8qqaq=dirpe0xb9q8qiLsFr0=vr0=vr0dc8meaabaqaciaacaGaaeqabaWaaeGaeaaakeaaimaacqWFqeFuaaa@3841@ = {2, 3}.

##### Creating positional weight matrices

For any frequent structured motif ℳ
 MathType@MTEF@5@5@+=feaafiart1ev1aaatCvAUfKttLearuWrP9MDH5MBPbIqV92AaeXatLxBI9gBamrtHrhAL1wy0L2yHvtyaeHbnfgDOvwBHrxAJfwnaebbnrfifHhDYfgasaacH8akY=wiFfYdH8Gipec8Eeeu0xXdbba9frFj0=OqFfea0dXdd9vqai=hGuQ8kuc9pgc9s8qqaq=dirpe0xb9q8qiLsFr0=vr0=vr0dc8meaabaqaciaacaGaaeqabaWaaeGaeaaakeaaimaacqWFZestaaa@3790@, we can summarize the information about its neighbors (including ℳ
 MathType@MTEF@5@5@+=feaafiart1ev1aaatCvAUfKttLearuWrP9MDH5MBPbIqV92AaeXatLxBI9gBamrtHrhAL1wy0L2yHvtyaeHbnfgDOvwBHrxAJfwnaebbnrfifHhDYfgasaacH8akY=wiFfYdH8Gipec8Eeeu0xXdbba9frFj0=OqFfea0dXdd9vqai=hGuQ8kuc9pgc9s8qqaq=dirpe0xb9q8qiLsFr0=vr0=vr0dc8meaabaqaciaacaGaaeqabaWaaeGaeaaakeaaimaacqWFZestaaa@3790@) by computing a *Positional Weight Matrix *(PWM). The PWM for a structured motif ℳ
 MathType@MTEF@5@5@+=feaafiart1ev1aaatCvAUfKttLearuWrP9MDH5MBPbIqV92AaeXatLxBI9gBamrtHrhAL1wy0L2yHvtyaeHbnfgDOvwBHrxAJfwnaebbnrfifHhDYfgasaacH8akY=wiFfYdH8Gipec8Eeeu0xXdbba9frFj0=OqFfea0dXdd9vqai=hGuQ8kuc9pgc9s8qqaq=dirpe0xb9q8qiLsFr0=vr0=vr0dc8meaabaqaciaacaGaaeqabaWaaeGaeaaakeaaimaacqWFZestaaa@3790@ gives for each non-gap position the likelihood of occurrence for each symbol in Σ_DNA_. The PWM W
 MathType@MTEF@5@5@+=feaafiart1ev1aaatCvAUfKttLearuWrP9MDH5MBPbIqV92AaeXatLxBI9gBamrtHrhAL1wy0L2yHvtyaeHbnfgDOvwBHrxAJfwnaebbnrfifHhDYfgasaacH8akY=wiFfYdH8Gipec8Eeeu0xXdbba9frFj0=OqFfea0dXdd9vqai=hGuQ8kuc9pgc9s8qqaq=dirpe0xb9q8qiLsFr0=vr0=vr0dc8meaabaqaciaacaGaaeqabaWaaeGaeaaakeaaimaacqWFwe=vaaa@384D@ for ℳ
 MathType@MTEF@5@5@+=feaafiart1ev1aaatCvAUfKttLearuWrP9MDH5MBPbIqV92AaeXatLxBI9gBamrtHrhAL1wy0L2yHvtyaeHbnfgDOvwBHrxAJfwnaebbnrfifHhDYfgasaacH8akY=wiFfYdH8Gipec8Eeeu0xXdbba9frFj0=OqFfea0dXdd9vqai=hGuQ8kuc9pgc9s8qqaq=dirpe0xb9q8qiLsFr0=vr0=vr0dc8meaabaqaciaacaGaaeqabaWaaeGaeaaakeaaimaacqWFZestaaa@3790@ is calculated as follows:

rij=fij+pi∑k=1|ΣDNA|fkj+pk,Wij=ln⁡(rijpi)     (1)
 MathType@MTEF@5@5@+=feaafiart1ev1aaatCvAUfKttLearuWrP9MDH5MBPbIqV92AaeXatLxBI9gBamrtHrhAL1wy0L2yHvtyaeHbnfgDOvwBHrxAJfwnaebbnrfifHhDYfgasaacH8akY=wiFfYdH8Gipec8Eeeu0xXdbba9frFj0=OqFfea0dXdd9vqai=hGuQ8kuc9pgc9s8qqaq=dirpe0xb9q8qiLsFr0=vr0=vr0dc8meaabaqaciaacaGaaeqabaWaaeGaeaaakeaafaqabeqacaaabaGaemOCai3aaSbaaSqaaiabdMgaPjabdQgaQbqabaGccqGH9aqpdaWcaaqaaiabdAgaMnaaBaaaleaacqWGPbqAcqWGQbGAaeqaaOGaey4kaSIaemiCaa3aaSbaaSqaaiabdMgaPbqabaaakeaadaaeWaqaaiabdAgaMnaaBaaaleaacqWGRbWAcqWGQbGAaeqaaOGaey4kaSIaemiCaa3aaSbaaSqaaiabdUgaRbqabaaabaGaem4AaSMaeyypa0JaeGymaedabaWaaqWaaeaacqqHJoWudaWgaaadbaGaeeiraqKaeeOta4KaeeyqaeeabeaaaSGaay5bSlaawIa7aaqdcqGHris5aaaakiabcYcaSaqaaGWaaiab=zr8xnaaBaaaleaacqWGPbqAcqWGQbGAaeqaaOGaeyypa0JagiiBaWMaeiOBa42aaeWaaeaadaWcaaqaaiabdkhaYnaaBaaaleaacqWGPbqAcqWGQbGAaeqaaaGcbaGaemiCaa3aaSbaaSqaaiabdMgaPbqabaaaaaGccaGLOaGaayzkaaaaaiaaxMaacaWLjaWaaeWaaeaacqaIXaqmaiaawIcacaGLPaaaaaa@6FBB@

where, *f*_*ij *_and *r*_*ij *_represent the observed and relative frequency of symbol *i *at position *j*, respectively, *p*_*i *_is the prior probability of symbol *i*, and W
 MathType@MTEF@5@5@+=feaafiart1ev1aaatCvAUfKttLearuWrP9MDH5MBPbIqV92AaeXatLxBI9gBamrtHrhAL1wy0L2yHvtyaeHbnfgDOvwBHrxAJfwnaebbnrfifHhDYfgasaacH8akY=wiFfYdH8Gipec8Eeeu0xXdbba9frFj0=OqFfea0dXdd9vqai=hGuQ8kuc9pgc9s8qqaq=dirpe0xb9q8qiLsFr0=vr0=vr0dc8meaabaqaciaacaGaaeqabaWaaeGaeaaakeaaimaacqWFwe=vaaa@384D@_*ij *_is the weight (log-likelihood) of observing symbol *i *at position *j*. Whereas W
 MathType@MTEF@5@5@+=feaafiart1ev1aaatCvAUfKttLearuWrP9MDH5MBPbIqV92AaeXatLxBI9gBamrtHrhAL1wy0L2yHvtyaeHbnfgDOvwBHrxAJfwnaebbnrfifHhDYfgasaacH8akY=wiFfYdH8Gipec8Eeeu0xXdbba9frFj0=OqFfea0dXdd9vqai=hGuQ8kuc9pgc9s8qqaq=dirpe0xb9q8qiLsFr0=vr0=vr0dc8meaabaqaciaacaGaaeqabaWaaeGaeaaakeaaimaacqWFwe=vaaa@384D@ gives the likelihood of observing a given symbol in a given position in ℳ
 MathType@MTEF@5@5@+=feaafiart1ev1aaatCvAUfKttLearuWrP9MDH5MBPbIqV92AaeXatLxBI9gBamrtHrhAL1wy0L2yHvtyaeHbnfgDOvwBHrxAJfwnaebbnrfifHhDYfgasaacH8akY=wiFfYdH8Gipec8Eeeu0xXdbba9frFj0=OqFfea0dXdd9vqai=hGuQ8kuc9pgc9s8qqaq=dirpe0xb9q8qiLsFr0=vr0=vr0dc8meaabaqaciaacaGaaeqabaWaaeGaeaaakeaaimaacqWFZestaaa@3790@ it does not account for the degree to which some symbols are conserved at some positions. We can adjust the weights W
 MathType@MTEF@5@5@+=feaafiart1ev1aaatCvAUfKttLearuWrP9MDH5MBPbIqV92AaeXatLxBI9gBamrtHrhAL1wy0L2yHvtyaeHbnfgDOvwBHrxAJfwnaebbnrfifHhDYfgasaacH8akY=wiFfYdH8Gipec8Eeeu0xXdbba9frFj0=OqFfea0dXdd9vqai=hGuQ8kuc9pgc9s8qqaq=dirpe0xb9q8qiLsFr0=vr0=vr0dc8meaabaqaciaacaGaaeqabaWaaeGaeaaakeaaimaacqWFwe=vaaa@384D@_*ij *_by considering the information content at each position. The *information content *for a PWM is given as:

ℐij=rijln⁡(rij)−piln⁡(pi),ℐj=∑i=1|ΣDNA|ℐij,ℐW=∑j=1Kℐj     (2)
 MathType@MTEF@5@5@+=feaafiart1ev1aaatCvAUfKttLearuWrP9MDH5MBPbIqV92AaeXatLxBI9gBamrtHrhAL1wy0L2yHvtyaeHbnfgDOvwBHrxAJfwnaebbnrfifHhDYfgasaacH8akY=wiFfYdH8Gipec8Eeeu0xXdbba9frFj0=OqFfea0dXdd9vqai=hGuQ8kuc9pgc9s8qqaq=dirpe0xb9q8qiLsFr0=vr0=vr0dc8meaabaqaciaacaGaaeqabaWaaeGaeaaakeaafaqabeqadaaabaacdaGae8heHK0aaSbaaSqaaiabdMgaPjabdQgaQbqabaGccqGH9aqpcqWGYbGCdaWgaaWcbaGaemyAaKMaemOAaOgabeaakiGbcYgaSjabc6gaUjabcIcaOiabdkhaYnaaBaaaleaacqWGPbqAcqWGQbGAaeqaaOGaeiykaKIaeyOeI0IaemiCaa3aaSbaaSqaaiabdMgaPbqabaGccyGGSbaBcqGGUbGBcqGGOaakcqWGWbaCdaWgaaWcbaGaemyAaKgabeaakiabcMcaPiabcYcaSaqaaiab=brijnaaBaaaleaacqWGQbGAaeqaaOGaeyypa0ZaaabCaeaacqWFqessdaWgaaWcbaGaemyAaKMaemOAaOgabeaaaeaacqWGPbqAcqGH9aqpcqaIXaqmaeaadaabdaqaaiabfo6atnaaBaaameaacqqGebarcqqGobGtcqqGbbqqaeqaaaWccaGLhWUaayjcSdaaniabggHiLdGccqGGSaalaeaacqWFqessdaWgaaWcbaGae8NfXFfabeaakiabg2da9maaqahabaGae8heHK0aaSbaaSqaaiabdQgaQbqabaaabaGaemOAaOMaeyypa0JaeGymaedabaGaem4saSeaniabggHiLdaaaOGaaCzcaiaaxMaadaqadaqaaiabikdaYaGaayjkaiaawMcaaaaa@7BF1@

where *K *is the number of symbols in ℳ
 MathType@MTEF@5@5@+=feaafiart1ev1aaatCvAUfKttLearuWrP9MDH5MBPbIqV92AaeXatLxBI9gBamrtHrhAL1wy0L2yHvtyaeHbnfgDOvwBHrxAJfwnaebbnrfifHhDYfgasaacH8akY=wiFfYdH8Gipec8Eeeu0xXdbba9frFj0=OqFfea0dXdd9vqai=hGuQ8kuc9pgc9s8qqaq=dirpe0xb9q8qiLsFr0=vr0=vr0dc8meaabaqaciaacaGaaeqabaWaaeGaeaaakeaaimaacqWFZestaaa@3790@; ℐ
 MathType@MTEF@5@5@+=feaafiart1ev1aaatCvAUfKttLearuWrP9MDH5MBPbIqV92AaeXatLxBI9gBamrtHrhAL1wy0L2yHvtyaeHbnfgDOvwBHrxAJfwnaebbnrfifHhDYfgasaacH8akY=wiFfYdH8Gipec8Eeeu0xXdbba9frFj0=OqFfea0dXdd9vqai=hGuQ8kuc9pgc9s8qqaq=dirpe0xb9q8qiLsFr0=vr0=vr0dc8meaabaqaciaacaGaaeqabaWaaeGaeaaakeaaimaacqWFqessaaa@3769@_*ij *_is the information content of symbol *i *at position *j*; ℐ
 MathType@MTEF@5@5@+=feaafiart1ev1aaatCvAUfKttLearuWrP9MDH5MBPbIqV92AaeXatLxBI9gBamrtHrhAL1wy0L2yHvtyaeHbnfgDOvwBHrxAJfwnaebbnrfifHhDYfgasaacH8akY=wiFfYdH8Gipec8Eeeu0xXdbba9frFj0=OqFfea0dXdd9vqai=hGuQ8kuc9pgc9s8qqaq=dirpe0xb9q8qiLsFr0=vr0=vr0dc8meaabaqaciaacaGaaeqabaWaaeGaeaaakeaaimaacqWFqessaaa@3769@_*j *_is the information content over all bases at position *j*; and ℐW
 MathType@MTEF@5@5@+=feaafiart1ev1aaatCvAUfKttLearuWrP9MDH5MBPbIqV92AaeXatLxBI9gBamrtHrhAL1wy0L2yHvtyaeHbnfgDOvwBHrxAJfwnaebbnrfifHhDYfgasaacH8akY=wiFfYdH8Gipec8Eeeu0xXdbba9frFj0=OqFfea0dXdd9vqai=hGuQ8kuc9pgc9s8qqaq=dirpe0xb9q8qiLsFr0=vr0=vr0dc8meaabaqaciaacaGaaeqabaWaaeGaeaaakeaaimaacqWFqessdaWgaaWcbaGae8NfXFfabeaaaaa@3978@ is the information content of the entire matrix W
 MathType@MTEF@5@5@+=feaafiart1ev1aaatCvAUfKttLearuWrP9MDH5MBPbIqV92AaeXatLxBI9gBamrtHrhAL1wy0L2yHvtyaeHbnfgDOvwBHrxAJfwnaebbnrfifHhDYfgasaacH8akY=wiFfYdH8Gipec8Eeeu0xXdbba9frFj0=OqFfea0dXdd9vqai=hGuQ8kuc9pgc9s8qqaq=dirpe0xb9q8qiLsFr0=vr0=vr0dc8meaabaqaciaacaGaaeqabaWaaeGaeaaakeaaimaacqWFwe=vaaa@384D@. To allow mismatches at less conserved positions to be more easily tolerated than those at highly conserved positions, we multiply each W
 MathType@MTEF@5@5@+=feaafiart1ev1aaatCvAUfKttLearuWrP9MDH5MBPbIqV92AaeXatLxBI9gBamrtHrhAL1wy0L2yHvtyaeHbnfgDOvwBHrxAJfwnaebbnrfifHhDYfgasaacH8akY=wiFfYdH8Gipec8Eeeu0xXdbba9frFj0=OqFfea0dXdd9vqai=hGuQ8kuc9pgc9s8qqaq=dirpe0xb9q8qiLsFr0=vr0=vr0dc8meaabaqaciaacaGaaeqabaWaaeGaeaaakeaaimaacqWFwe=vaaa@384D@_*ij *_by ℐ
 MathType@MTEF@5@5@+=feaafiart1ev1aaatCvAUfKttLearuWrP9MDH5MBPbIqV92AaeXatLxBI9gBamrtHrhAL1wy0L2yHvtyaeHbnfgDOvwBHrxAJfwnaebbnrfifHhDYfgasaacH8akY=wiFfYdH8Gipec8Eeeu0xXdbba9frFj0=OqFfea0dXdd9vqai=hGuQ8kuc9pgc9s8qqaq=dirpe0xb9q8qiLsFr0=vr0=vr0dc8meaabaqaciaacaGaaeqabaWaaeGaeaaakeaaimaacqWFqessaaa@3769@_*j*_, which is larger for more conserved positions. As a result, the corrected weight of each element in the PWM W
 MathType@MTEF@5@5@+=feaafiart1ev1aaatCvAUfKttLearuWrP9MDH5MBPbIqV92AaeXatLxBI9gBamrtHrhAL1wy0L2yHvtyaeHbnfgDOvwBHrxAJfwnaebbnrfifHhDYfgasaacH8akY=wiFfYdH8Gipec8Eeeu0xXdbba9frFj0=OqFfea0dXdd9vqai=hGuQ8kuc9pgc9s8qqaq=dirpe0xb9q8qiLsFr0=vr0=vr0dc8meaabaqaciaacaGaaeqabaWaaeGaeaaakeaaimaacqWFwe=vaaa@384D@ becomes:

Wijc=ℐjln⁡(rijpi)     (3)
 MathType@MTEF@5@5@+=feaafiart1ev1aaatCvAUfKttLearuWrP9MDH5MBPbIqV92AaeXatLxBI9gBamrtHrhAL1wy0L2yHvtyaeHbnfgDOvwBHrxAJfwnaebbnrfifHhDYfgasaacH8akY=wiFfYdH8Gipec8Eeeu0xXdbba9frFj0=OqFfea0dXdd9vqai=hGuQ8kuc9pgc9s8qqaq=dirpe0xb9q8qiLsFr0=vr0=vr0dc8meaabaqaciaacaGaaeqabaWaaeGaeaaakeaaimaacqWFwe=vdaqhaaWcbaGaemyAaKMaemOAaOgabaGaem4yamgaaOGaeyypa0Jae8heHK0aaSbaaSqaaiabdQgaQbqabaGccyGGSbaBcqGGUbGBdaqadaqaamaalaaabaGaemOCai3aaSbaaSqaaiabdMgaPjabdQgaQbqabaaakeaacqWGWbaCdaWgaaWcbaGaemyAaKgabeaaaaaakiaawIcacaGLPaaacaWLjaGaaCzcamaabmaabaGaeG4mamdacaGLOaGaayzkaaaaaa@4F98@

Then we can calculate the PWM score, ℛ
 MathType@MTEF@5@5@+=feaafiart1ev1aaatCvAUfKttLearuWrP9MDH5MBPbIqV92AaeXatLxBI9gBamrtHrhAL1wy0L2yHvtyaeHbnfgDOvwBHrxAJfwnaebbnrfifHhDYfgasaacH8akY=wiFfYdH8Gipec8Eeeu0xXdbba9frFj0=OqFfea0dXdd9vqai=hGuQ8kuc9pgc9s8qqaq=dirpe0xb9q8qiLsFr0=vr0=vr0dc8meaabaqaciaacaGaaeqabaWaaeGaeaaakeaaimaacqWFBeIuaaa@377D@, for a structured motif, ℳ
 MathType@MTEF@5@5@+=feaafiart1ev1aaatCvAUfKttLearuWrP9MDH5MBPbIqV92AaeXatLxBI9gBamrtHrhAL1wy0L2yHvtyaeHbnfgDOvwBHrxAJfwnaebbnrfifHhDYfgasaacH8akY=wiFfYdH8Gipec8Eeeu0xXdbba9frFj0=OqFfea0dXdd9vqai=hGuQ8kuc9pgc9s8qqaq=dirpe0xb9q8qiLsFr0=vr0=vr0dc8meaabaqaciaacaGaaeqabaWaaeGaeaaakeaaimaacqWFZestaaa@3790@, by summing up the positional weights for the bases in ℳ
 MathType@MTEF@5@5@+=feaafiart1ev1aaatCvAUfKttLearuWrP9MDH5MBPbIqV92AaeXatLxBI9gBamrtHrhAL1wy0L2yHvtyaeHbnfgDOvwBHrxAJfwnaebbnrfifHhDYfgasaacH8akY=wiFfYdH8Gipec8Eeeu0xXdbba9frFj0=OqFfea0dXdd9vqai=hGuQ8kuc9pgc9s8qqaq=dirpe0xb9q8qiLsFr0=vr0=vr0dc8meaabaqaciaacaGaaeqabaWaaeGaeaaakeaaimaacqWFZestaaa@3790@, given as ℛ=∑j=1KWℳ[j]jc
 MathType@MTEF@5@5@+=feaafiart1ev1aaatCvAUfKttLearuWrP9MDH5MBPbIqV92AaeXatLxBI9gBamrtHrhAL1wy0L2yHvtyaeHbnfgDOvwBHrxAJfwnaebbnrfifHhDYfgasaacH8akY=wiFfYdH8Gipec8Eeeu0xXdbba9frFj0=OqFfea0dXdd9vqai=hGuQ8kuc9pgc9s8qqaq=dirpe0xb9q8qiLsFr0=vr0=vr0dc8meaabaqaciaacaGaaeqabaWaaeGaeaaakeaaimaacqWFBeIucqGH9aqpdaaeWaqaaiab=zr8xnaaDaaaleaacqWFZestcqGGBbWwcqWGQbGAcqGGDbqxcqWGQbGAaeaacqWGJbWyaaaabaGaemOAaOMaeyypa0JaeGymaedabaGaem4saSeaniabggHiLdaaaa@48AB@. Thus for each ℳ
 MathType@MTEF@5@5@+=feaafiart1ev1aaatCvAUfKttLearuWrP9MDH5MBPbIqV92AaeXatLxBI9gBamrtHrhAL1wy0L2yHvtyaeHbnfgDOvwBHrxAJfwnaebbnrfifHhDYfgasaacH8akY=wiFfYdH8Gipec8Eeeu0xXdbba9frFj0=OqFfea0dXdd9vqai=hGuQ8kuc9pgc9s8qqaq=dirpe0xb9q8qiLsFr0=vr0=vr0dc8meaabaqaciaacaGaaeqabaWaaeGaeaaakeaaimaacqWFZestaaa@3790@, its PWM score and PWM information content can be further used to measure whether ℳ
 MathType@MTEF@5@5@+=feaafiart1ev1aaatCvAUfKttLearuWrP9MDH5MBPbIqV92AaeXatLxBI9gBamrtHrhAL1wy0L2yHvtyaeHbnfgDOvwBHrxAJfwnaebbnrfifHhDYfgasaacH8akY=wiFfYdH8Gipec8Eeeu0xXdbba9frFj0=OqFfea0dXdd9vqai=hGuQ8kuc9pgc9s8qqaq=dirpe0xb9q8qiLsFr0=vr0=vr0dc8meaabaqaciaacaGaaeqabaWaaeGaeaaakeaaimaacqWFZestaaa@3790@ is a significant motif.

### Solving repeated structured motif identification problem

In repeated structured motif identification problem, the frequency closure property (that all the subsequences of a frequent sequence must be frequent), does not hold any more. For example, the sequence GCTTT, has three occurrences of pattern G[1,3]T, but it sub-pattern, G, has only one occurrence. Thus we cannot apply the closure property for pruning candidates. Nevertheless, a bound on the frequency of a sub-pattern can be established, which can be used for pruning.

**Theorem 1. ***Let *ℳ
 MathType@MTEF@5@5@+=feaafiart1ev1aaatCvAUfKttLearuWrP9MDH5MBPbIqV92AaeXatLxBI9gBamrtHrhAL1wy0L2yHvtyaeHbnfgDOvwBHrxAJfwnaebbnrfifHhDYfgasaacH8akY=wiFfYdH8Gipec8Eeeu0xXdbba9frFj0=OqFfea0dXdd9vqai=hGuQ8kuc9pgc9s8qqaq=dirpe0xb9q8qiLsFr0=vr0=vr0dc8meaabaqaciaacaGaaeqabaWaaeGaeaaakeaaimaacqWFZestaaa@3790@ = *M*_1 _... *M*_*k *_*be a structured motif and *ℳ′
 MathType@MTEF@5@5@+=feaafiart1ev1aaatCvAUfeBSjuyZL2yd9gzLbvyNv2CaerbwvMCKfMBHbqedmvETj2BSbWenfgDOvwBHrxAJfwnHbqeg0uy0HwzTfgDPnwy1aqee0evGueE0jxyaibaieIgFLIOYR2NHOxjYhrPYhrPYpI8F4rqqrFfpeea0xe9Lq=Jc9vqaqpepm0xbbG8FasPYRqj0=yi0lXdbba9pGe9qqFf0dXdHuk9fr=xfr=xfrpiWZqaaeaabiGaaiaacaqabeaadaqacqaaaOqaaGWaaiqb=ntinzaafaaaaa@406D@ = *M*_*i *_... *M*_*k *_*be a suffix of *ℳ
 MathType@MTEF@5@5@+=feaafiart1ev1aaatCvAUfKttLearuWrP9MDH5MBPbIqV92AaeXatLxBI9gBamrtHrhAL1wy0L2yHvtyaeHbnfgDOvwBHrxAJfwnaebbnrfifHhDYfgasaacH8akY=wiFfYdH8Gipec8Eeeu0xXdbba9frFj0=OqFfea0dXdd9vqai=hGuQ8kuc9pgc9s8qqaq=dirpe0xb9q8qiLsFr0=vr0=vr0dc8meaabaqaciaacaGaaeqabaWaaeGaeaaakeaaimaacqWFZestaaa@3790@, *for *1 ≤ *i *≤ *k*. *If the weighted support of *ℳ
 MathType@MTEF@5@5@+=feaafiart1ev1aaatCvAUfKttLearuWrP9MDH5MBPbIqV92AaeXatLxBI9gBamrtHrhAL1wy0L2yHvtyaeHbnfgDOvwBHrxAJfwnaebbnrfifHhDYfgasaacH8akY=wiFfYdH8Gipec8Eeeu0xXdbba9frFj0=OqFfea0dXdd9vqai=hGuQ8kuc9pgc9s8qqaq=dirpe0xb9q8qiLsFr0=vr0=vr0dc8meaabaqaciaacaGaaeqabaWaaeGaeaaakeaaimaacqWFZestaaa@3790@*is π*_*w *_(ℳ
 MathType@MTEF@5@5@+=feaafiart1ev1aaatCvAUfKttLearuWrP9MDH5MBPbIqV92AaeXatLxBI9gBamrtHrhAL1wy0L2yHvtyaeHbnfgDOvwBHrxAJfwnaebbnrfifHhDYfgasaacH8akY=wiFfYdH8Gipec8Eeeu0xXdbba9frFj0=OqFfea0dXdd9vqai=hGuQ8kuc9pgc9s8qqaq=dirpe0xb9q8qiLsFr0=vr0=vr0dc8meaabaqaciaacaGaaeqabaWaaeGaeaaakeaaimaacqWFZestaaa@3790@), *then *πw(ℳ′)≥πw(ℳ)∏m=1i−1Wm
MathType@MTEF@5@5@+=feaafiart1ev1aaatCvAUfKttLearuWrP9MDH5MBPbIqV92AaeXatLxBI9gBamrtHrhAL1wy0L2yHvtyaeHbnfgDOvwBHrxAJfwnaebbnrfifHhDYfgasaacH8akY=wiFfYdH8Gipec8Eeeu0xXdbba9frFj0=OqFfea0dXdd9vqai=hGuQ8kuc9pgc9s8qqaq=dirpe0xb9q8qiLsFr0=vr0=vr0dc8meaabaqaciaacaGaaeqabaWaaeGaeaaakeaaiiGacqWFapaCdaWgaaWcbaGaem4DaChabeaakiabcIcaOGWaaiqb+ntinzaafaGaeiykaKIaeyyzIm7aaSaaaeaacqWFapaCdaWgaaWcbaGaem4DaChabeaakiabcIcaOiab+ntinjabcMcaPaqaamaaradabaGaem4vaC1aaSbaaSqaaiabd2gaTbqabaaabaGaemyBa0Maeyypa0JaeGymaedabaGaemyAaKMaeyOeI0IaeGymaedaniabg+Givdaaaaaa@500D@, *where W*_*m *_= *u*_*m *_- *l*_*m *_+ 1 *is the span of the gap range for m *∈ [1, *k *- 1].

*Proof*. Let O
 MathType@MTEF@5@5@+=feaafiart1ev1aaatCvAUfKttLearuWrP9MDH5MBPbIqV92AaeXatLxBI9gBamrtHrhAL1wy0L2yHvtyaeHbnfgDOvwBHrxAJfwnaebbnrfifHhDYfgasaacH8akY=wiFfYdH8Gipec8Eeeu0xXdbba9frFj0=OqFfea0dXdd9vqai=hGuQ8kuc9pgc9s8qqaq=dirpe0xb9q8qiLsFr0=vr0=vr0dc8meaabaqaciaacaGaaeqabaWaaeGaeaaakeaaimaacqWFoe=taaa@383D@(ℳ
 MathType@MTEF@5@5@+=feaafiart1ev1aaatCvAUfKttLearuWrP9MDH5MBPbIqV92AaeXatLxBI9gBamrtHrhAL1wy0L2yHvtyaeHbnfgDOvwBHrxAJfwnaebbnrfifHhDYfgasaacH8akY=wiFfYdH8Gipec8Eeeu0xXdbba9frFj0=OqFfea0dXdd9vqai=hGuQ8kuc9pgc9s8qqaq=dirpe0xb9q8qiLsFr0=vr0=vr0dc8meaabaqaciaacaGaaeqabaWaaeGaeaaakeaaimaacqWFZestaaa@3790@) be the occurrence set of ℳ
 MathType@MTEF@5@5@+=feaafiart1ev1aaatCvAUfKttLearuWrP9MDH5MBPbIqV92AaeXatLxBI9gBamrtHrhAL1wy0L2yHvtyaeHbnfgDOvwBHrxAJfwnaebbnrfifHhDYfgasaacH8akY=wiFfYdH8Gipec8Eeeu0xXdbba9frFj0=OqFfea0dXdd9vqai=hGuQ8kuc9pgc9s8qqaq=dirpe0xb9q8qiLsFr0=vr0=vr0dc8meaabaqaciaacaGaaeqabaWaaeGaeaaakeaaimaacqWFZestaaa@3790@ and O
 MathType@MTEF@5@5@+=feaafiart1ev1aaatCvAUfKttLearuWrP9MDH5MBPbIqV92AaeXatLxBI9gBamrtHrhAL1wy0L2yHvtyaeHbnfgDOvwBHrxAJfwnaebbnrfifHhDYfgasaacH8akY=wiFfYdH8Gipec8Eeeu0xXdbba9frFj0=OqFfea0dXdd9vqai=hGuQ8kuc9pgc9s8qqaq=dirpe0xb9q8qiLsFr0=vr0=vr0dc8meaabaqaciaacaGaaeqabaWaaeGaeaaakeaaimaacqWFoe=taaa@383D@(ℳ′
 MathType@MTEF@5@5@+=feaafiart1ev1aaatCvAUfeBSjuyZL2yd9gzLbvyNv2CaerbwvMCKfMBHbqedmvETj2BSbWenfgDOvwBHrxAJfwnHbqeg0uy0HwzTfgDPnwy1aqee0evGueE0jxyaibaieIgFLIOYR2NHOxjYhrPYhrPYpI8F4rqqrFfpeea0xe9Lq=Jc9vqaqpepm0xbbG8FasPYRqj0=yi0lXdbba9pGe9qqFf0dXdHuk9fr=xfr=xfrpiWZqaaeaabiGaaiaacaqabeaadaqacqaaaOqaaGWaaiqb=ntinzaafaaaaa@406D@) be the occurrence set of ℳ′
 MathType@MTEF@5@5@+=feaafiart1ev1aaatCvAUfeBSjuyZL2yd9gzLbvyNv2CaerbwvMCKfMBHbqedmvETj2BSbWenfgDOvwBHrxAJfwnHbqeg0uy0HwzTfgDPnwy1aqee0evGueE0jxyaibaieIgFLIOYR2NHOxjYhrPYhrPYpI8F4rqqrFfpeea0xe9Lq=Jc9vqaqpepm0xbbG8FasPYRqj0=yi0lXdbba9pGe9qqFf0dXdHuk9fr=xfr=xfrpiWZqaaeaabiGaaiaacaqabeaadaqacqaaaOqaaGWaaiqb=ntinzaafaaaaa@406D@. For each occurrence of ℳ′
 MathType@MTEF@5@5@+=feaafiart1ev1aaatCvAUfeBSjuyZL2yd9gzLbvyNv2CaerbwvMCKfMBHbqedmvETj2BSbWenfgDOvwBHrxAJfwnHbqeg0uy0HwzTfgDPnwy1aqee0evGueE0jxyaibaieIgFLIOYR2NHOxjYhrPYhrPYpI8F4rqqrFfpeea0xe9Lq=Jc9vqaqpepm0xbbG8FasPYRqj0=yi0lXdbba9pGe9qqFf0dXdHuk9fr=xfr=xfrpiWZqaaeaabiGaaiaacaqabeaadaqacqaaaOqaaGWaaiqb=ntinzaafaaaaa@406D@ in O
 MathType@MTEF@5@5@+=feaafiart1ev1aaatCvAUfKttLearuWrP9MDH5MBPbIqV92AaeXatLxBI9gBamrtHrhAL1wy0L2yHvtyaeHbnfgDOvwBHrxAJfwnaebbnrfifHhDYfgasaacH8akY=wiFfYdH8Gipec8Eeeu0xXdbba9frFj0=OqFfea0dXdd9vqai=hGuQ8kuc9pgc9s8qqaq=dirpe0xb9q8qiLsFr0=vr0=vr0dc8meaabaqaciaacaGaaeqabaWaaeGaeaaakeaaimaacqWFoe=taaa@383D@(ℳ′
 MathType@MTEF@5@5@+=feaafiart1ev1aaatCvAUfeBSjuyZL2yd9gzLbvyNv2CaerbwvMCKfMBHbqedmvETj2BSbWenfgDOvwBHrxAJfwnHbqeg0uy0HwzTfgDPnwy1aqee0evGueE0jxyaibaieIgFLIOYR2NHOxjYhrPYhrPYpI8F4rqqrFfpeea0xe9Lq=Jc9vqaqpepm0xbbG8FasPYRqj0=yi0lXdbba9pGe9qqFf0dXdHuk9fr=xfr=xfrpiWZqaaeaabiGaaiaacaqabeaadaqacqaaaOqaaGWaaiqb=ntinzaafaaaaa@406D@), we can extend it to get occurrences of ℳ
 MathType@MTEF@5@5@+=feaafiart1ev1aaatCvAUfKttLearuWrP9MDH5MBPbIqV92AaeXatLxBI9gBamrtHrhAL1wy0L2yHvtyaeHbnfgDOvwBHrxAJfwnaebbnrfifHhDYfgasaacH8akY=wiFfYdH8Gipec8Eeeu0xXdbba9frFj0=OqFfea0dXdd9vqai=hGuQ8kuc9pgc9s8qqaq=dirpe0xb9q8qiLsFr0=vr0=vr0dc8meaabaqaciaacaGaaeqabaWaaeGaeaaakeaaimaacqWFZestaaa@3790@ in O
 MathType@MTEF@5@5@+=feaafiart1ev1aaatCvAUfKttLearuWrP9MDH5MBPbIqV92AaeXatLxBI9gBamrtHrhAL1wy0L2yHvtyaeHbnfgDOvwBHrxAJfwnaebbnrfifHhDYfgasaacH8akY=wiFfYdH8Gipec8Eeeu0xXdbba9frFj0=OqFfea0dXdd9vqai=hGuQ8kuc9pgc9s8qqaq=dirpe0xb9q8qiLsFr0=vr0=vr0dc8meaabaqaciaacaGaaeqabaWaaeGaeaaakeaaimaacqWFoe=taaa@383D@(ℳ
 MathType@MTEF@5@5@+=feaafiart1ev1aaatCvAUfKttLearuWrP9MDH5MBPbIqV92AaeXatLxBI9gBamrtHrhAL1wy0L2yHvtyaeHbnfgDOvwBHrxAJfwnaebbnrfifHhDYfgasaacH8akY=wiFfYdH8Gipec8Eeeu0xXdbba9frFj0=OqFfea0dXdd9vqai=hGuQ8kuc9pgc9s8qqaq=dirpe0xb9q8qiLsFr0=vr0=vr0dc8meaabaqaciaacaGaaeqabaWaaeGaeaaakeaaimaacqWFZestaaa@3790@) by adding *M*_1 _... *M*_*i*-1 _before ℳ′
 MathType@MTEF@5@5@+=feaafiart1ev1aaatCvAUfeBSjuyZL2yd9gzLbvyNv2CaerbwvMCKfMBHbqedmvETj2BSbWenfgDOvwBHrxAJfwnHbqeg0uy0HwzTfgDPnwy1aqee0evGueE0jxyaibaieIgFLIOYR2NHOxjYhrPYhrPYpI8F4rqqrFfpeea0xe9Lq=Jc9vqaqpepm0xbbG8FasPYRqj0=yi0lXdbba9pGe9qqFf0dXdHuk9fr=xfr=xfrpiWZqaaeaabiGaaiaacaqabeaadaqacqaaaOqaaGWaaiqb=ntinzaafaaaaa@406D@. This leads to at most ∏m=1i−1Wm
MathType@MTEF@5@5@+=feaafiart1ev1aaatCvAUfKttLearuWrP9MDH5MBPbIqV92AaeXatLxBI9gBaebbnrfifHhDYfgasaacH8akY=wiFfYdH8Gipec8Eeeu0xXdbba9frFj0=OqFfea0dXdd9vqai=hGuQ8kuc9pgc9s8qqaq=dirpe0xb9q8qiLsFr0=vr0=vr0dc8meaabaqaciaacaGaaeqabaqabeGadaaakeaadaqeWaqaaiabdEfaxnaaBaaaleaacqWGTbqBaeqaaaqaaiabd2gaTjabg2da9iabigdaXaqaaiabdMgaPjabgkHiTiabigdaXaqdcqGHpis1aaaa@37E9@ occurrences of ℳ
 MathType@MTEF@5@5@+=feaafiart1ev1aaatCvAUfKttLearuWrP9MDH5MBPbIqV92AaeXatLxBI9gBamrtHrhAL1wy0L2yHvtyaeHbnfgDOvwBHrxAJfwnaebbnrfifHhDYfgasaacH8akY=wiFfYdH8Gipec8Eeeu0xXdbba9frFj0=OqFfea0dXdd9vqai=hGuQ8kuc9pgc9s8qqaq=dirpe0xb9q8qiLsFr0=vr0=vr0dc8meaabaqaciaacaGaaeqabaWaaeGaeaaakeaaimaacqWFZestaaa@3790@ for any occurrence of ℳ′
 MathType@MTEF@5@5@+=feaafiart1ev1aaatCvAUfeBSjuyZL2yd9gzLbvyNv2CaerbwvMCKfMBHbqedmvETj2BSbWenfgDOvwBHrxAJfwnHbqeg0uy0HwzTfgDPnwy1aqee0evGueE0jxyaibaieIgFLIOYR2NHOxjYhrPYhrPYpI8F4rqqrFfpeea0xe9Lq=Jc9vqaqpepm0xbbG8FasPYRqj0=yi0lXdbba9pGe9qqFf0dXdHuk9fr=xfr=xfrpiWZqaaeaabiGaaiaacaqabeaadaqacqaaaOqaaGWaaiqb=ntinzaafaaaaa@406D@. Thus |O(ℳ′)|⋅∏m=1i−1Wm≥|O(ℳ)|
MathType@MTEF@5@5@+=feaafiart1ev1aaatCvAUfKttLearuWrP9MDH5MBPbIqV92AaeXatLxBI9gBamrtHrhAL1wy0L2yHvtyaeHbnfgDOvwBHrxAJfwnaebbnrfifHhDYfgasaacH8akY=wiFfYdH8Gipec8Eeeu0xXdbba9frFj0=OqFfea0dXdd9vqai=hGuQ8kuc9pgc9s8qqaq=dirpe0xb9q8qiLsFr0=vr0=vr0dc8meaabaqaciaacaGaaeqabaWaaeGaeaaakeaadaabdaqaaGWaaiab=5q8pjabcIcaOiqb=ntinzaafaGaeiykaKcacaGLhWUaayjcSdGaeyyXIC9aaebmaeaacqWGxbWvdaWgaaWcbaGaemyBa0gabeaaaeaacqWGTbqBcqGH9aqpcqaIXaqmaeaacqWGPbqAcqGHsislcqaIXaqma0Gaey4dIunakiabgwMiZoaaemaabaGae8NdX=KaeiikaGIae83mH0KaeiykaKcacaGLhWUaayjcSdaaaa@5567@, which immediately gives πw(ℳ′)≥πw(ℳ)∏m=1i−1Wm
MathType@MTEF@5@5@+=feaafiart1ev1aaatCvAUfKttLearuWrP9MDH5MBPbIqV92AaeXatLxBI9gBamrtHrhAL1wy0L2yHvtyaeHbnfgDOvwBHrxAJfwnaebbnrfifHhDYfgasaacH8akY=wiFfYdH8Gipec8Eeeu0xXdbba9frFj0=OqFfea0dXdd9vqai=hGuQ8kuc9pgc9s8qqaq=dirpe0xb9q8qiLsFr0=vr0=vr0dc8meaabaqaciaacaGaaeqabaWaaeGaeaaakeaaiiGacqWFapaCdaWgaaWcbaGaem4DaChabeaakiabcIcaOGWaaiqb+ntinzaafaGaeiykaKIaeyyzIm7aaSaaaeaacqWFapaCdaWgaaWcbaGaem4DaChabeaakiabcIcaOiab+ntinjabcMcaPaqaamaaradabaGaem4vaC1aaSbaaSqaaiabd2gaTbqabaaabaGaemyBa0Maeyypa0JaeGymaedabaGaemyAaKMaeyOeI0IaeGymaedaniabg+Givdaaaaaa@500D@.     □

With Theorem 1, EXMOTIF can calculate a support bound for any suffix ℳ′
 MathType@MTEF@5@5@+=feaafiart1ev1aaatCvAUfeBSjuyZL2yd9gzLbvyNv2CaerbwvMCKfMBHbqedmvETj2BSbWenfgDOvwBHrxAJfwnHbqeg0uy0HwzTfgDPnwy1aqee0evGueE0jxyaibaieIgFLIOYR2NHOxjYhrPYhrPYpI8F4rqqrFfpeea0xe9Lq=Jc9vqaqpepm0xbbG8FasPYRqj0=yi0lXdbba9pGe9qqFf0dXdHuk9fr=xfr=xfrpiWZqaaeaabiGaaiaacaqabeaadaqacqaaaOqaaGWaaiqb=ntinzaafaaaaa@406D@ of ℳ
 MathType@MTEF@5@5@+=feaafiart1ev1aaatCvAUfKttLearuWrP9MDH5MBPbIqV92AaeXatLxBI9gBamrtHrhAL1wy0L2yHvtyaeHbnfgDOvwBHrxAJfwnaebbnrfifHhDYfgasaacH8akY=wiFfYdH8Gipec8Eeeu0xXdbba9frFj0=OqFfea0dXdd9vqai=hGuQ8kuc9pgc9s8qqaq=dirpe0xb9q8qiLsFr0=vr0=vr0dc8meaabaqaciaacaGaaeqabaWaaeGaeaaakeaaimaacqWFZestaaa@3790@, given the quorum requirement *q*. For example, assume that the motif template is NN[3,5]NNN[0,4]NNN and *q *= 100, with *W*_1 _= 5 - 3 + 1 = 3 and *W*_2 _= 4 - 0 + 1 = 5. When processing the suffix component ℳ′
 MathType@MTEF@5@5@+=feaafiart1ev1aaatCvAUfeBSjuyZL2yd9gzLbvyNv2CaerbwvMCKfMBHbqedmvETj2BSbWenfgDOvwBHrxAJfwnHbqeg0uy0HwzTfgDPnwy1aqee0evGueE0jxyaibaieIgFLIOYR2NHOxjYhrPYhrPYpI8F4rqqrFfpeea0xe9Lq=Jc9vqaqpepm0xbbG8FasPYRqj0=yi0lXdbba9pGe9qqFf0dXdHuk9fr=xfr=xfrpiWZqaaeaabiGaaiaacaqabeaadaqacqaaaOqaaGWaaiqb=ntinzaafaaaaa@406D@ = NNN, we require that *π*_*w*_(ℳ′
 MathType@MTEF@5@5@+=feaafiart1ev1aaatCvAUfeBSjuyZL2yd9gzLbvyNv2CaerbwvMCKfMBHbqedmvETj2BSbWenfgDOvwBHrxAJfwnHbqeg0uy0HwzTfgDPnwy1aqee0evGueE0jxyaibaieIgFLIOYR2NHOxjYhrPYhrPYpI8F4rqqrFfpeea0xe9Lq=Jc9vqaqpepm0xbbG8FasPYRqj0=yi0lXdbba9pGe9qqFf0dXdHuk9fr=xfr=xfrpiWZqaaeaabiGaaiaacaqabeaadaqacqaaaOqaaGWaaiqb=ntinzaafaaaaa@406D@) ≥ 1003×5
 MathType@MTEF@5@5@+=feaafiart1ev1aaatCvAUfKttLearuWrP9MDH5MBPbIqV92AaeXatLxBI9gBaebbnrfifHhDYfgasaacH8akY=wiFfYdH8Gipec8Eeeu0xXdbba9frFj0=OqFfea0dXdd9vqai=hGuQ8kuc9pgc9s8qqaq=dirpe0xb9q8qiLsFr0=vr0=vr0dc8meaabaqaciaacaGaaeqabaqabeGadaaakeaadaWcaaqaaiabigdaXiabicdaWiabicdaWaqaaiabiodaZiabgEna0kabiwda1aaaaaa@338B@ = 6; when processing ℳ′
 MathType@MTEF@5@5@+=feaafiart1ev1aaatCvAUfeBSjuyZL2yd9gzLbvyNv2CaerbwvMCKfMBHbqedmvETj2BSbWenfgDOvwBHrxAJfwnHbqeg0uy0HwzTfgDPnwy1aqee0evGueE0jxyaibaieIgFLIOYR2NHOxjYhrPYhrPYpI8F4rqqrFfpeea0xe9Lq=Jc9vqaqpepm0xbbG8FasPYRqj0=yi0lXdbba9pGe9qqFf0dXdHuk9fr=xfr=xfrpiWZqaaeaabiGaaiaacaqabeaadaqacqaaaOqaaGWaaiqb=ntinzaafaaaaa@406D@ = NNN[0,4]NNN, we require that *π*_*w*_(ℳ′
 MathType@MTEF@5@5@+=feaafiart1ev1aaatCvAUfeBSjuyZL2yd9gzLbvyNv2CaerbwvMCKfMBHbqedmvETj2BSbWenfgDOvwBHrxAJfwnHbqeg0uy0HwzTfgDPnwy1aqee0evGueE0jxyaibaieIgFLIOYR2NHOxjYhrPYhrPYpI8F4rqqrFfpeea0xe9Lq=Jc9vqaqpepm0xbbG8FasPYRqj0=yi0lXdbba9pGe9qqFf0dXdHuk9fr=xfr=xfrpiWZqaaeaabiGaaiaacaqabeaadaqacqaaaOqaaGWaaiqb=ntinzaafaaaaa@406D@) ≥ 1003
 MathType@MTEF@5@5@+=feaafiart1ev1aaatCvAUfKttLearuWrP9MDH5MBPbIqV92AaeXatLxBI9gBaebbnrfifHhDYfgasaacH8akY=wiFfYdH8Gipec8Eeeu0xXdbba9frFj0=OqFfea0dXdd9vqai=hGuQ8kuc9pgc9s8qqaq=dirpe0xb9q8qiLsFr0=vr0=vr0dc8meaabaqaciaacaGaaeqabaqabeGadaaakeaadaWcaaqaaiabigdaXiabicdaWiabicdaWaqaaiabiodaZaaaaaa@307C@ = 33. Thus even the weaker bounds can lead to some pruning.

### The complete EXMOTIF algorithm: complexity analysis

**Figure 6:**
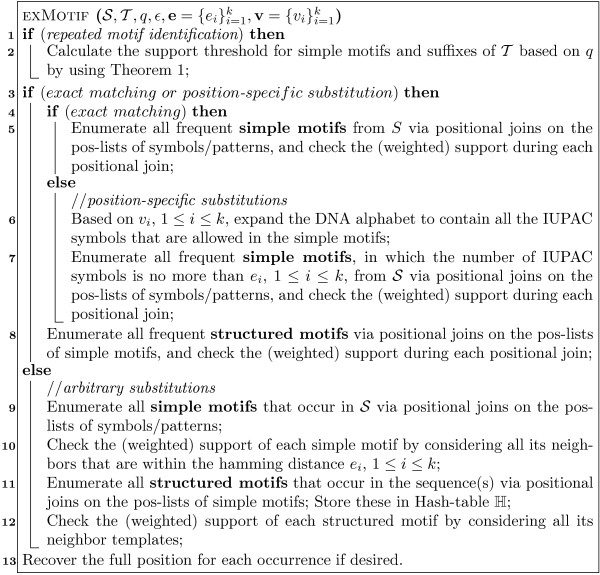
EXMOTIF Algorithm.

The pseudo-code for the complete EXMOTIF algorithm is shown in Figure 6. The program takes as inputs the set of sequences S={Si}i=1n
 MathType@MTEF@5@5@+=feaafiart1ev1aaatCvAUfKttLearuWrP9MDH5MBPbIqV92AaeXatLxBI9gBamrtHrhAL1wy0L2yHvtyaeHbnfgDOvwBHrxAJfwnaebbnrfifHhDYfgasaacH8akY=wiFfYdH8Gipec8Eeeu0xXdbba9frFj0=OqFfea0dXdd9vqai=hGuQ8kuc9pgc9s8qqaq=dirpe0xb9q8qiLsFr0=vr0=vr0dc8meaabaqaciaacaGaaeqabaWaaeGaeaaakeaaimaacqWFse=ucqGH9aqpcqGG7bWEcqWGtbWudaWgaaWcbaGaemyAaKgabeaakiabc2ha9naaDaaaleaacqWGPbqAcqGH9aqpcqaIXaqmaeaacqWGUbGBaaaaaa@43EE@, the motif template T
 MathType@MTEF@5@5@+=feaafiart1ev1aaatCvAUfKttLearuWrP9MDH5MBPbIqV92AaeXatLxBI9gBamrtHrhAL1wy0L2yHvtyaeHbnfgDOvwBHrxAJfwnaebbnrfifHhDYfgasaacH8akY=wiFfYdH8Gipec8Eeeu0xXdbba9frFj0=OqFfea0dXdd9vqai=hGuQ8kuc9pgc9s8qqaq=dirpe0xb9q8qiLsFr0=vr0=vr0dc8meaabaqaciaacaGaaeqabaWaaeGaeaaakeaaimaacqWFtepvaaa@3847@ = *M*_1_[*l*_1_, *u*_1_] ... [*l*_*k*-1_, *u*_*k*-1_] *M*_*k*_, the quorum threshold *q*, the number of errors or IUPAC symbols allowed per simple motif e={ei}i=1k
 MathType@MTEF@5@5@+=feaafiart1ev1aaatCvAUfKttLearuWrP9MDH5MBPbIqV92AaeXatLxBI9gBaebbnrfifHhDYfgasaacH8akY=wiFfYdH8Gipec8Eeeu0xXdbba9frFj0=OqFfea0dXdd9vqai=hGuQ8kuc9pgc9s8qqaq=dirpe0xb9q8qiLsFr0=vr0=vr0dc8meaabaqaciaacaGaaeqabaqabeGadaaakeaaieqacqWFLbqzcqGH9aqpcqGG7bWEcqWGLbqzdaWgaaWcbaGaemyAaKgabeaakiabc2ha9naaDaaaleaacqWGPbqAcqGH9aqpcqaIXaqmaeaacqWGRbWAaaaaaa@39CC@, and the set of IUPAC symbols to use per simple motif, v={vi}i=1k
 MathType@MTEF@5@5@+=feaafiart1ev1aaatCvAUfKttLearuWrP9MDH5MBPbIqV92AaeXatLxBI9gBaebbnrfifHhDYfgasaacH8akY=wiFfYdH8Gipec8Eeeu0xXdbba9frFj0=OqFfea0dXdd9vqai=hGuQ8kuc9pgc9s8qqaq=dirpe0xb9q8qiLsFr0=vr0=vr0dc8meaabaqaciaacaGaaeqabaqabeGadaaakeaaieqacqWF2bGDcqGH9aqpcqGG7bWEcqWG2bGDdaWgaaWcbaGaemyAaKgabeaakiabc2ha9naaDaaaleaacqWGPbqAcqGH9aqpcqaIXaqmaeaacqWGRbWAaaaaaa@3A10@ (only for position specific substitutions). As outlined in Figure 6 EXMOTIF allows several different variations to motif extraction, as described above. These variations include, exact matching, position-specific substitutions via use of IUPAC symbols, arbitrary substitutions, and repeated motif identification.

EXMOTIF initially adjusts the support thresholds if the task is repeated motif identification (lines 1–2). The main approach for handling exact matches or position-specific substitutions is the same. The main difference is that while enumerating the simple motifs, EXMOTIF uses the appropriate IUPAC alphabet (specified by *v*_*i *_for component *M*_*i*_; lines 6–7). The structured motifs are found via positional joins over the simple motifs (line 8). The positional joins are performed as described in Figure 1. For arbitrary substitutions, EXMOTIF first enumerates the simple motifs (line 9) and checks their aggregate support (i.e., including the supports of all neighbors within error *ε*_*i*_). From these, the structured motifs are enumerated and stored in a hash-table (ℍ; line 11). Lastly, the aggregate support of all these motifs is computed as described in Figure 5 (line 12). Those that meet the quorum will be output. Finally, if desired, EXMOTIF recovers the full positions for each occurrence, via the procedure outlined in Figure 2.

In terms of the computational complexity of EXMOTIF, let's first consider the complexity of extracting the simple motifs. Assume that *m *is the length of the longest simple motif component in the structured template T
 MathType@MTEF@5@5@+=feaafiart1ev1aaatCvAUfKttLearuWrP9MDH5MBPbIqV92AaeXatLxBI9gBamrtHrhAL1wy0L2yHvtyaeHbnfgDOvwBHrxAJfwnaebbnrfifHhDYfgasaacH8akY=wiFfYdH8Gipec8Eeeu0xXdbba9frFj0=OqFfea0dXdd9vqai=hGuQ8kuc9pgc9s8qqaq=dirpe0xb9q8qiLsFr0=vr0=vr0dc8meaabaqaciaacaGaaeqabaWaaeGaeaaakeaaimaacqWFtepvaaa@3847@. Note that there are potentially |Σ|^*m *^frequent simple motifs at that length, but due to the quorum requirement, many of these will not be frequent. Nevertheless, in the worst case *O*(|Σ|^*m*^) simple components may be extracted. For a simple motif of length *m*, EXMOTIF uses *O*(log(*m*)) positional joins to obtain its support, and each such join takes *O*(*N*) time, where N=∑i=1n|Si|
 MathType@MTEF@5@5@+=feaafiart1ev1aaatCvAUfKttLearuWrP9MDH5MBPbIqV92AaeXatLxBI9gBaebbnrfifHhDYfgasaacH8akY=wiFfYdH8Gipec8Eeeu0xXdbba9frFj0=OqFfea0dXdd9vqai=hGuQ8kuc9pgc9s8qqaq=dirpe0xb9q8qiLsFr0=vr0=vr0dc8meaabaqaciaacaGaaeqabaqabeGadaaakeaacqWGobGtcqGH9aqpdaaeWaqaamaaemaabaGaem4uam1aaSbaaSqaaiabdMgaPbqabaaakiaawEa7caGLiWoaaSqaaiabdMgaPjabg2da9iabigdaXaqaaiabd6gaUbqdcqGHris5aaaa@3B71@ is the sum of the lengths of all the sequences *S*_*i *_in the database S
 MathType@MTEF@5@5@+=feaafiart1ev1aaatCvAUfKttLearuWrP9MDH5MBPbIqV92AaeXatLxBI9gBamrtHrhAL1wy0L2yHvtyaeHbnfgDOvwBHrxAJfwnaebbnrfifHhDYfgasaacH8akY=wiFfYdH8Gipec8Eeeu0xXdbba9frFj0=OqFfea0dXdd9vqai=hGuQ8kuc9pgc9s8qqaq=dirpe0xb9q8qiLsFr0=vr0=vr0dc8meaabaqaciaacaGaaeqabaWaaeGaeaaakeaaimaacqWFse=uaaa@3845@. Thus, extracting the simple motifs takes time *O*(*N *log(*m*)|Σ|^*m*^) in the worst case.

With |Σ|^*m *^simple motifs, there are *O*(|Σ|^*mk*^) potential structured motifs, though a vast majority of these will not meet the quorum requirement. Extracting the structured motifs then takes time *O*(*kN*|Σ|^*mk*^) for the exact match and position-specific substitution cases. For arbitrary substitutions there is additional cost of enumerating aggregate neighbors and computing their support. For each motif we have to consider ∏i=1k(|Mi|εi)
MathType@MTEF@5@5@+=feaafiart1ev1aaatCvAUfKttLearuWrP9MDH5MBPbIqV92AaeXatLxBI9gBaebbnrfifHhDYfgasaacH8akY=wiFfYdH8Gipec8Eeeu0xXdbba9frFj0=OqFfea0dXdd9vqai=hGuQ8kuc9pgc9s8qqaq=dirpe0xb9q8qiLsFr0=vr0=vr0dc8meaabaqaciaacaGaaeqabaqabeGadaaakeaadaqeWaqaamaabmaabaqbaeqabiqaaaqaamaaemaabaGaemyta00aaSbaaSqaaiabdMgaPbqabaaakiaawEa7caGLiWoaaeaaiiGacqWF1oqzdaWgaaWcbaGaemyAaKgabeaaaaaakiaawIcacaGLPaaaaSqaaiabdMgaPjabg2da9iabigdaXaqaaiabdUgaRbqdcqGHpis1aaaa@3DF8@ = *km*^*e *^aggregate neighbors, where *e *= max_*i*_{*e*_*i*_}. Furthermore, an aggregate neighbor can have *k*|Σ|^*e *^matching motifs. Thus the time complexity of extracting all the structured motifs is *O*(*kN*|Σ|^*mk *^+ *k*^2^*m*^*e*^|Σ|^*e*^) for arbitrary substitutions. Since typically *mk *> *e *and *N *> *m*^*e*^, the time complexity is essentially *O*(*kN*|Σ|^*mk*^). Combined with the cost for simple motif extraction, the computational complexity of EXMOTIF is then given as *O*(log(*m*) *N *|Σ|^*m *^+ *kN*|Σ|^*km*^) = *O*(*kN*|Σ|^*km*^).

## Experimental results

EXMOTIF has been implemented in C++, and compiled with g++ v4.0.0 at optimization level 3 (-O3). We performed experiments on a Macintosh PowerPC G5 with dual 2.7GHz processors and 4GB memory running Mac OS X vl0.4.5. We compare our results with the latest version of RISO [15-17] (called RISOTTO [17]), the best previous algorithm for structured motif extraction problem.

### EXMOTIF and RISO: comparison

**Figure 7:**
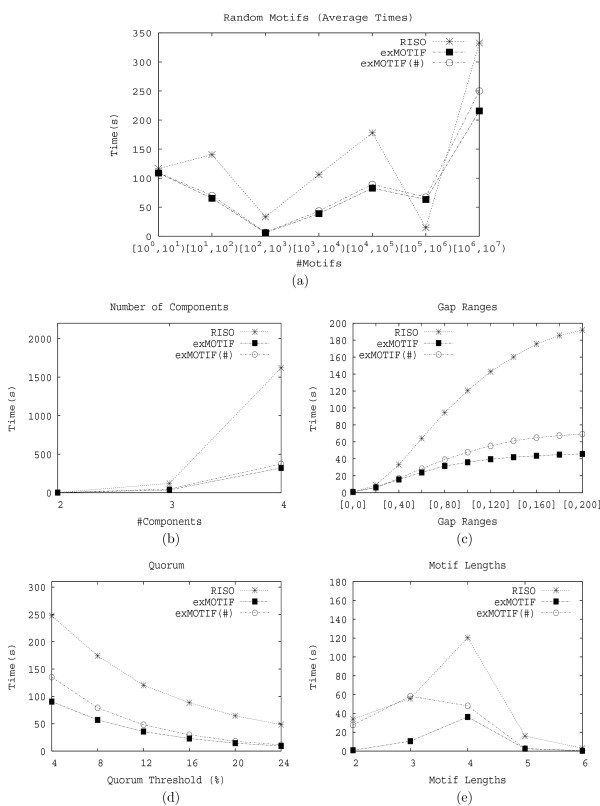
EXMOTIF vs. RISO: Exact Matching.

**Figure 8:**
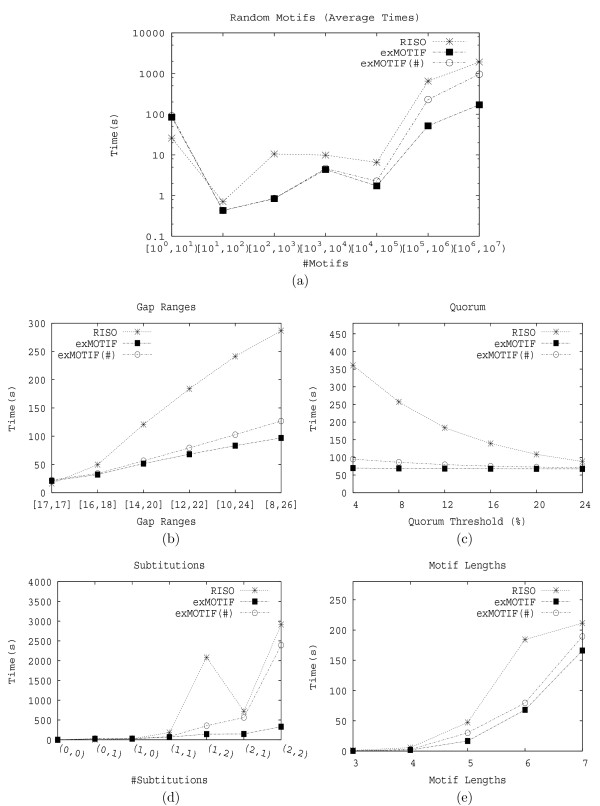
EXMOTIF vs. RISO: Approximate Matching.

For comparison, we extract structured motifs from 1,062 non-coding sequences (a total of 196,736 nucleotides) located between two divergent genes in the genome of *B. subtilis *[15-17]. Figure 7 and 8 compare the running time (in seconds) for EXMOTIF and RISO using exact matching and approximate matching, respectively. Experiments were done for different gap ranges, number of components, and quorum thresholds. Note that EXMOTIF has two options: one (shown as "exMOTIF" in the figures) for reporting only the number of sequences where the structured motifs occur, the other (shown as "exMOTIF(#)") for reporting both the number of sequences where the structured motifs occur and the actual occurrences. Also note that the current implementation of RISO *does not *report the actual occurrences; it reports only the frequency.

#### Exact matching

In the first experiment, shown in Figure 7(a), we randomly generated 100 structured motif templates, with *k *∈ [2,4] simple motifs of length *l *∈ [4,7] (*k *and *l *are selected uniformly at random within the given ranges). The gap range between each pair of simple motifs is a random sub-interval of [0, 200]. The x-axis is sorted on the number of motifs extracted. For clarity we plot average times for the methods when the number of motifs extracted fall into the given range on the x-axis. For example, the time plotted for the range [10^2^, 10^3^) is the average time for all the random templates that produce between 100 and 1000 motifs. We find that the average running time for RISO across all extracted motifs is 120.7s, whereas for EXMOTIF it takes 88.4s for reporting only the supports, and 91.3s for also reporting all the occurrences. The median times were 26.3s, 8.5s, and 9.2s, respectively, indicating a 3 times speed-up of EXMOTIF over RISO.

In the next set of experiments we varied one parameter while keeping the others fixed. We set the default quorum to 12% (*q *= 127), the default gap ranges to [0,100], the default simple motif length to *l *= 4 (NNNN), and the default number of components *k *= 3 (e.g., NNNN[0,100]NNNN[0,100]NNNN). In Figure 7(b), we plot the time as a function of the number of simple motifs *k *in the template. We find that as the number of components increases the time gap between EXMOTIF and RISO increases; for *k *= 4 simple motifs, EXMOTIF is around 5 times faster than RISO. Figure 7(c) shows the effect of increasing gap ranges, from [0,0] to [0,200]. We find that as the gap range increases the time for EXMOTIF increases at a slower rate compared to RISO. For [0,200], EXMOTIF is 3–4 times faster than RISO depending whether only frequency or full occurrences are reported. In Figure 7(d), as the quorum threshold increases, the running time goes down for both methods. For quorum 24%, EXMOTIF is 4–5 times faster than RISO. As support decreases, the gap narrows somewhat, but EXMOTIF remains 2–3 times faster. Finally, Figure 7(e) plots the effect of increasing simple motif lengths *l *∈ [2,6]. We find that the time first increases and then decreases. This is because there are a large number of motif occurrences for length 3 and length 4, but relatively few occurrences for length 5 and length 6. Depending on the motif lengths, EXMOTIF can be 3–40 times faster than RISO for comparable output, i.e., reporting only the support. EXMOTIF remains up to 5 times faster when also reporting the actual occurrences.

To compare the performance for extracting structured motifs with length ranges, we used the template T
 MathType@MTEF@5@5@+=feaafiart1ev1aaatCvAUfKttLearuWrP9MDH5MBPbIqV92AaeXatLxBI9gBamrtHrhAL1wy0L2yHvtyaeHbnfgDOvwBHrxAJfwnaebbnrfifHhDYfgasaacH8akY=wiFfYdH8Gipec8Eeeu0xXdbba9frFj0=OqFfea0dXdd9vqai=hGuQ8kuc9pgc9s8qqaq=dirpe0xb9q8qiLsFr0=vr0=vr0dc8meaabaqaciaacaGaaeqabaWaaeGaeaaakeaaimaacqWFtepvaaa@3847@ = *M*_1_[50, 100] *M*_2_[1,50]*M*_3_[20, 100]*M*_4 _with *q *= 12%, where |*M*_1_| ∈ [2,4], |*M*_2_| ∈ [3,4], |*M*_3_| ∈ [5,6], |*M*_4_| ∈ [4,5]. EXMOTIF took 78.4s, whereas RISO took 1640.9s to extract 14,174 motifs.

#### Approximate matching

In the first experiment, shown in Figure 8(a), we randomly generated 30 structured motif templates, with *k *∈ [2,3] simple motifs of length *l *∈ [3,6] (*k *and *l *are selected uniformly at random within the given ranges). The gap range between each pair of simple motifs is a random sub-interval of [10, 30]. The x-axis is sorted on the number of motifs extracted, and average times are plotted for the extracted number of motifs in the given range. We find that the average running time for RISO is 334.5s, whereas for EXMOTIF it takes 59.3s seconds for reporting only the support, and 176.7s for also reporting all the occurrences. Thus EXMOTIF is on average 5 times faster than RISO, with comparable output.

Figures 8(b)–(e) plot the time for approximate matching as a function of different parameters. We set the default quorum to 12% (*q *= 127, out of |S
 MathType@MTEF@5@5@+=feaafiart1ev1aaatCvAUfKttLearuWrP9MDH5MBPbIqV92AaeXatLxBI9gBamrtHrhAL1wy0L2yHvtyaeHbnfgDOvwBHrxAJfwnaebbnrfifHhDYfgasaacH8akY=wiFfYdH8Gipec8Eeeu0xXdbba9frFj0=OqFfea0dXdd9vqai=hGuQ8kuc9pgc9s8qqaq=dirpe0xb9q8qiLsFr0=vr0=vr0dc8meaabaqaciaacaGaaeqabaWaaeGaeaaakeaaimaacqWFse=uaaa@3845@| = 1062 sequences), the default gap ranges to [12,22], the default simple motif length to *l *= 6 (NNNNNN), and the default number of components *k *= 2 (e.g., NNNNNN[12,22]NNNNNN). Figure 8(b) shows how increasing gap ranges effect the running time; for gap range [8,26] between the two motif components, EXMOTIF is 2–3 times faster than RISO. In Figure 8(c), we increase the numbers of arbitrary substitutions allowed for each simple motif; a pair (*ε*_1_, *ε*_2_) on the x-axis denotes that *ε*_1 _substitutions are allowed for motif component *M*_1_, and *ε*_2 _for *M*_2_. We can see that EXMOTIF is always faster than RISO. It is 9 times faster when only frequencies are reported, and it can be up to 5 times faster then full occurrences are reported, though for some cases the difference is slight.

Figure 8(d) plots the effect of the quorum threshold. Compared to RISO, EXMOTIF performs much better for low quorum, e.g., for *q *= 4% EXMOTIF is 4–5 times faster than RISO. Finally in Figure 8(e), as the simple motif lengths increase, the time for both EXMOTIF and RISO increases, and we find that EXMOTIF can be 2–3 times faster.

We also studied the effect of quorum and allowed substitutions. Table 4 shows the comparative results for EXMOTIF and RISO. Here we used the template T
 MathType@MTEF@5@5@+=feaafiart1ev1aaatCvAUfKttLearuWrP9MDH5MBPbIqV92AaeXatLxBI9gBamrtHrhAL1wy0L2yHvtyaeHbnfgDOvwBHrxAJfwnaebbnrfifHhDYfgasaacH8akY=wiFfYdH8Gipec8Eeeu0xXdbba9frFj0=OqFfea0dXdd9vqai=hGuQ8kuc9pgc9s8qqaq=dirpe0xb9q8qiLsFr0=vr0=vr0dc8meaabaqaciaacaGaaeqabaWaaeGaeaaakeaaimaacqWFtepvaaa@3847@ = NNNNNN[12, 22]NNNNNN to extract motifs from the 1062 subsequences from *B. subtilis*. We vary the quorum from low (5%) to high (90%), and vary the number of errors *e*_*i *_per simple motif (with more errors allowed for higher quorum). For a comparable output (when only the frequency is reported), EXMOTIF outperforms RISO, especially for high quorum and high number of errors. It is interesting that for this latter case, reporting all occurrences incurs significant overhead. For example for *q *= 90% and with (*e*_1 _= 3, *e*_2 _= 3), EXMOTIF is 20 times faster than RISO, but EXMOTIF(#) is 3 times slower!

**Table 4:** Comparison of EXMOTIF and RISO for different quorums and allowed substitutions.

Quorum	#Substitutions	RISO	EXMOTIF	EXMOTIF(#)
5%	(0, 0)	1.82s	1.42s	1.52s
30%	(1, 1)	63.01s	58.91s	64.52s
60%	(2, 2)	2763.31s	328.43s	2317.35s
90%	(3, 3)	13682.13s	707.56s	41464.93s

The template used is T
 MathType@MTEF@5@5@+=feaafiart1ev1aaatCvAUfKttLearuWrP9MDH5MBPbIqV92AaeXatLxBI9gBamrtHrhAL1wy0L2yHvtyaeHbnfgDOvwBHrxAJfwnaebbnrfifHhDYfgasaacH8akY=wiFfYdH8Gipec8Eeeu0xXdbba9frFj0=OqFfea0dXdd9vqai=hGuQ8kuc9pgc9s8qqaq=dirpe0xb9q8qiLsFr0=vr0=vr0dc8meaabaqaciaacaGaaeqabaWaaeGaeaaakeaaimaacqWFtepvaaa@3847@ = *NNNNNN*[12,22]*NNNNNN*. #Substitutions shows the number of errors (*e*_1_, *e*_2_) allowed for the two simple components.

### Real applications

#### Discovery of single transcription factor binding sites

We evaluate our algorithm by extracting the conserved features of known transcription factor binding sites in yeast. In particular we used the binding sites for the Zinc (Zn) factors [11]. There are 11 binding sites listed for the Zn cluster, 3 of which are simple motifs. The remaining 8 are structured, as shown in Table 5. For the evaluation, we first form several structured motif templates according to the conserved features in the binding sites. Then we extract the frequent structured motifs satisfying these templates from the upstream regions of 68 genes regulated by zinc factors [11]. We used the -1000 to -1 upstream regions, truncating the region if and where it overlaps with an upstream open-reading frame (ORF). After extraction, since binding sites cannot have many occurrences in the ORF regions, we drop some motifs if they also occur frequently in the ORF regions (i.e., within the genes). Finally, we calculate the Z-scores for the remaining frequent motifs, and rank them by descending Z-scores. In our experiments, we set the minimum quorum threshold to 7% within the upstream regions and the maximum support threshold to 30% in the ORF regions. We use the shuffling program from SMILE [14] to compute the Z-scores. The shufffing program randomly shuffles the original input sequences to obtain a new *shuffled *set of sequences.

Then it computes, for each extracted frequent motif, its support (*π*) and weighted support (*π*_*w*_) in the shuffled set. For a given frequent motif ℳ
 MathType@MTEF@5@5@+=feaafiart1ev1aaatCvAUfKttLearuWrP9MDH5MBPbIqV92AaeXatLxBI9gBamrtHrhAL1wy0L2yHvtyaeHbnfgDOvwBHrxAJfwnaebbnrfifHhDYfgasaacH8akY=wiFfYdH8Gipec8Eeeu0xXdbba9frFj0=OqFfea0dXdd9vqai=hGuQ8kuc9pgc9s8qqaq=dirpe0xb9q8qiLsFr0=vr0=vr0dc8meaabaqaciaacaGaaeqabaWaaeGaeaaakeaaimaacqWFZestaaa@3790@, let *μ *and *σ *be the mean and standard deviation of its support across different sets (about 30) of shuffled sequences. Then the Z-score for each motif is calculated as: Z=π(ℳ)−μσ
 MathType@MTEF@5@5@+=feaafiart1ev1aaatCvAUfKttLearuWrP9MDH5MBPbIqV92AaeXatLxBI9gBamrtHrhAL1wy0L2yHvtyaeHbnfgDOvwBHrxAJfwnaebbnrfifHhDYfgasaacH8akY=wiFfYdH8Gipec8Eeeu0xXdbba9frFj0=OqFfea0dXdd9vqai=hGuQ8kuc9pgc9s8qqaq=dirpe0xb9q8qiLsFr0=vr0=vr0dc8meaabaqaciaacaGaaeqabaWaaeGaeaaakeaatuuDJXwAK1uy0HwmaeXbfv3ySLgzG0uy0Hgip5wzaGqbaiab=Lr8Ajabg2da9maalaaabaacciGae4hWdaNaeiikaGccdaGae03mH0KaeiykaKIaeyOeI0Iae4hVd0gabaGae43Wdmhaaaaa@4BE7@. Likewise we can also calculate the Z-score for each frequent motif by using the weighted support (which is also applicable for the repeated structured motif identification problem). As shown in Table 5, we can successfully predict GAL4, GAL4 chips, LEU3, PPR1 and PUT3 with the highest rank. CAT8 and LYS also have high ranks. We were thus able to extract all eight transcription factors for the Zinc factors with high confidence. As a comparison, with the same dataset RISO can only predict GAL4, LEU3 and PPR1.

**Table 5:** Regulons of Zn cluster proteins.

TF Name	Known Motif	Predicted Motifs	Num-Motifs	Ranking
GAL4 GAL4 chips	CGGRnnRCYnYnCnCCG	CGG[11,11]CCG	1634(3346)	1/1
CAT8	CGGnnnnnnGGA	CGG[6,6]GGA	1621(3356)	147/13
HAP1	CGGnnnTAnCGGCGGnnnTAnCGGnnnTA	CGG[6,6]CGG	1621(3356)	111/146
LEU3	RCCGGnnCCGGY	CCG[4,4]CGG	1588(3366)	2/1
LYS	WWWTCCRnYGGAWWW	TCC[3,3]GGA	1605(3360)	33/21
PPR1	WYCGGnnWWYKCCGAW	CGG[6,6]CCG	1621(3356)	1/2
PUT3	YCGGnAnGCGnAnnnCCGA CGGnAnGCnAnnnCCGA	CGG[10,11]CCG	727(4035)	1/1

TF Name stands for transcription factor name; Known Motif stands for the known binding sites corresponding to the transcription factors in TF Name column; Predicted Motifs stands for the motifs predicted by EXMOTIF; Num-Motifs gives the final (original) number of motifs extracted (final is after pruning those motifs that are also frequent in the ORF regions); Ranking stands for the Z-score ranking based on support/weighted support.

#### Discovery of composite regulatory patterns

The complex transcriptional regulatory network in Eukaryotic organisms usually requires interactions of multiple transcription factors. A potential application of EXMOTIF is to extract such composite regulatory binding sites from DNA sequences. We took two such transcription factors, URS1H and UASH, which are involved in early meiotic expression during sporulation, and that are known to cooperatively regulate 11 yeast genes [24]. These 11 genes are also listed in SCPD [1], the promoter database of *Saccharomyces cerevisiae*. In 10 of those genes the URS1H binding site appears downstream from UASH; in the remaining one (HOP1) the binding sites are reversed. We took the binding sites for the 10 genes (all except HOP1), and after their multiple alignment, we obtained their consensus:  taTTTtGGAGTaata[4,179]ttGGCGGCTAA (the lower case letters are less conserved, whereas uppercase letters are the most conserved). Table 6 shows the binding sites for UASH and URS1H for the 10 genes, their start positions, their alignment, and the consensus pattern. The gap between the sites are obtained after subtracting the length of UASH, 15, from the position difference (since the start position of UASH is given). The smallest gap is *l *= 119 - 110 - 15 = 4 and the largest is *u *= 288 - 94 - 15 = 179. Based on the on most conserved parts of the consensus, we formed the composite motif template: T
 MathType@MTEF@5@5@+=feaafiart1ev1aaatCvAUfKttLearuWrP9MDH5MBPbIqV92AaeXatLxBI9gBamrtHrhAL1wy0L2yHvtyaeHbnfgDOvwBHrxAJfwnaebbnrfifHhDYfgasaacH8akY=wiFfYdH8Gipec8Eeeu0xXdbba9frFj0=OqFfea0dXdd9vqai=hGuQ8kuc9pgc9s8qqaq=dirpe0xb9q8qiLsFr0=vr0=vr0dc8meaabaqaciaacaGaaeqabaWaaeGaeaaakeaaimaacqWFtepvaaa@3847@ = NNN[1,1]NNNNN[10,185]NNNNNNNNN (note the 6 additional gaps added to [4,179] to account for the non-conserved positions). We then extracted the structured motifs in the upstream regions of the 10 genes. We used the -800 to -1 upstream regions, and truncated the segment if it overlaps with an upstream ORF. The numbers of substitutions for NNN, NNNNN and NNNNNNNNN were set to *ε*_1 _= 1, *ε*_2 _= 2 and *ε*_3 _= 1, respectively. The quorum thresholds was set to *q *= 0.7 with the upstreams, and the maximum support within genes was set to 0.1% The rank of the true motif TTT[1,1]GGAGT[10,185]GGCGGCTAA was 290 (out of 5284 final motifs) with a Z-score of 22.61.

**Table 6:** UASH and URS1H binding sites.

**Genes**	**UASH**		**URS1H**		**Gap**
		
	Site	Pos	Site	Pos	
ZIP1	GATTCGGAAGTAAAA	-42	==TCGGCGGCTAAAT	-22	5
MEI4	TCTTTCGGAGTCATA	-121	==TGGGCGGCTAAAT	-98	8
DMC1	TTGTGTGGAGAGATA	-175	AAATAGCCGCCCA==	-143	17
SPO13	TAATTAGGAGTATAT	-119	AAATAGCCGCCGA==	-100	4
MER1	GGTTTTGTAGTTCTA	-152	TTTTAGCCGCCGA==	-115	22
SPO16	CATTGTGATGTATTT	-201	==TGGGCGGCTAAAA	-90	96
REC104	CAATTTGGAGTAGGC	-182	==TTGGCGGCTATTT	-93	74
RED1	ATTTCTGGAGATATC	-355	==TCAGCGGCTAAAT	-167	173
REC114	GATTTTGTAGGAATA	-288	==TGGGCGGCTAACT	-94	179
MEK1	TCATTTGTAGTTTAT	-233	==ATGGCGGCTAAAT	-150	68

Consensus	taTTTtGGAGTaata		==ttGGCGGCTAA==		[4,179]

## Conclusion and future work

In this paper, we introduced EXMOTIF, an efficient algorithm to extract structured motifs within one or multiple biological sequences. We showed its application in discovering single/composite regulatory binding sites. In the structured motif template, we assume the gap range between each pair of simple motifs is known. In the future, we plan to solve the motif discovery problem when even the gap ranges are unknown. Another potential direction is to directly extract structured profile (or position weight matrix) patterns.

## Authors' contributions

All authors contributed equally to this work.
